# Synthesis and Characterization of *N,N,O*-Tridentate Aminophenolate Zinc Complexes and Their Catalysis in the Ring-Opening Polymerization of Lactides

**DOI:** 10.3389/fchem.2019.00189

**Published:** 2019-04-05

**Authors:** Wei-Yi Lu, Kuo-Hui Wu, Hsuan-Ying Chen, Chu-Chieh Lin

**Affiliations:** ^1^Department of Chemistry, National Chung Hsing University, Taichung, Taiwan; ^2^Department of Chemistry, Graduate School of Science, The University of Tokyo, Tokyo, Japan; ^3^Department of Medicinal and Applied Chemistry, Kaohsiung Medical University, Kaohsiung, Taiwan; ^4^Department of Medical Research, Kaohsiung Medical University Hospital, Kaohsiung, Taiwan

**Keywords:** zinc, polymerization, catalyst, lactide, kinetic

## Abstract

A series of aminophenolate ligands with various pendant groups and associated ethyl Zn complexes were synthesized and studied as catalysts for the ring-opening polymerization (ROP) of lactides (LAs). The thiophenylmethyl group (**L**^**4**^**ZnEt**) increased the catalytic activity more than the benzyl group (**L**^**1**^**ZnEt**) did, and 2-fluorobenzyl (**L**^**3**^**ZnEt**) and 2-methoxybenzyl (**L**^**2**^**ZnEt**) groups had the opposite effect. In addition, the LA polymerization mechanism proved by Nuclear Magnetic Resonance and Density Function Theory was that LA was attracted by H···O bond of an α-hydrogen of the LA molecule and the phenoxyl oxygen of the catalyst. After the dissociation of amino group from the Zn atom, the benzyl alcohol initiated LA without replacing the ethyl group of Zn complex. It is the first case where the ethyl group is regarded as a ligand and cannot be replaced by benzyl alcohol, and this information is very important for the mechanism study of ROP.

## Introduction

**Figure 1:**
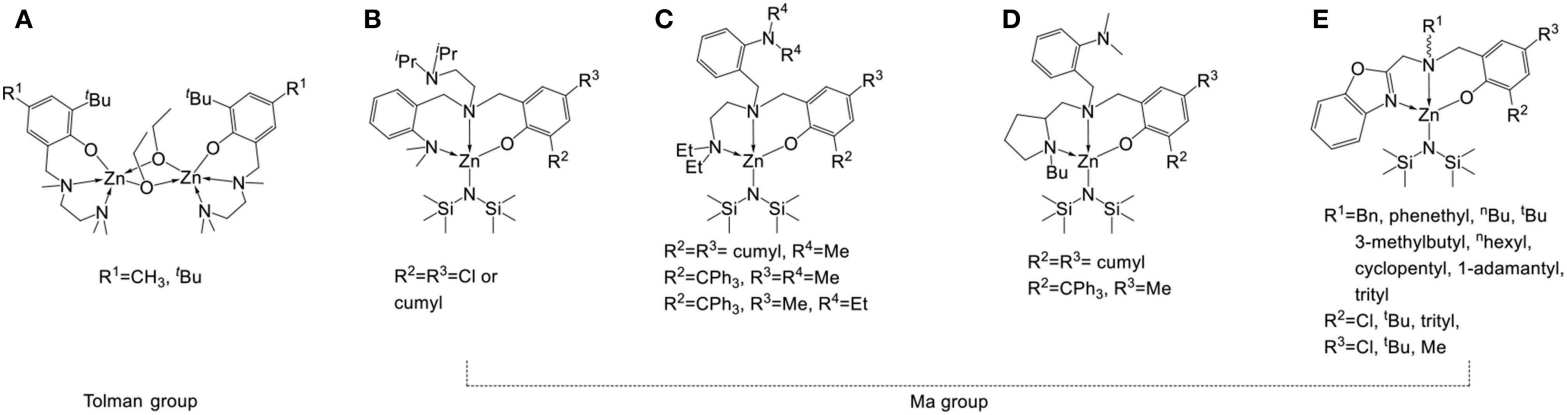
Zn complexes bearing tridentate (**A**; Williams et al., 2003), (**E**; Hu et al., 2018), and tetradentate (**B–D**; Yang et al., 2015) aminophenolate ligands.

Because petrochemical polymers such as polystyrene, polypropylene, polyethylene, and poly(vinyl chloride) can be produced easily and cheaply, they are widely used as disposable packaging materials. Because these petrochemical polymers need more than a hundred years to degrade into innocuous soil manure (Rochman et al., 2013), polymer pollution has become a serious problem (Romer, 2010; Romer and Tamminen, 2014; Ladewig et al., 2015; Zhang et al., 2017). The replacement of non-biodegradable polymers with biodegradable materials is therefore a popular field of research (Levis and Barlaz, 2011). Poly(lactide) (PLA) is a biopolymer designed to ameliorate pollution by petrochemical plastics. Owing to its biodegradability, biocompatibility, and permeability, PLA is commonly used for various purposes, such as humidity detection (Sun et al., 2007), MRI contrast agents (Patel et al., 2008), cell/tissue anti-adhesion (Lih et al., 2015), nanocomposites (Raquez et al., 2013), drug delivery (Khemtong et al., 2009), blood circulation (Ma et al., 2008), bone replacement (Simpson et al., 2007), and tissue engineering (Place et al., 2009). Ring-opening polymerization (ROP) by using metal complexes (Bellemin and Dagorne, 2014; Guillaume et al., 2015; Sarazin and Carpentier, 2015; Huang et al., 2016; Fuoco and Pappalardo, 2017; Redshaw, 2017) as catalysts is a common method for the efficient synthesis of PLA. Because no cytotoxic metal residue is required in PLA for the biomaterials, the use of a non-cytotoxic metal such as zinc (Williams et al., 2003; Romain and Williams, 2014; Yang et al., 2015; Binda et al., 2016; Ebrahimi et al., 2016; Thevenon et al., 2016; Wang et al., 2016) in lactide polymerization has been investigated widely. Ligands are crucial for the catalyst design because their catalytic activity can be increased. In a study of Zn complexes (Williams et al., 2003) bearing tridentate aminophenolate ligands, a high catalytic activity was observed during *rac*-lactide (*rac*-LA) polymerization, as shown in Figure 1A. As in previous studies, the tetradentate aminophenol ligands reacted with Zn[N(SiMe_3_)_2_]_2_ and four coordinated Zn complexes with a non-coordinated fourth amino group were obtained, as shown in Figures 1B–D. According to the polymerization results of Figures 1B–E, the steric bulky substituents on the phenolate ring, the fourth coordinated amino groups, and chiral *N*-alkyl groups can increase the stereoselectivity of *rac*-LA polymerization, and maintain high catalytic activity. A survey of the coordination behavior of Zn complexes (Gao et al., 2013) revealed that five and six are the possible coordination numbers for these complexes, and even seven-coordinated Zn complexes were reported (Vaiana et al., 2007). It would be worthwhile to design the fourth coordinated group of tetradentate aminophenol ligands to interact with the Zn atom, and influence their catalytic activities. In this study, a series of aminophenol ligands and associated Zn complexes were synthesized, and their application in LAs polymerization was investigated.

## Results and Discussion

### Syntheses and Characterization

**Figure 2:**
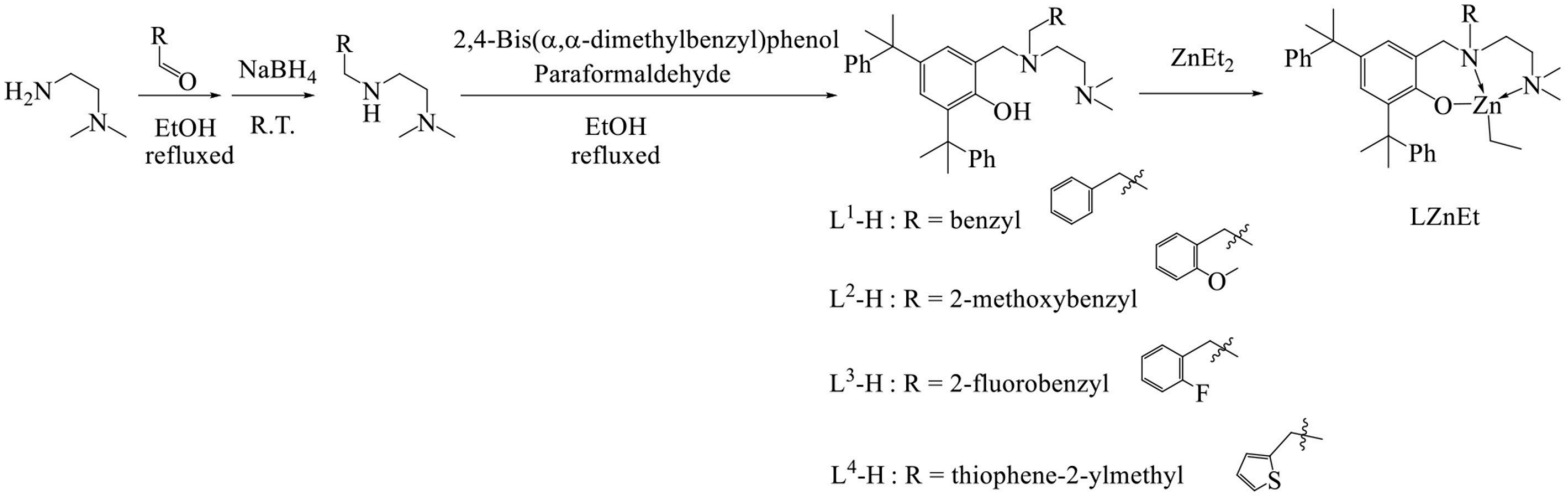
Synthesis of the ligands L^1^-H–L^4^-H and associated Zn complexes.

A series of *N*^1^-alkyl- *N*^2^,*N*^2^-dimethylethylene-1,2-diamines was synthesized by using NaBH_4_ to reduce 2-alkylideneamino-*N*,*N*-dimethylethylen-1-amines that were synthesized by condensing the aldehyde derivatives with dimethylethylenediamine in ethanol. All ligands L^1^-H–L^4^-H were prepared by refluxing a mixture of *N*^1^-alkyl- *N*^2^,*N*^2^-dimethylethylene-1,2-diamine, *para*-formaldehyde, and 2,4-bis(α,α-dimethylbenzyl)-phenol (Figure 2). All the ligands reacted with 1.1 equivalents of ZnEt_2_ in THF at 0°C to produce a moderate yield (74–83%) of Zn compounds after hexane washing. The Zn complex synthesis can be identified by the ^1^H NMR spectrum. The peaks of the methylene groups of HO[(^t^Bu)_2_-Ph]**C*H***_**2**_N and N**C*H***_**2**_Ar from the ^1^H NMR spectrum are singlet in ligands and doublet of doublets in Zn complexes, and the proton of PhO-***H*** (10.43–10.15 ppm) disappeared after ZnEt_2_ was added. The formulas and structures of the compounds were confirmed on the basis of ^1^H and ^13^C Nuclear Magnetic Resonance (NMR) spectra.

**Figure 3:**
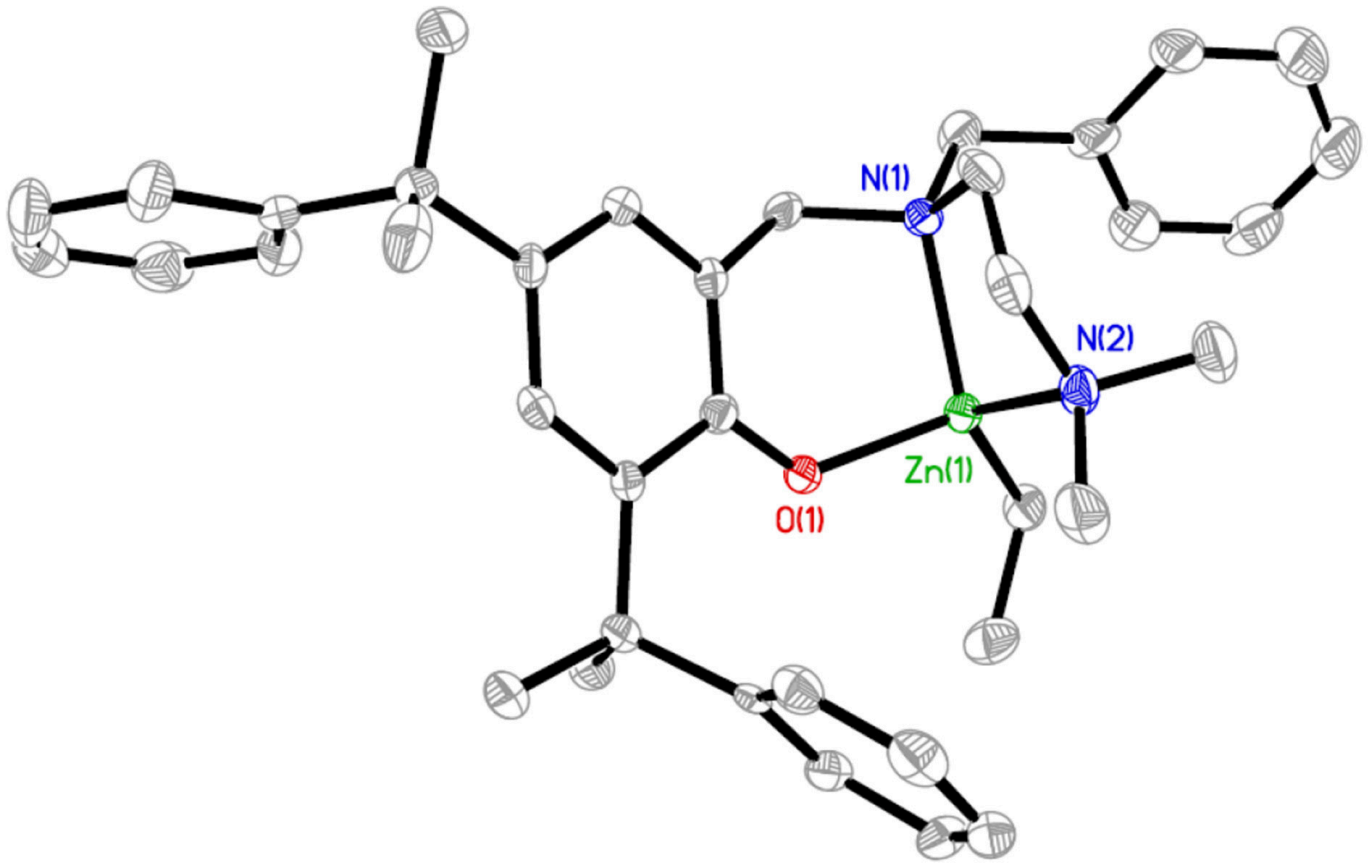
Molecular structure of **L**^**1**^**ZnEt** (ellipsoids drawn at the 50% probability, H atoms omitted for clarity). Selected bond lengths (Å) and angles (degrees): Zn(1)-O(1), 1.9583(18); Zn(1)-N(1), 2.159(2); Zn(1)-N(2), 2.121(2); Zn(1)-C(37), 1.983(3); O(1)-Zn(1)-N(1), 95.61(8); O(1)-Zn(1)-N(2), 102.08(9); O(1)-Zn(1)-C(37), 114.38(10); C(37)-Zn(1)-N(1), 134.15(11); C(37)-Zn(1)-N(2), 119.88(11); N(2)-Zn(1)-N(1), 83.55(9).

The X-ray structure of **L**^**1**^**ZnEt** (CCDC 1574177), Figure 3 revealed a four-coordinated, mononuclear Zn complex with one ethyl group and one aminophenolate ligand. In structural chemistry, the index for four-coordinated complexes (τ_4_) is the number that indicates the geometry of the coordination center (Yang et al., 2007). **L**^**1**^**ZnEt** showed an intermediate structure varying in geometry between trigonal pyramidal and seesaw (C_2v_, θ_6_ = 90°), with the corresponding τ_4_ value being 0.74. The distances between the Zn(1) atom and C(37) O(1), N(1), and N(2) atoms were 1.983(3), 1.9583(18), 2.159(2), and 2.121(2) Å, respectively.

### Ring Opening Polymerization of LAs

The catalytic activity of Zn complexes for *L*-LA and *rac*-LA polymerizations with benzyl alcohol (BnOH) as the initiator under a nitrogen atmosphere was investigated, and the results are given in Table 1. As shown by entries 1–4 in Table 1, all Zn complexes were active for *L*-LA polymerization at 25°C, producing polymers with narrow polydispersity indexes (Ð, 1.08–1.13). **L**^**4**^**ZnEt** had the most controllability of the polymer molar mass with similar values of *Mn*_cal_, *Mn*_NMR_, and *Mn*_GPC_. However, the difference of catalytic activity of these Zn complexes was initially unclear. To determine the difference, the polymerization temperature was set at 100°C and polymerization was terminated after 2 h (entries 5–8 in Table 1). The catalytic activity of *L*-LA polymerization was in the following order: **L**^**4**^**ZnEt** > **L**^**1**^**ZnEt** > **L**^**3**^**ZnEt** > **L**^**2**^**ZnEt**. The results revealed that the thiophenylmethyl group of **L**^**4**^**ZnEt** increased the catalytic activity of Zn complexes more than the benzyl group of **L**^**1**^**ZnEt** did, and that 2-fluorobenzyl and 2-methoxybenzyl groups decreased the catalytic activity of the complexes. From the polymer data (entries 1–8 in Table 1), the values of *Mn*_NMR_, and *Mn*_GPC_ were smaller than that of *Mn*_cal_, and this phenomenon may be attributed to the transesterification. As shown by entries 9–12 in Table 1, the catalytic rates for *rac*-LA polymerization were the same as those for *L*-LA polymerization. The *rac*-PLA polymerized by **L**^**4**^**ZnEt** showed the highest selectivity. According to the literature, electronic donating substituents increased the catalytic activity of Zn catalysts (Chen et al., 2006, 2011, 2015; Huang et al., 2006; Chuang et al., 2011; Fliedel et al., 2014a,b). In our case, the pendant-chelating substituents do not coordinate with the Zn atom in the solid state, but they increase the catalytic activity. Crystal data imply that the fourth coordinated groups, such as thiophenylmethyl, 2-methoxybenzyl, and 2-fluorobenzyl groups, still do not coordinate to Zn atom, just like the behavior of the Zn complexes (1B-1D) shown in Figure 1; however, the low stereoselectivity of *rac*-LA polymerization was also observed in our case. These phenomena are ascribed, possibly, to the size of the pendant substituents. The reasons will be discussed with DFT results later. In addition, the Ð values of these *rac*-PLAs were higher than that of PLAs (Table 1), and it may be attributed to more transesterification at a higher temperature in longer polymerization time.

**Table 1:** Rinrg opening polymerizations of *L*-LA and *rac*-LA by Zn complexes.

**Entry**	**Cat**.	**[M]:[cat.]:[BnOH]**	**Time(h)**	**Conv. (%)^e^**	***Mn*_cal._^d^**	***Mn*_NMR_^e^**	***Mn*_GPC_^f^**	**Ð^f^**	**P*r*^e^**
1^a^	L^1^ZnEt	50:1:1	6.5	89	6,500	4,600	4,400	1.08	
2^a^	L^2^ZnEt	50:1:1	8	91	6,700	5,600	4,900	1.10	
3^a^	L^3^ZnEt	50:1:1	7.5	90	6,600	5,300	4,400	1.09	
4^a^	L^4^ZnEt	50:1:1	7	96	7,000	6,800	6,300	1.13	
5^b^	L^1^ZnEt	50:1:1	2	81	5,900	4,300	3,800	1.10	
6^b^	L^2^ZnEt	50:1:1	2	68	5,000	3,000	2,900	1.11	
7^b^	L^3^ZnEt	50:1:1	2	74	5,500	3,800	3,600	1.10	
8^b^	L^4^ZnEt	50:1:1	2	93	6,800	5,000	4,500	1.10	
9^c^	L^1^ZnEt	50:1:1	3.5	95	6,900	7,300	5,400	1.46	0.52
10^c^	L^2^ZnEt	50:1:1	3.5	86	6,300	6,900	5,300	1.28	0.55
11^c^	L^3^ZnEt	50:1:1	3.5	93	6,800	7,300	5,600	1.39	0.49
12^c^	L^4^ZnEt	50:1:1	3.5	98	7,200	7,500	5,300	1.50	0.58

a *Reaction conditions: [L-LA]_0_ = 0.5 M, toluene 10 mL, room temperature, in N_2_*.

b *Reaction conditions: [L-LA]_0_ = 0.5 M, toluene 10 mL, 100°C, in N_2_*.

c *Reaction conditions: [rac-LA]_0_ = 0.5 M, toluene 10 mL, 100°C, in N_2_*.

d *Calculated from the molar mass of M_w_(LA) × [M]_0_/[BnOH]_0_ × conversion + M_w_(BnOH)*.

e *Obtained from the ^1^H NMR analysis*.

f *Obtained from GPC analysis and calibrated by polystyrene standard. Values are obtained from GPC times 0.58*.

**Figure 4:**
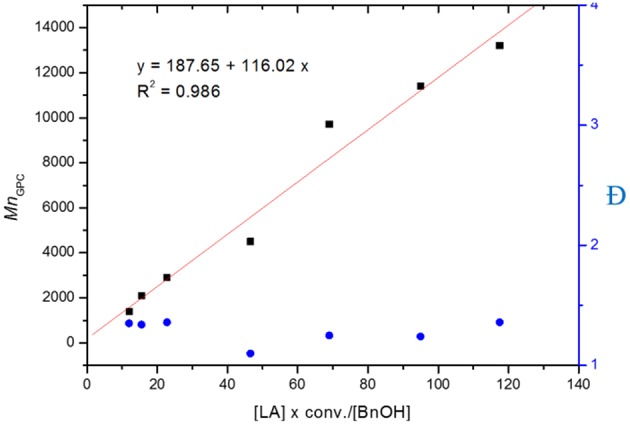
Linear plot of *Mn*_GPC_ vs. [LA] × conv. / [BnOH], with Ðindicated by blue dots (entries 1–7 in Table 2).

The controllability of polymer molar mass by using **L**^**4**^**ZnEt** as a catalyst was investigated, and the results are given in Table 2. The linear relationship between *Mn*_GPC_ and ([LA] × conv.)/[BnOH] exhibited in Figure 4 shows that the polymerization of LA by using **L**^**4**^**ZnEt** as a catalyst was highly controllable with acceptable Ð values.

**Table 2:** Ring opening polymerization of *L*-lactide by L^4^ZnEt with BnOH.

**Entry**	**[M]_**0**_:[cat.]_**0**_: [BnOH]_**0**_**	**Time (h)**	**Conv. (%)**	***Mn*_cal._^e^**	***Mn*_NMR_^f^**	***Mn*_GPC_^g^**	**Ð^g^**
1^a^	50:1:1	2	93	6,800	5,000	4,500	1.10
2^b^	75:1:1	2	92	10,100	7,200	9,700	1.25
3^c^	100:1:1	2.5	95	13,700	10,200	11,400	1.24
4^d^	125:1:1	2	94	16,900	12,300	13,200	1.36
5^a^	50:1:2	1	91	3,400	3,300	2,900	1.36
6^a^	50:1:3	1	93	2,300	2,400	2,100	1.34
7^a^	50:1:4	1	96	1,800	1,800	1,400	1.35

a *Reaction conditions: [L-LA]_0_ = 0.50 M, toluene 10 mL, 100°C, in N_2_*.

b *Reaction conditions: [L-LA]_0_ = 0.75 M, toluene 10 mL, 100°C, in N_2_*.

c *Reaction conditions: [L-LA]_0_ = 0.10 M, toluene 10 mL, 100°C, in N_2_*.

d *Reaction conditions: [L-LA]_0_ = 1.25 M, toluene 10 mL, 100°C, in N_2_*.

e *Calculated from the molar mass of M_w_(LA) × [M]_0_/[BnOH]_0_ × conversion + M_w_(BnOH)*.

f *Obtained from the ^1^H NMR analysis*.

g *Obtained from GPC analysis and calibrated by polystyrene standard. Values are obtained from GPC times 0.58*.

### Kinetic Studies of Polymerization of L-LA by Using L^3^ZnEt

**Figure 5:**
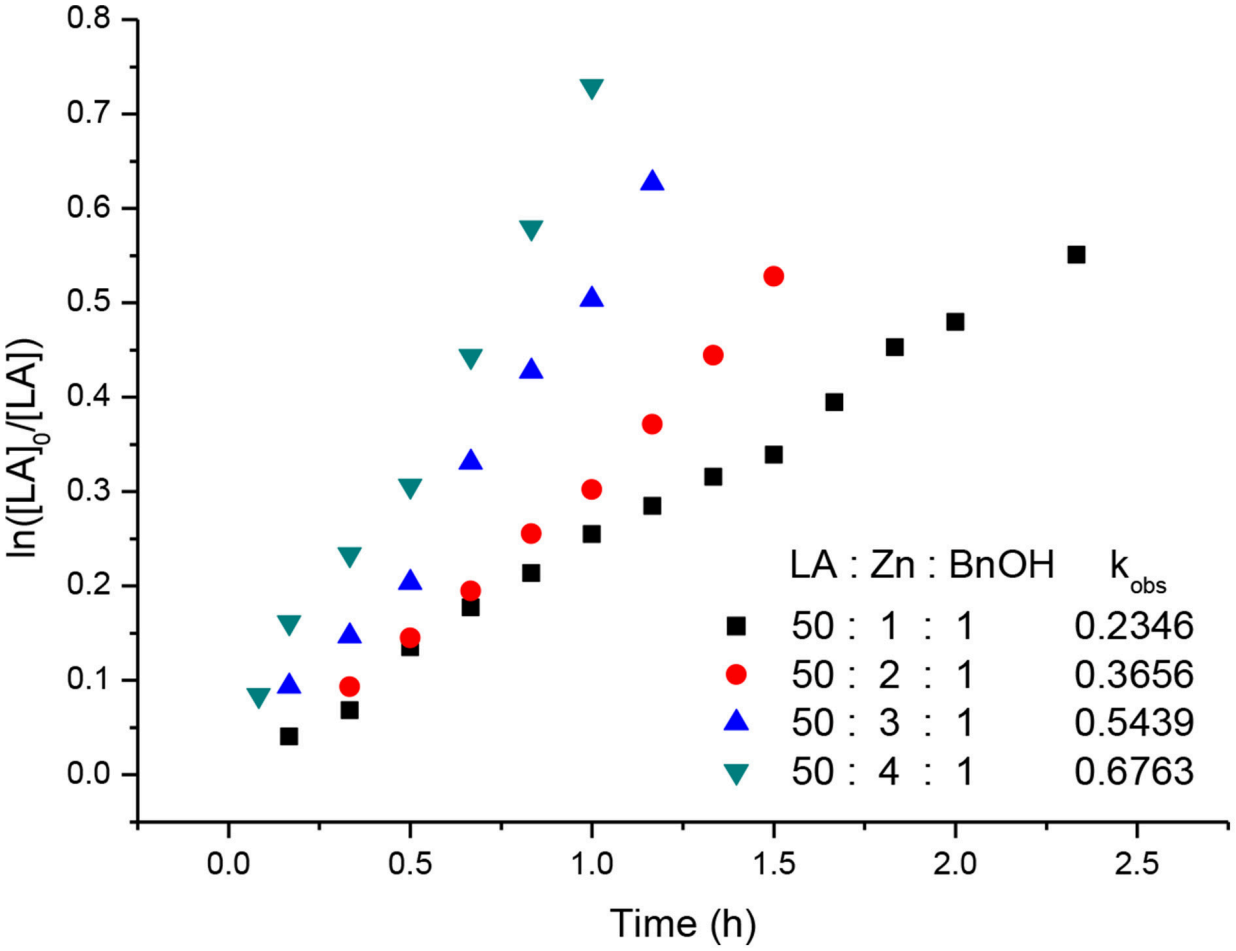
First-order kinetic plots for *L*-LA polymerizations vs. time in toluene (10 mL) at 25°C with various concentrations of complex [**L**^**3**^**ZnEt**].

**Figure 6:**
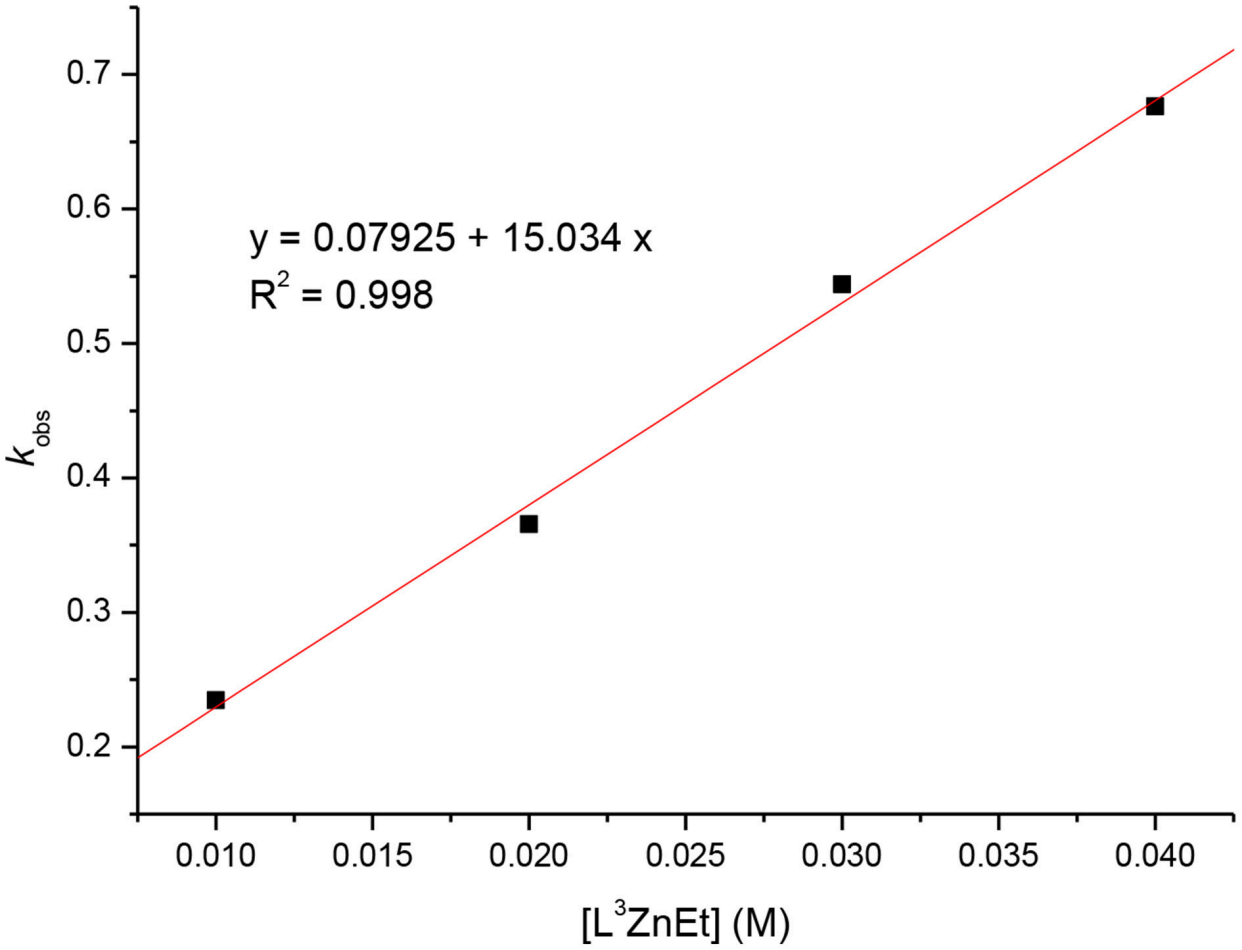
Linear plot of *k*_obs_ vs. [**L**^**3**^**ZnEt**] for the polymerization of *L*-LA with [*L*-LA] = 0.5 M in toluene (10 mL) at 25°C.

**Figure 7:**
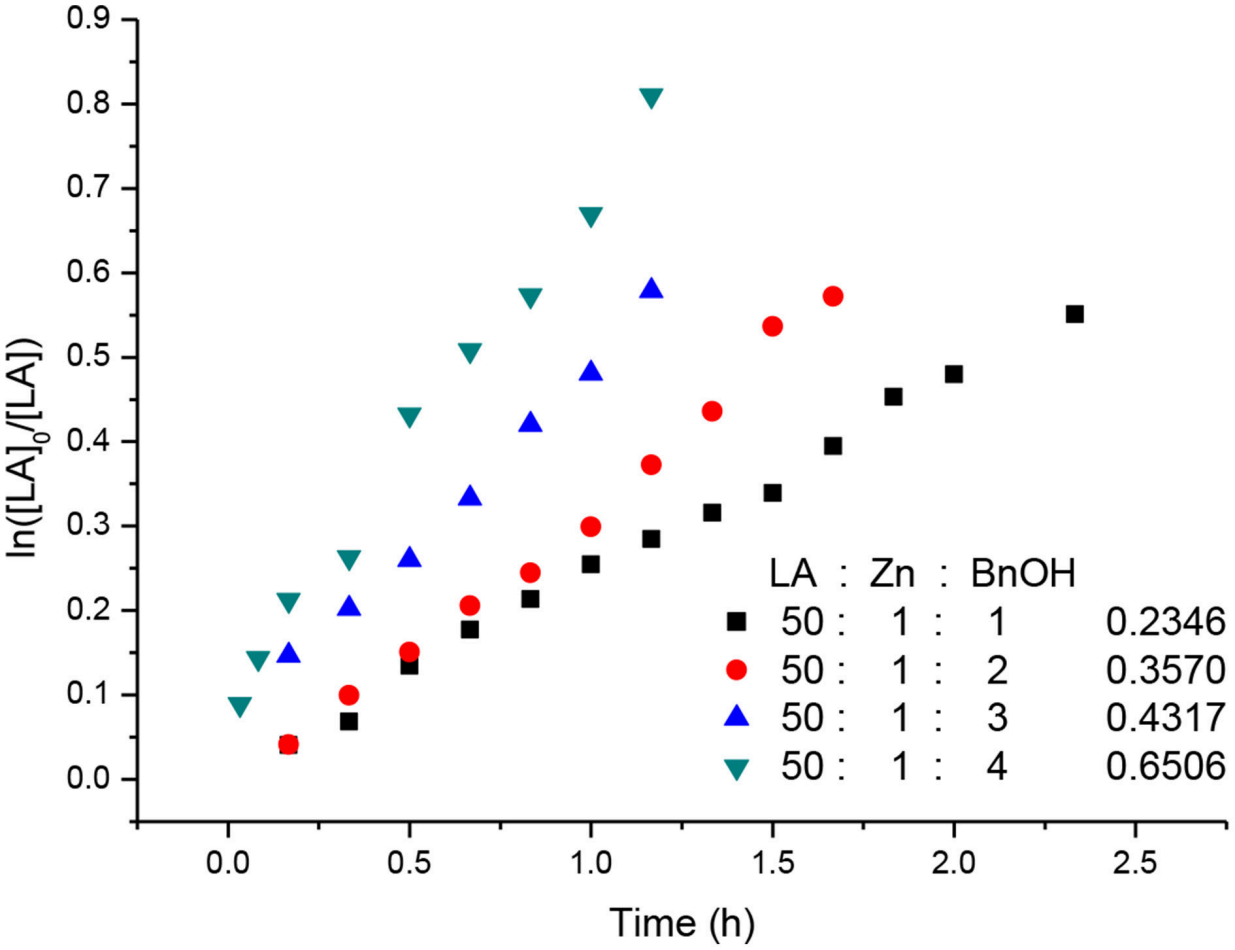
First-order kinetic plots for *L*-LA polymerizations vs. time in toluene (10 mL) at 25°C with various concentrations of [BnOH].

**Figure 8:**
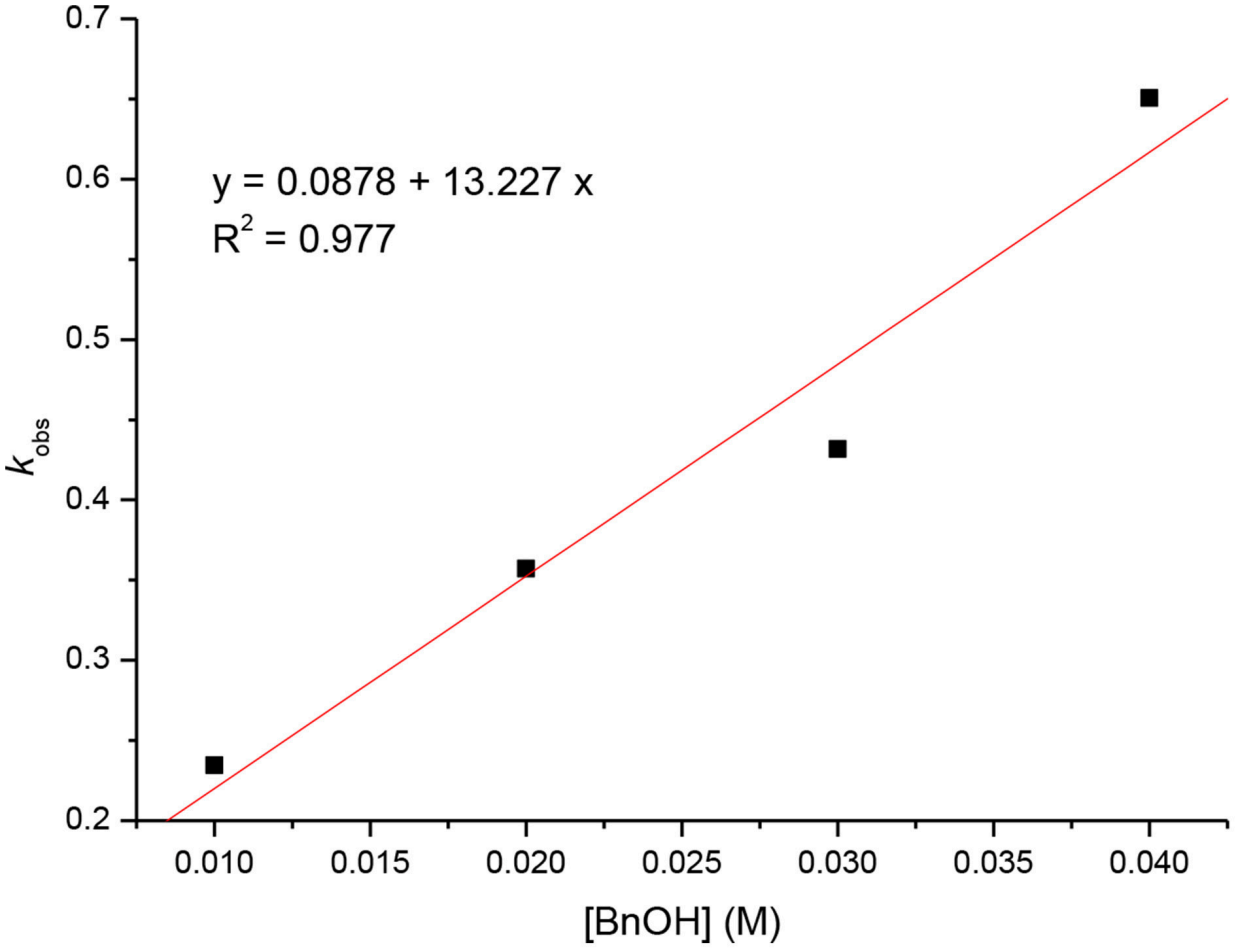
Linear plot of *k*_obs_ vs. [BnOH] for the polymerization of *L*-LA with [*L*-LA] = 0.5 M in toluene (10 mL) at 25°C.

Kinetic studies of the polymerization of *L*-LA catalyzed by **L**^**3**^**ZnEt** in the presence of BnOH were performed to establish the reaction order with respect to [*L*-LA], [**L**^**3**^**ZnEt**], and [BnOH]. The experiments were performed at [*L*-LA]_0_/[**L**^**3**^**ZnEt**]/[BnOH] ratios of 50:1:1, 50:1:2, 50:1:3, 50:1:4, 50:2:1, 50:3:1, and 50:4:1 ([*L*-LA] = 0.5 M in 10 mL toluene at 25°C), respectively. The preliminary results indicated that the reaction rate was a first-order dependent on [*L*-LA] for all seven ratios (Figures 5, 7) according to Equation (1), where *k*_obs_ = *k*_prop_[**L**^**3**^**ZnEt**]^*x*^[BnOH]^*y*^ and *k*_prop_ is the propagation rate constant. To determine the order (*x*) of [**L**^**3**^**ZnEt**], different [**L**^**3**^**ZnEt**] (10, 20, 30, and 40 mM) with the same [*L*-LA] (0.5 M) and [BnOH] (10 mM) were used (Figure 6). In addition, [BnOH] is regarded as a constant and incorporated into *k*_1_ (*k*_1_ = *k*_prop_[BnOH]^*y*^). The variable *k*_obs_ is first assumed to be as described by Equation (2). When *k*_obs_ is plotted against [**L**^**3**^**ZnEt**], a *k*_1_ values of 15.034 (M^−1^h^−1^) for *x* = 1 is obtained (Figure 6). The variable *k*_obs_ is then assumed to be as described by Equation **(**3), where [**L**^**3**^**ZnEt**] is regarded as a constant and incorporated into *k*_2_. Furthermore, various concentrations of [BnOH] (10, 20, 30, and 40 mM) with the same [*L*-LA] (0.5 M) and [**L**^**3**^**ZnEt**] (10 mM) (Figure 7) are used. When *k*_obs_ is plotted against [BnOH], a *k*_2_ values of 13.227 (M^−1^h^−1^) for *y* = 1 is obtained (Figure 8). Next, *k*_prop_ is calculated to be 1,413 by averaging *k*_1_/[BnOH] and *k*_2_/[**L**^**3**^**ZnEt**]. *L*-LA polymerization using [**L**^**3**^**ZnEt**] and [BnOH] followed by an overall kinetic law given by Equation (4).

(1) $$-d[\text{L}-\text{LA}]/\text{d}t=k_{\text{obs}}[L-\text{LA}]$$

(2) $$k_{\text{obs}}=k_{1}[{\text{L}}^{3}\text{ZnEt}{]}^{\text{x}}$$

(3) $$k_{\text{obs}}=k_{2}[\text{BnOH}{]}^{\text{y}}$$

(4) $$-\text{d}[\text{L}-\text{LA}]/\text{dt}=1413[L-\text{LA}][L^{3}ZnEt][\text{BnOH}]$$

### Proposed Mechanism

**Figure 9:**
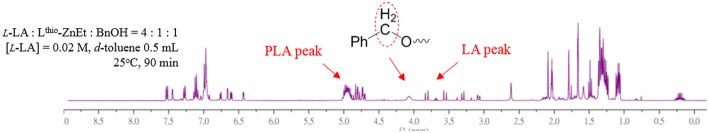
^1^H NMR spectra of the LA polymerization ([LA]:[Zn]:[BnOH]=4:1:1, [LA] = 0.02 M in *d*^8^-toluene (0.5 mL) at 25°C).

In order to realize the mechanism of LA polymerization by using Zn catalysts, the ^1^H NMR study of the reaction of **L**^**4**^**ZnEt** with one equivalent BnOH was investigated as shown in Figure S19. In Figure S19D, the ^1^H NMR spectrum revealed that **L**^**4**^**ZnEt** did not react with BnOH. This phenomenon was very surprising because most alkyl Zn complexes could react with alcohol to form Zn alkoxide complexes (Chuang et al., 2011; Fliedel et al., 2014a,b; Chen et al., 2015). To prove that the ethyl group of **L**^**4**^**ZnEt** could not be replaced by BnOH in polymerization process, the LA polymerization ([LA]:[Zn]:[BnOH] = 4:1:1, [LA] = 0.02 M in *d*^8^-toluene (0.5 mL) at 25°C) was monitored by ^1^H NMR as shown in Figure 9, Figure S20, and the ethyl group was always at 0.25 ppm from the beginning to the end of the polymerization (Figure S20). When these ^1^H NMR spectra revealed that the BnOH did not replace the ethyl group of the Zn complex in the LA polymerization process, we were curious about how BnOH initiated the monomer. To understand the polymerization mechanism, the interactions between the catalysts, the initiator, LA, and relative free energies of the intermediates of the catalytic reaction were studied using the DFT calculation.

### DFT Calculations: Mechanistic Study of LA Polymerization by Using Zn Complexes Bearing Aminophenolate Ligands as Catalysts

**Figure 10:**
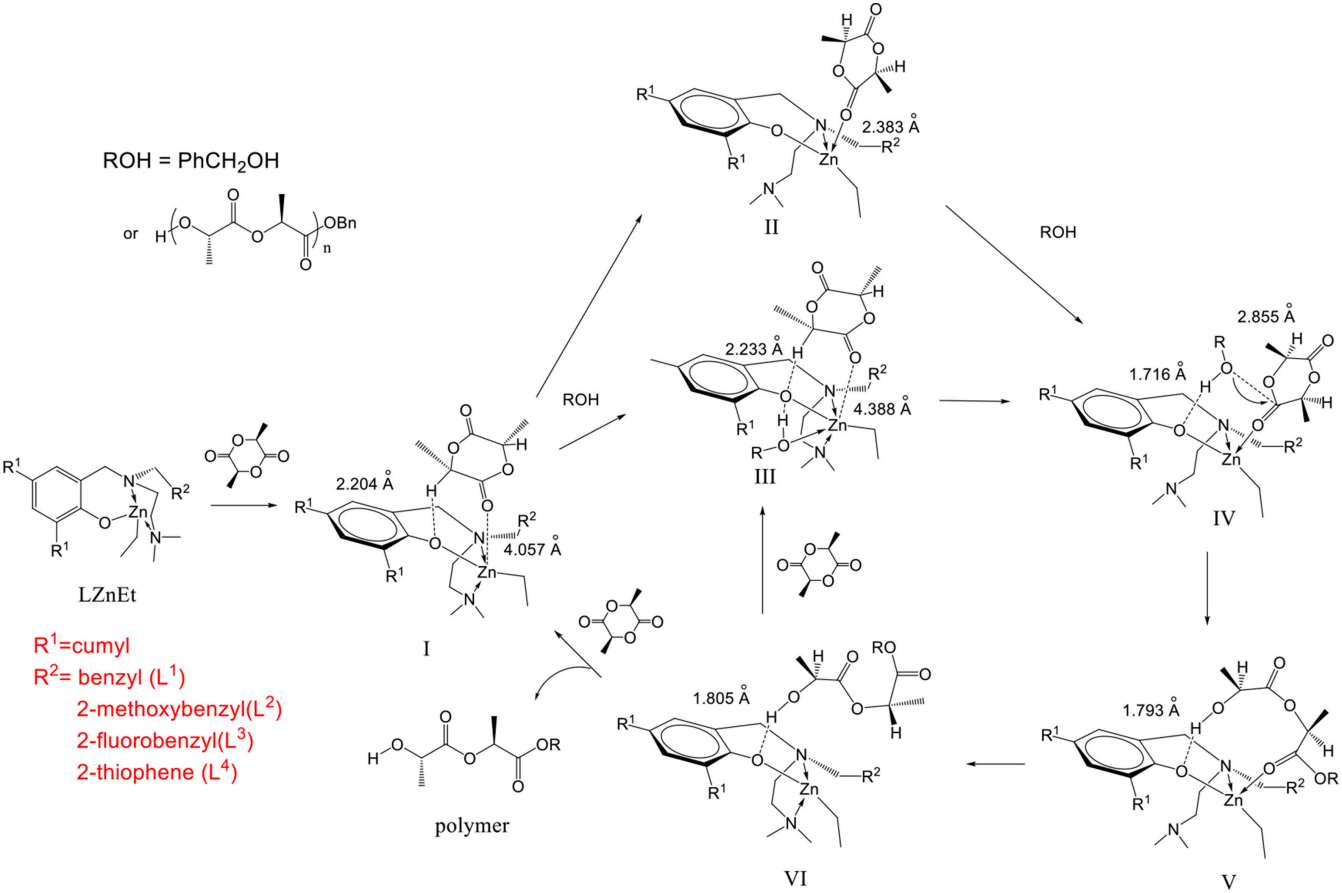
Mechanism of *L*-lactide polymerization with *N,N,O*-tridentate aminophenolate Zn complexes as catalysts and benzyl alcohol as an initiator.

In our DFT calculations, the cumyl group at the 4 position of the phenol ring **L**^**2**^**ZnEt** was simplified to a hydrogen atom, since it is far from the zinc catalytic center. The initiator was replaced by a methanol molecule because different alcohol molecules often show similar activity (Chang et al., 2015). The mechanism is shown in Figure 10.

**Figure 11:**
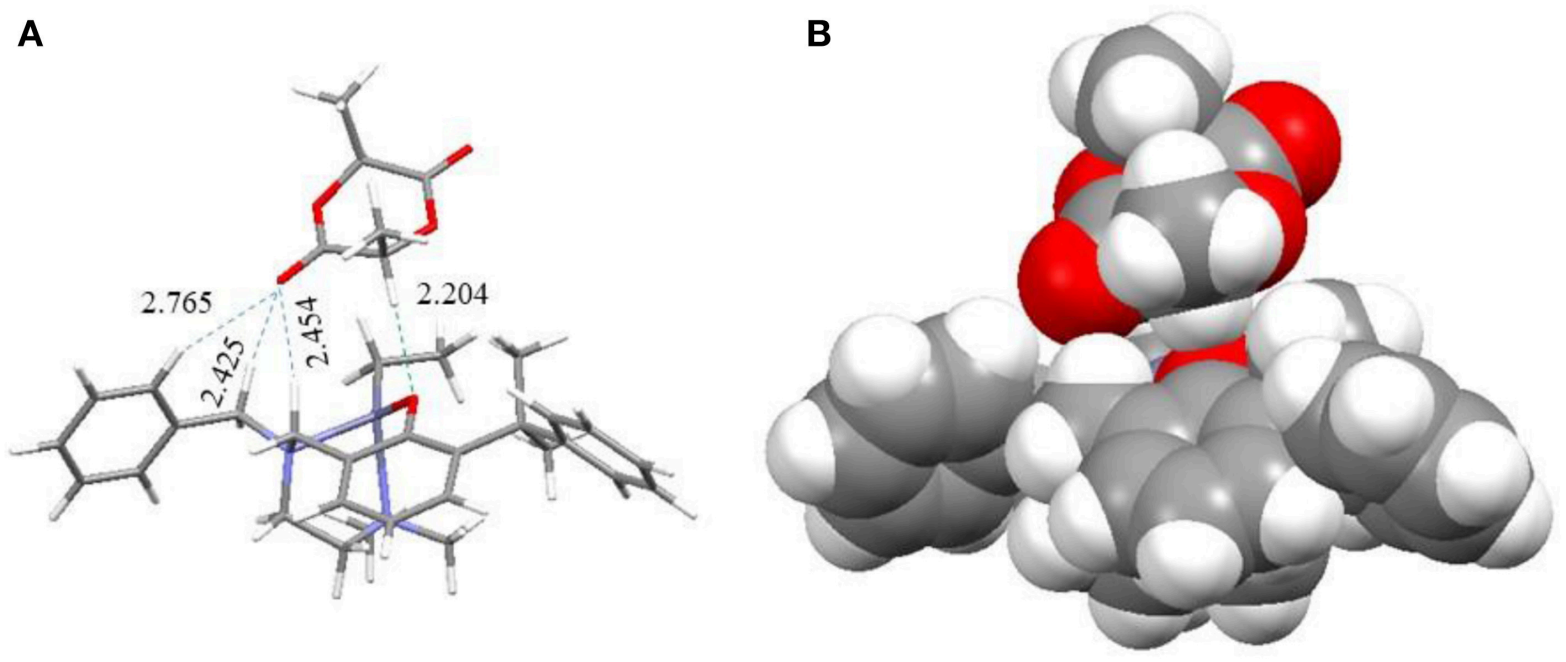
The stick **(A)** and spacefill **(B)** model of intermediate **I**.

When the catalyst reacted with LA, the LA molecule was initially stably bonded in an open pocket formed between the *R*^1^ group on the 2 position of the phenol ring and the pendant *R*^2^ group to form intermediate **I** as shown in Figure 11. The major interaction to stabilize this structure was found to be a unique hydrogen bond between an α-hydrogen of the LA molecule and the phenoxyl oxygen of the catalyst with a H···O distance of 2.204 Å. **I** then went through two possible pathways to form key catalytic intermediate **IV**. One of possible pathways is that the NMe_2_ group dissociated from the Zn center to produce an empty space to be coordinated by a carbonyl oxygen atom with a Zn-O bond (2.383 Å) to form intermediate **II**. After that, the initiator came into the complex to form intermediate **IV**. Another possible pathway is that the initiator (ROH) bonded to the Zn complex with a coordination bond between its oxygen atom and the Zn center (2.508 Å) and a hydrogen bond between the hydroxyl hydrogen atom of ROH and phenoxyl oxygen of catalyst to form intermediate **III**. The NMe_2_ group subsequently left the Zn atom to make a rearrangement to generate **IV**. In intermediate **IV**, the O atom of the initiator was close to the carbonyl group of LA with a distance of 2.855 Å to facilitate the ring opening reaction. Moreover, the hydrogen bond between the initiator and the phenoxyl O atom of Zn catalyst stabilized this transient intermediate structure (**IV**) and also activated the initiator. After the LA ring opening, a new hydroxyl group formed at one end of the polymer (or oligomer) and interacted with phenoxyl oxygen through a hydrogen bond with a coordination between the zinc center and the carbonyl group on the other end to form intermediate **V**. The NMe_2_ group then came back to re-coordinate on the zinc center to assist the leaving of the carbonyl group of the polymer to form intermediate **VI** and re-activated the catalyst. Finally, another LA monomer came in to reform intermediate **III** to continue the polymerization reaction or to expel the polymer from the catalyst to regenerate intermediate **I** to finish one catalytic cycle.

**Figure 12:**
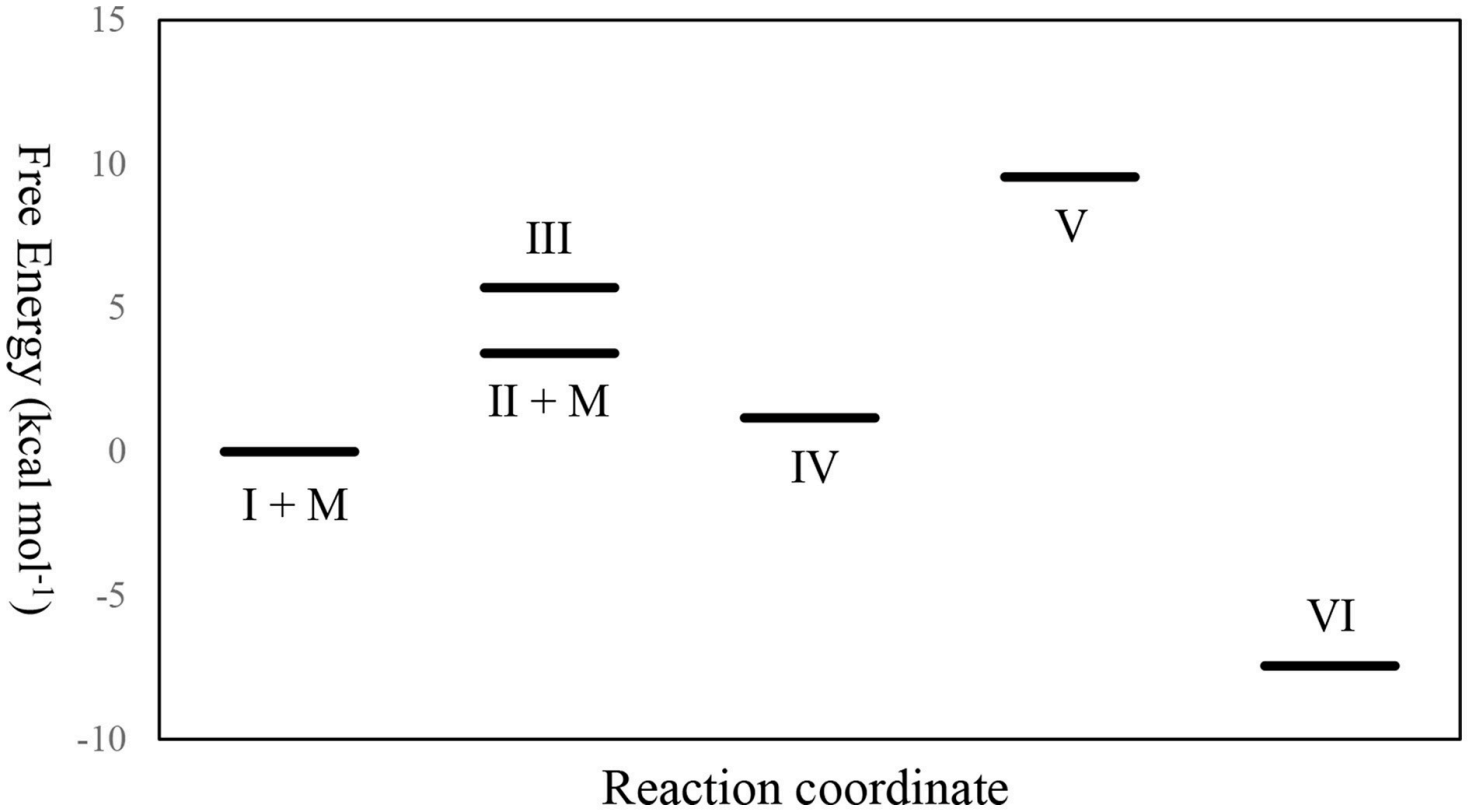
The relative free energy level diagram of all intermediates in the mechanism. M stands for methanol.

From the thermochemical data of our DFT studies as shown in Figure 12 and Table 3, it can be found that to generate **IV** from **I**, the pathway through **II** was a little more favored, since the relative free energy of **II** + MeOH was slightly lower than that of **III** (2.276 kcal mol^−1^ lower). This is because the molecular freedom (entropy) of **II** + methanol was much higher than that of **III**. After forming **IV**, the ring opening reaction to form **V** makes its relative free energy increase, due to the fact that the bulky polymer chain was restricted on the catalyst. However, after the carbonyl group dissociated from the zinc atom, the relative free energy of **VI** decreased dramatically. This large free energy decrease is a strong driving force to make the catalyst open the lactide ring.

**Table 3:** Thermochemical data of intermediates derived from DFT calculations^a^.

**Intermediate**	**Relative free energy (kcal mol^**−1**^)**	**Relative enthalpy (kcal mol^**−1**^)**	**Entropy (cal mol^**−1**^K^**−1**^)**
I + MeOH	0.000	0.000	330.284
II + MeOH	3.420	5.158	336.112
III	5.696	−6.531	289.276
IV	1.174	−9.199	295.489
V	9.547	−3.287	287.239
VI	−7.449	−17.399	296.912

a *The calculation condition is at 298.15 K under 1 atm*.

From our DFT studies, some important experimental phenomena can be well-explained. First, the stability of the basic Et group on the Zn atom of the catalyst can be explained by noting that the acidic proton of the initiator (RO-H) was retained on the phenoxyl oxygen of catalyst by hydrogen bond and the crowded structures of the catalysts (**III**, **IV**, and **VI**). These protected the Et group on the Zn atom from the acid proton. Second, the relative activities of the four catalysts can also be rationalized, possibly by the size of the pocket for accepting LA on the catalysts found in intermediate **I**, besides the electronic effects on the zinc center. The size of the pocket decides the easiness of LA entering the catalyst center, and the smaller pocket size also makes monomer coordination slightly different between *D*-LA and *L*-LA to reveal the higher stereselectivity in *rac*-LA polymerization. The pocket sizes of the four catalysts according to the sizes of the pendant groups and the percent buried volume (%V_bur_) of the ligands with a sphere radius of 7 Å measured from the optimized **L'ZnEt** structures (Supporting Information) are in the following order: **L^4′^ZnEt** (32.6%) > **L^1′^ZnEt** (32.8%) > **L^3′^ZnEt** (33.4%) > **L^2′^ZnEt** (33.6%) (Falivene et al., 2016). This order is coincident with that of their catalytic activities and opposed to stereselectivity.

## Conclusion

In this study, a series of aminophenolate ligands with various pendant groups and associated ethyl Zn complexes were synthesized to investigate the effect of pendant groups on the catalytic activity of the complexes during LA polymerization. The thiophenylmethyl group (**L**^**4**^**ZnEt**) increased the catalytic activity more than the benzyl group (**L**^**1**^**ZnEt**) did, and 2-fluorobenzyl (**L**^**3**^**ZnEt**) and 2-methoxybenzyl (**L**^**2**^**ZnEt**) groups showed the opposite effect. The new LA polymerization mechanism was proven by NMR study and DFT calculation, which showed that LA was attracted by the H···O bond of an α-hydrogen of the LA molecule and the phenoxyl oxygen of the catalyst. After the dissociation of the amino group from the Zn atom, the benzyl alcohol initiated LA without replacing the ethyl group of the Zn complex. It is the first case that the ethyl group is regarded as a ligand and cannot be replaced by benzyl alcohol. This information provides researchers with another possible mechanisms of ROP by using alkyl zinc complexes as catalysts.

## Experiment Section

### General

Standard Schlenk techniques and a N_2_-filled glove box were used throughout the isolation and handling of all the compounds. Solvents, L-LA, and deuterated solvents were purified prior to use. N,N-dimethylethylenediamine, 2,4-bis(α,α-dimethylbenzyl)phenol, sodium borohydride, benzaldehyde, 2-methoxybenzaldehyde, 2-fluorobenzaldehyde, 2-thiophenecarboxaldehyde, and diethyl zinc were purchased from Aldrich. ^1^H and ^13^C NMR spectra were recorded on a Varian Unity Inova-600 (600 MHz for ^1^H and 150 MHz for ^13^C) or a Varian Mercury-400 (400 MHz for ^1^H and 100 MHz for ^13^C) spectrometer with chemical shifts given in ppm from the internal tetramethylsilane or the central line of CDCl_3_. Gel permeation chromatography (GPC) measurements were performed on a Jasco PU-2080 plus system equipped with a RI-2031 detector using THF (high-performance liquid chromatography grade) as an eluent (flow rate 1.0 mL/min, at 40°C). The chromatographic column was Phenomenex Phenogel 5 μm 103 Å, and the calibration curve used to calculate Mn(GPC) was produced from polystyrene standards. The GPC results were calculated using the Scientific Information Service Corporation (SISC) chromatography data solution 3.1 edition.

### Ligands and Associated Zn Complexes Synthesis

#### Synthesis of L^1^-H

*N*,*N*-Dimethylethylenediamine (0.88 g, 10 mmole) and benzaldehyde (1.06 g, 10 mmole) were refluxed in ethanol (20 mL) for 6 h. When the solution was cooled to 25°C, sodium borohydride (0.42 g, 11 mmole) was added and stirred overnight to form *N*^1^-benzyl-*N*^2^,*N*^2^-dimethylethane-1,2-diamine. After removing the precipitate from the solution, 2,4-bis(α,α-dimethylbenzyl)phenol (3.3 g, 10 mmole) and paraformaldehyde (0.45 g, 15 mmole) were transferred into the solution and refluxed for 12 h. Volatile materials were removed under vacuum to produce yellow mud. The mud was dissolved in CH_2_Cl_2_ (20 mL) and the solution was washed with water (3 × 40 mL), and the solvent removed at reduced pressure to produce the white powders. Yield: 3.08 g (59%). ^1^H NMR (CDCl_3_, 400 MHz): δ10.42 (1H, s, ArO*H*), 7.27 (5H, m, Ph*H*), 7.23 (4H, m, Ph*H*), 7.17 (5H, m, Ph*H*), 6.91 (1H, m, Ph*H*), 6.89 (1H, m, Ar*H*), 6.75 (1H, d, Ar*H*), 3.60 (2H, s, NC*H*_2_Ar), 3.37 (2H, s, NC*H*_2_Ph), 2.39 (2H, t, *J* = 4 Hz), NC*H*_2_CH_2_N), 2.27 (2H, t, *J* = 4 Hz, NCH_2_C*H*_2_N), 1.96 (6H, s, NC*H*_3_), 1.68 (12H, s, C(C*H*_3_)_2_Ph). ^13^C NMR (CDCl_3_, 100 MHz): δ153.46 (*ArC*-OH), 151.65, 151.46, 139.50, 137.52, 135.31, 129.69, 128.14, 127.80, 127.55, 127.07, 126.70, 126.37, 125.66, 125.30, 124.62, 124.54, 122.19 (*ArC* and *PhC*), 57.85 (N*C*Ph), 57.37 (*C*N(CH_3_)_2_), 55.82 (Ar*C*N), 49.84 (N*C*CN), 45.07 (CN(*C*H_3_)_2_), 42.40, 41.99 (Ar*C*(CH_3_)_2_Ph), 31.10, 29.44 (ArC(*C*H_3_)_2_Ph). Anal. Calc. (found) for C_36_H_44_N_2_O: C 82.76% (83.03%), H 8.35% (8.52%), N 5.36% (5.38%).

#### Synthesis of L^2^-H

We used a method similar to that for L^1^-H, except 2-methoxybenzaldehyde was used to replace benzaldehyde. Yield: 2.86 g (52%). ^1^H NMR (CDCl_3_, 400 MHz): δ10.22 (1H, s, ArO*H*), 7.24 (5H, m, Ph*H*), 7.20 (4H, m, Ph*H*), 7.16 (3H, m, Ph*H*), 6.88 (1H, m, Ar*H*), 6.78 (2H, m, Ph*H*), 6.71 (1H, d, Ar*H*), 3.64 (3H, s, PhOC*H*_3_), 3.59 (2H, s, NC*H*_2_Ar), 3.54 (2H, s, NC*H*_2_Ph), 2.41 (2H, t, *J* = 4 Hz, NC*H*_2_CH_2_N), 2.18 (2H, t, *J* = 4 Hz, NCH_2_C*H*_2_N), 2.01 (6H, s, NC*H*_3_), 1.67 (12H, s, C(C*H*_3_)_2_Ph). ^13^C NMR (CDCl_3_, 100 MHz): δ157.92 (*PhC*OCH_3_), 153.62 (*ArC*-OH), 151.64, 151.45, 139.42, 135.06, 131.63, 128.68, 127.76, 127.45, 126.66, 125.84, 125.65, 125.26, 125.23, 124.47, 124.36, 122.14, 120.26, 110.15 (*ArC* and *PhC*), 58.09 (N*C*Ph), 56.08 [*C*N(CH_3_)_2_], 54.96 (Ar*C*N), 53.05 (PhO*C*H_3_), 50.18 (N*C*CN), 45.23 [CN(*C*H_3_)_2_], 42.34, 41.86 [Ar*C*(CH_3_)_2_Ph], 31.04, 29.35 [ArC(*C*H_3_)_2_Ph]. Anal. Calc. (found) for C_37_H_46_N_2_O_2_: C 79.79% (80.69%), H 8.30% (8.42%), N 5.04% (5.09%).

#### Synthesis of L^3^-H

We used a method similar to that for L^1^-H, except 2-florobenzaldehyde was used to replace benzaldehyde. Yield: 3.39 g (63%). ^1^H NMR (CDCl_3_, 400 MHz): δ10.32 (1H, s, ArO*H*), 7.25 (5H, m, Ph*H*), 7.22 (4H, m, Ph*H*), 7.15 (3H, m, Ph*H*), 6.93 (1H, m, Ph*H*), 6.91 (1H, m, Ar*H*), 6.76 (1H, m, Ph*H*), 6.73 (1H, d, Ar*H*), 3.61 (2H, s, NC*H*_2_Ar), 3.47 (2H, s, NC*H*_2_Ph), 2.42 (2H, t, *J* = 4 Hz, NC*H*_2_CH_2_N), 2.28 (2H, t, *J* = 4 Hz, NCH_2_C*H*_2_N), 1.98 (6H, s, NC*H*_3_), 1.68 [12H, s, C(C*H*_3_)_2_Ph]. ^13^C NMR (CDCl_3_, 100 MHz): δ162.51 (*PhC*F), 160.07 (*ArC*-OH), 153.48, 151.64, 151.42, 139.56, 135.30, 132.36, 132.32, 128.85, 128.77, 127.80, 127.52, 126.69, 126.34, 125.66, 125.31, 124.67, 124.54, 124.15, 124.11, 124.03, 122.03 (*ArC* and *PhC*), 57.31 (N*C*Ph), 55.84 [*C*N(CH_3_)_2_], 49.89 (Ar*C*N), 43.59 (N*C*CN), 45.06 [CN(*C*H_3_)_2_], 42.39, 41.99 [Ar*C*(CH_3_)_2_Ph], 31.07, 29.42 [ArC(*C*H_3_)_2_Ph]. Anal. Calc. (found) for C_36_H_43_N_2_OF: C 80.12% (80.26%), H 8.07% (8.04%), N 5.40% (5.20%).

#### Synthesis of L^4^-H

We used a method similar to that for L^1^-H, except 2-thiophenecarboxaldehyde was used to replace benzaldehyde.Yield: 3.16 g (60%). ^1^H NMR (CDCl_3_, 400 MHz): δ10.15 (1H, s, ArO*H*), 7.27 (4H, d, Ph*H*), 7.22 (4H, d, Ph*H*), 7.15 (2H, m, Ph*H*), 7.10 (1H, m, Ph*H*), 6.87 (1H, t, CHC*H*CHS), 6.72 (1H, d, CHCHC*H*S), 6.63 (1H, d, C*H*CHCHS), 3.69 (2H, s, NC*H*_2_Ar), 3.59 (2H, s, NC*H*_2_Ph), 2.51 (2H, t, *J* = 4 Hz, NC*H*_2_CH_2_N), 2.32 (2H, t, *J* = 4 Hz, NCH_2_C*H*_2_N), 2.05 (6H, s, NC*H*_3_), 1.68 [12H, s, C(C*H*_3_)_2_Ph]. ^13^C NMR (CDCl_3_, 100 MHz): δ153.60 (*ArC*-OH), 151.53, 151.43, 139.68, 138.53, 135.23, 127.81, 127.56, 126.70, 126.64, 126.48, 125.64, 125.31, 125.14, 124.69, 124.61, 121.76 (*ArC, PhC*, and *thioC*), 56.70 (N*C*Ph), 56.27 [*C*N(CH_3_)_2_], 50.88 (Ar*C*N), 49.71 (N*C*CN), 45.19 [CN(*C*H_3_)_2_], 42.38, 42.05 [Ar*C*(CH_3_)_2_Ph], 31.03, 29.48 [ArC(*C*H_3_)_2_Ph]. Anal. Calc. (found) for C_34_H_42_N_2_OS: C 77.08% (77.52%), H 7.69% (8.04%), N 5.11% (5.32%).

#### Synthesis of L^1^ZnEt

ZnEt_2_ (0.617 g, 5.0 mmol) was added slowly to an ice cold (0°C) solution of L^1^-H (2.6 g, 5.0 mmol) in THF (15 mL), and the solution was stirred for 3 h. Volatile materials were removed under vacuum to yield a yellow mud. The mud was washed with hexane (20 mL) and a white powder was obtained after filtration. Yield: 2.54 g (83%). ^1^H NMR (CDCl_3_, 400 MHz): δ7.48 (2H, d, Ph*H*), 7.41 (3H, t, Ph*H*), 7.27 (7H, m, Ph*H*), 7.18 (1H, m, Ph*H*), 7.10 (1H, t, Ph*H*), 4.01, 3.93 (2H, d, *J* = 6 Hz, NC*H*_2_Ph), 3.92, 3.23 (2H, d, *J* = 6 Hz, NC*H*_2_Ph), 2.67 (1H, m, NC*H*_2_CH_2_N), 2.45 (1H, m, NC*H*_2_CH_2_N), 2.33 (1H, m, NC*H*_2_CH_2_N), 2.16 (1H, m, NCH_2_C*H*_2_N), 2.11 (3H, s, NC*H*_3_), 1.98 [3H, s, C(C*H*_3_)_2_Ph], 1.71 [3H, s, C(C*H*_3_)_2_Ph], 1.68 [6H, s, C(C*H*_3_)_2_Ph], 1.33 (3H, t, ZnCH_2_C*H*_3_), 1.28 (3H, s, NC*H*_3_), 0.12 (2H, m, ZnC*H*_2_CH_3_). ^13^C NMR (CDCl_3_, 100 MHz): δ164.87 (*ArC*-OH), 152.52, 151.02, 136.60, 133.63, 132.30, 131.75, 131.59, 128.41, 128.41, 128.36, 128.15, 127.82, 127.69, 127.56, 127.28, 127.13, 126.98, 126.72, 125.88, 125.71, 124.98, 124.18, 124.84 (*ArC* and *PhC*), 59.96 (N*C*Ph), 58.44 [*C*N(CH_3_)_2_], 57.25 (Ar*C*N), 46.85 (N*C*CN), 45.15 [CN(*C*H_3_)_2_], 44.01, 42.14 [Ar*C*(CH_3_)_2_Ph], 31.02, 26.78 [ArC(*C*H_3_)_2_Ph], 13.21 (ZnCH_2_*C*H_3_),−3.17 (Zn*C*H_2_CH_3_). Anal. Calc. (found) for C_38_H_48_N_2_OZn: C 74.66% (74.31%), H 7.97% (7.88%), N 4.86% (4.56%).

#### Synthesis of L^2^ZnEt

We used a method similar to that for **L**^**2**^**ZnEt**, except L^2^-H was used to replace L^1^-H. L^2^ZnEt followed the procedure of L^2^ZnEt. Yield: 2.51g (78%). ^1^H NMR (CDCl_3_, 400 MHz): δ7.45 (2H, d, Ph*H*), 7.33 (2H, m, Ph*H*), 7.19 (7H, m, Ph*H*), 7.07 (2H, m, Ph*H*), 6.93 (2H, m, Ph*H*), 6.51 (1H, s, Ph*H*), 4.14, 4.06 (2H, d, *J* = 6 Hz, NC*H*_2_Ph), 3.91, 3.12 (2H, d, *J* = 6 Hz, NC*H*_2_Ph), 3.76 (3H, s, PhOC*H*_3_), 2.50 (2H, m, NC*H*_2_CH_2_N), 2.34 (2H, m, NC*H*_2_CH_2_N), 2.01 (3H, s, NC*H*_3_), 1.95 [3H, s, C(C*H*_3_)_2_Ph], 1.64 [3H, s, C(C*H*_3_)_2_Ph], 1.61 [6H, s, C(C*H*_3_)_2_Ph], 1.32 (3H, t, ZnCH_2_C*H*_3_), 1.06 (3H, s, NC*H*_3_), 0.09 (2H, m, ZnC*H*_2_CH_3_). ^13^C NMR (CDCl_3_, 100 MHz): δ165.03 (*PhC*OCH_3_), 164.14 (*ArC*-OH), 158.42, 152.59, 152.10, 150.76, 150.31, 133.85, 129.91, 128.23, 127.61, 127.48, 127.44, 126.89, 126.63, 125.69, 124.89, 124.12, 120.28, 111.00, 110.78 (*ArC* and *PhC*), 67.91 (PhO*C*H_3_), 58.91 (N*C*Ph), 57.52 [*C*N(CH_3_)_2_], 55.29 (Ar*C*N), 52.47 (N*C*CN), 45.34 [CN(*C*H_3_)_2_], 42.08, 31.10 [Ar*C*(CH_3_)_2_Ph], 29.39, 26.21 [ArC(*C*H_3_)_2_Ph], (ZnCH_2_*C*H_3_) 13.21,−3.63 (Zn*C*H_2_CH_3_). Anal. Calc. (found) for C_39_H_50_N_2_O_2_Zn: C 72.27% (72.71%), H 7.28% (7.82%), N 4.52% (4.35%).

#### Synthesis of L^3^ZnEt

We used a method similar to that for **L**^**3**^**ZnEt**, except L^3^-H was used to replace L^1^-H.Yield: 2.58 g (82%). ^1^H NMR (CDCl_3_, 400 MHz): δ7.42 (2H, d, Ph*H*), 7.33 (2H, m, Ph*H*), 7.18 (7H, m, Ph*H*), 7.11 (2H, m, Ph*H*), 7.06 (2H, m, Ph*H*), 6.48 (1H, s, Ph*H*), 4.08, 3.94 (2H, d, *J* = 6 Hz NC*H*_2_Ph), 3.91, 3.14 (2H, d, *J* = 6 Hz, NC*H*_2_Ph), 2.47-2.44 (2H, m, NCH_2_C*H*_2_N), 2.08 (3H, s, NC*H*_3_), 2.00-1.94 (2H, m, NC*H*_2_CH_2_N), 1.92 [3H, s, C(C*H*_3_)_2_Ph], 1.63 [3H, s, C(C*H*_3_)_2_Ph], 1.61 [6H, s, C(C*H*_3_)_2_Ph], 1.28 (3H, t, ZnCH_2_C*H*_3_), 1.10 (3H, s, NC*H*_3_), 0.05 (2H, m, ZnC*H*_2_CH_3_). ^13^C NMR (CDCl_3_, 100 MHz): δ164.91 (*PhC*F), 163.01 (*ArC*-OH), 160.57, 152.55, 150.87, 136.62, 134.07, 133.66, 130.53, 128.23, 127.56, 127.39, 126.96, 126.70, 125.86, 124.98, 124.19, 121.63, 119.53, 119.37, 115.87, 115.64 (*ArC* and *PhC*), 58.36 (N*C*Ph), 57.24 [*C*N(CH_3_)_2_], 51.92 (Ar*C*N), 46.11 (N*C*CN), 45.15 [CN(*C*H_3_)_2_], 42.15, 41.96 [Ar*C*(CH_3_)_2_Ph], 31.04, 26.62 [ArC(*C*H_3_)_2_Ph], (ZnCH_2_*C*H_3_) 13.18,−3.68 (Zn*C*H_2_CH_3_). Anal. Calc. (found) for C_38_H_47_N_2_OFZn: C 71.96% (72.20%), H 7.49% (7.49%), N 4.52% (4.43%).

#### Synthesis of L^4^ZnEt

We used a method similar to that for **L**^**4**^**ZnEt**, except L^4^-H was used to replace L^1^-H. Yield: 2.29 g (74%). ^1^H NMR (CDCl_3_, 400 MHz): δ 7.40 (2H, d, *J* = 4 Hz, Ph*H*), 7.29 (1H, d, *J* = 2 Hz, thio-*H*), 7.21-7.10 (7H, m, Ph*H*), 7.05-6.99 (1H, m, Ph*H*), 6.88 (1H, s, Ph*H*), 6.56 (1H, s, Ph*H*), 4.18, 3.98 (2H, d, *J* = 6 Hz, NC*H*_2_Ph), 3.73, 3.31 (1H, d, *J* = 6 Hz, NC*H*_2_Ph), 2.66, 2.46 (2H, m, NC*H*_2_CH_2_N), 2.30, 2.23 (2H, m, NCH_2_C*H*_2_N), 2.11 (3H, s, NC*H*_3_), 1.89 [3H, s, C(C*H*_3_)_2_Ph], 1.65 [3H, s, C(C*H*_3_)_2_Ph], 1.62 [6H, s, C(C*H*_3_)_2_Ph], 1.35 (3H, s, NC*H*_3_), 1.22 (3H, t, ZnCH_2_C*H*_3_), 0.01 (2H, m, ZnC*H*_2_CH_3_). ^13^C NMR (CDCl_3_, 100 MHz): δ164.78 (*ArC*-OH), 152.49, 151.11, 136.79, 133.75, 133.65, 130.30, 128.10, 127.58, 127.21, 127.11,126.98, 126.73, 126.58, 125.97, 124.98, 124.17, 121.85 (*ArC, PhC* and *thioC*), 57.26 (N*C*Ph), 57.10 [*C*N(CH_3_)_2_], 53.07 (Ar*C*N), 47.49 (N*C*CN), 46.84 [CN(*C*H_3_)_2_], 44.28, 42.06 [Ar*C*(CH_3_)_2_Ph], 30.99, 26.98 [ArC(*C*H_3_)_2_Ph], (ZnCH_2_*C*H_3_) 13.18,−3.84 (Zn*C*H_2_CH_3_). Anal. Calc. (found) for C_36_H_46_N_2_OSZn: C 69.65% (69.74%), H 7.43% (7.48%), N 4.68% (4.52%).

### General Procedures for the Polymerization of L-LA and rac-LA

A typical polymerization procedure was exemplified by the synthesis of entry 1 of Table 1. The mixture of *L*-lactide (0.72 g, 5 mmol) and BnOH (5 mmol) in 5 mL toluene was added in the solution of zinc complex in toluene (5 mL) at 25°C. After the solution was stirred for 6.5 h, the reaction was then quenched by adding to a drop of ethanol, and the polymer was precipitated pouring into *n*-hexane (20.0 mL) to give white solids. The white solid was dissolved in CH_2_Cl_2_ (2.0 mL) and then *n*-hexane (20.0 mL) was added to give white crystalline solid. For *rac*-PLA, the *P*r values for the selectivity of the heterotactic PLA were identified through ^1^H NMR spectra (5.0−5.2 ppm) of *rac*-PLA after decoupling at 1.57 ppm (methine group of rac-PLA) as shown in Figure S21.

### X-Ray Crystallographic Study

X-ray diffraction data for a suitable crystal of L^1^ZnEt was collected on an Oxford diffraction limited Gemini S with graphite-monochromated Mo-Ka (λ = 0.71073 Å) radiation. All data were collected at 150 K with the ω-scan techniques. The structure was solved by direct methods and refined using Fourier techniques. An absorption correction based on SADABS was applied. All non-hydrogen atoms were refined by full-matrix least squares on F^2^ using the SHELXTL program package. Hydrogen atoms were located and refined by the geometry method. The cell refinement, data collection and reduction were done through the use of CrysAlisPro, Agilent Technologies, Version 1.171.37.31. The structure solution and refinement were performed by using SHELXS-97 and SHELXL-97, respectively. Molecular structure was generated by using the SHELXTL program.

### DFT Calculation

The DFT calculation was carried out using the Gaussian09 program. The structures were built according to the X-ray structure of **L**^**1**^**ZnEt**. Their geometry optimizations were carried out using B3LPY (Becke, 1993). The LanL2DZ basis set (Hay and Wadt, 1985a,b) was used for Zn atom, and the 6-31G(d) basis set (Ditchfield et al., 1971) was used for other atoms. The minimum energy stationary point was confirmed by frequency analysis with the same calculation level.

## Author Contributions

Theoretical calculation was performed by K-HW and the rest of work was performed by W-YL under the supervision of C-CL and H-YC.

### Conflict of Interest Statement

The authors declare that the research was conducted in the absence of any commercial or financial relationships that could be construed as a potential conflict of interest.
