# Supplementary material for: Synthesis and Characterization of N,N,O-Tridentate Aminophenolate Zinc Complexes and Their Catalysis in the Ring-Opening Polymerization of Lactides
Source: Front Chem. 2019 Apr 5;7:189. doi: 10.3389/fchem.2019.00189 (PMC6459900; doi:10.3389/fchem.2019.00189)
Supplement: Supplementary file 1 [file Data_Sheet_1.PDF]

Synthesis and Characterization of *N,N,O*-tridentate aminophenolate Zinc Complexes and Their Catalysis in the Ring-Opening Polymerization of Lactides

Wei-Yi Lu,[a] Kuo-Hui Wu,[b]* Hsuan-Ying Chen,[cd]* and Chu-Chieh Lin[a]*

a Department of Chemistry, National Chung Hsing University, Taichung 402, Taiwan, R.O.C.

b Department of Chemistry, Graduate School of Science, The University of Tokyo, Tokyo 113-0033, Japan.

c Department of Medicinal and Applied Chemistry, Kaohsiung Medical University, Kaohsiung 80708, Taiwan, R.O.C.

d Department of Medical Research, Kaohsiung Medical University Hospital, Kaohsiung 80708, Taiwan, R.O.C.

Electronic Supplementary Information Available: Polymer characterization data, and details of the kinetic study.

Table of Contents

Table S1 The variations of [LA] in ROP process with a wide range of [$\mathbf{L^3ZnEt}$] in toluene 10 mL, [*L*-LA] = 0.5 M, [BnOH] = 10 mM at room temperature. 2
Table S2 The variations of [LA] in ROP process with a wide range of [BnOH] in toluene 10 mL, [*L*-LA] = 0.5 M, [BnOH] = 10 mM at room temperature......................3
Table S3 Atomic coordinates of intermediate I……………………………….....…11
Table S4 Thermochemical data of intermediate I……………………………………15
Table S5 Atomic coordinates of intermediate II……………………………………..16
Table S6 Thermochemical data of intermediate II…………………………………...19
Table S7 Atomic coordinates of intermediate III…………………………………….20
Table S8 Thermochemical data of intermediate III……………………………….…23
Table S9 Atomic coordinates of intermediate IV…………………………………....24
Table S10 Thermochemical data of intermediate IV………………………………...27
Table S11 Atomic coordinates of intermediate V…………………………………....28
Table S12 Thermochemical data of intermediate V…………………………………31
Table S13 Atomic coordinates of intermediate VI…………………………………..32

Table S14 Thermochemical data of intermediate VI…………………………...…34
Table S15 Atomic coordinates of methanol……………………………………..…35
Table S16 Thermochemical data of methanol……………………………….…….36
Table S17 Atomic coordinates of L1'ZnEt…………………………………………37
Table S18 Atomic coordinates of L2'ZnEt…………………………………………40
Table S19 Atomic coordinates of L3'ZnEt…………………………………………43
Table S20 Atomic coordinates of L4'ZnEt…………………………………………47
Table S21 Crystal data and structure refinement for **L1ZnEt**……………………50-65
Figure S1-8 1H and 13C NMR spectrum of ligands and associated Zn complexes ..4-11
Figure S9 Chemical structure and ball and stick model of intermediate I…………..11
Figure S10 Chemical structure and ball and stick model of intermediate II………...15
Figure S11 Chemical structure and ball and stick model of intermediate III……..…20
Figure S12 Chemical structure and ball and stick model of intermediate IV……..…24
Figure S13 Chemical structure and ball and stick model of intermediate V………...28
Figure S14 Chemical structure and ball and stick model of intermediate VI………..32
Figure S15 Chemical structure and ball and stick model of L1'ZnEt………………....37
Figure S16 Chemical structure and ball and stick model of L2'ZnEt………………....40
Figure S17 Chemical structure and ball and stick model of L3'ZnEt………………....43
Figure S18 Chemical structure and ball and stick model of L4'ZnEt………………....47
Figure S19 1H NMR spectra of the reaction of **L4ZnEt** with one equivalent BnOH in *d*8-toluene.
Figure S20 1H NMR spectra of the LA polymerization ([LA]:[Zn]:[BnOH]=4:1:1, [LA]=0.02 M in *d*8-toluene (0.5 mL) at 25 °C)
Figure S21. 1H NMR spectra of rac-PLA (entry 9, Table 1) after decoupling at 1.57 ppm.

Table S1 The variations of [LA] in ROP process with a wide range of [**L3ZnEt**] in toluene 10 mL, [*L*-LA] = 0.5 M, [BnOH] = 10 mM at room temperature.

**LA : Zn : BnOH**	50:1:1	50:2:1	50:3:1	50:4:1
Time (h)	Conversion of PLA (%)[a]			
0.083333				8.08
0.166667	3.96		8.93	14.92
0.333333	6.59	8.9	13.65	20.83
0.5	12.61	13.49	18.41	26.38
0.666667	16.25	17.69	28.18	35.80
0.833333	19.22	22.55	34.80	43.99
1	22.49	26.07	39.57	51.78
1.166667	24.79	31.02	46.58	

1.333333	27.08	35.89		
1.5	28.76	41.58		
1.666667	32.62			
1.833333	36.43			
2	38.09			
2.333333	42.35			
k_{obs}	0.2346 (52)	0.3656 (141)	0.5439 (287)	0.6763 (397)
R^2	0.994	0.996	0.993	0.992

[a] Obtained from ^{1}H NMR analysis.

Table S2 The variations of [LA] in ROP process with a wide range of [BnOH] in toluene 10 mL, [*L*-LA] = 0.5 M, [BnOH] = 10 mM at room temperature.

**LA : Zn : BnOH**	50:1:1	50:1:2	50:1:3	50:1:4
Time (h)	Conversion of PLA (%)[a]			
0.033333				8.53
0.083333				13.36
0.166667	3.96	4.01	13.63	19.12
0.333333	6.59	9.45	18.32	23.14
0.5	12.61	13.99	22.89	35.06
0.666667	16.25	18.60	28.31	39.84
0.833333	19.22	21.69	34.33	43.63
1	22.49	25.84	38.19	48.79
1.166667	24.79	31.08	43.94	55.52
1.333333	27.08	35.32		60.64
1.5	28.76	41.51		64.69
1.666667	32.62	43.56		68.39
1.833333	36.43			72.64
2	38.09			75.17
2.333333	42.35			
k_{obs}	0.2346 (52)	0.3570 (125)	0.4317 (161)	0.6506 (118)
R^2	0.994	0.995	0.997	0.998

[a] Obtained from ^{1}H NMR analysis.

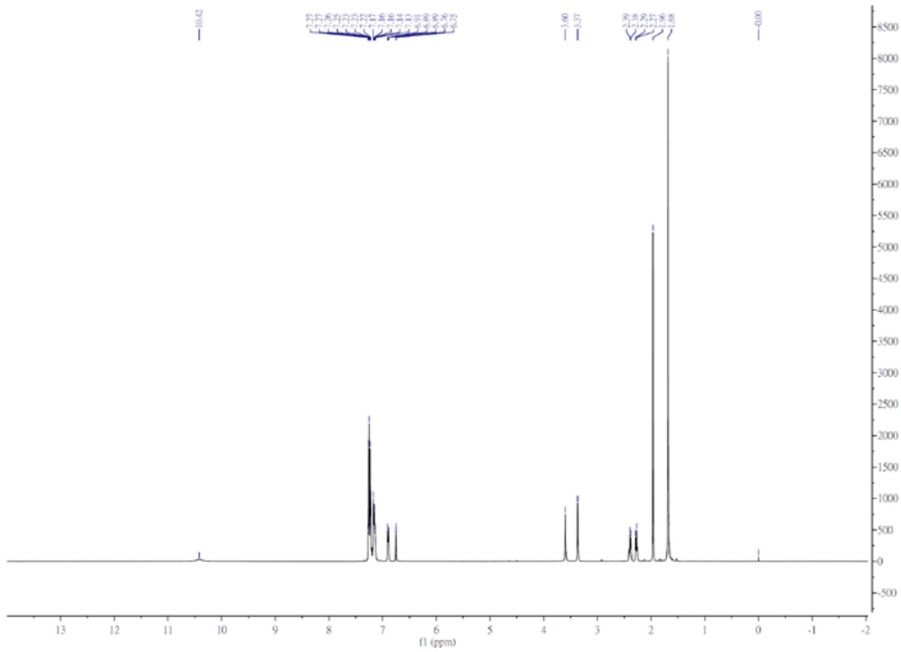

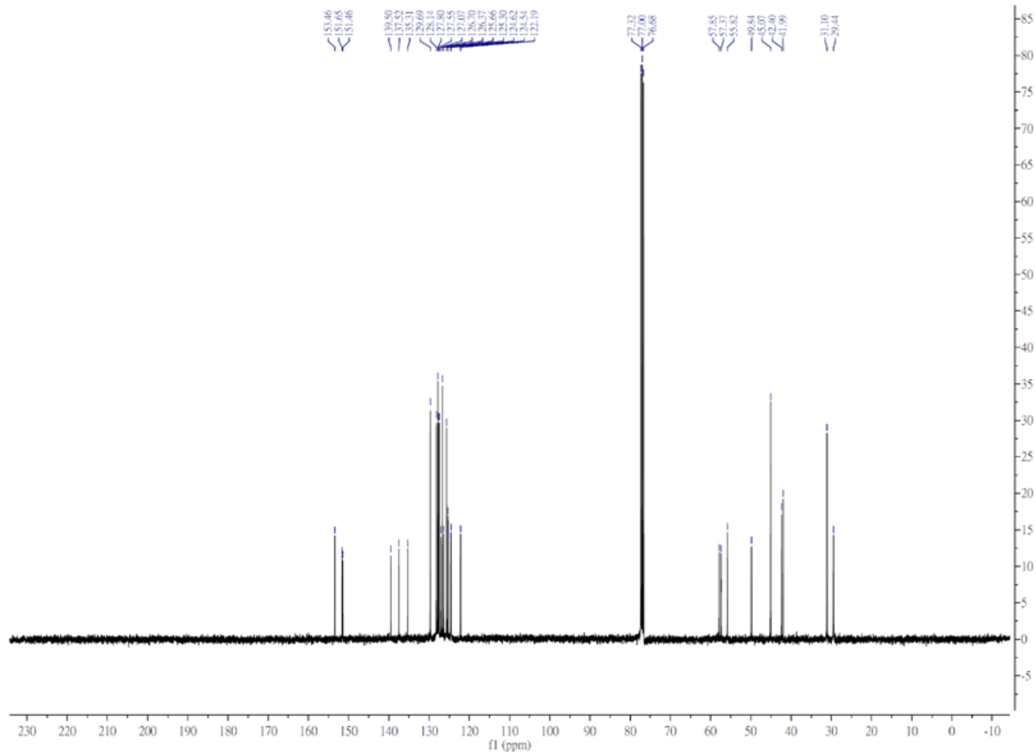

Figure S1 ^{1}H and ^{13}C NMR spectrum of L^{1}-H

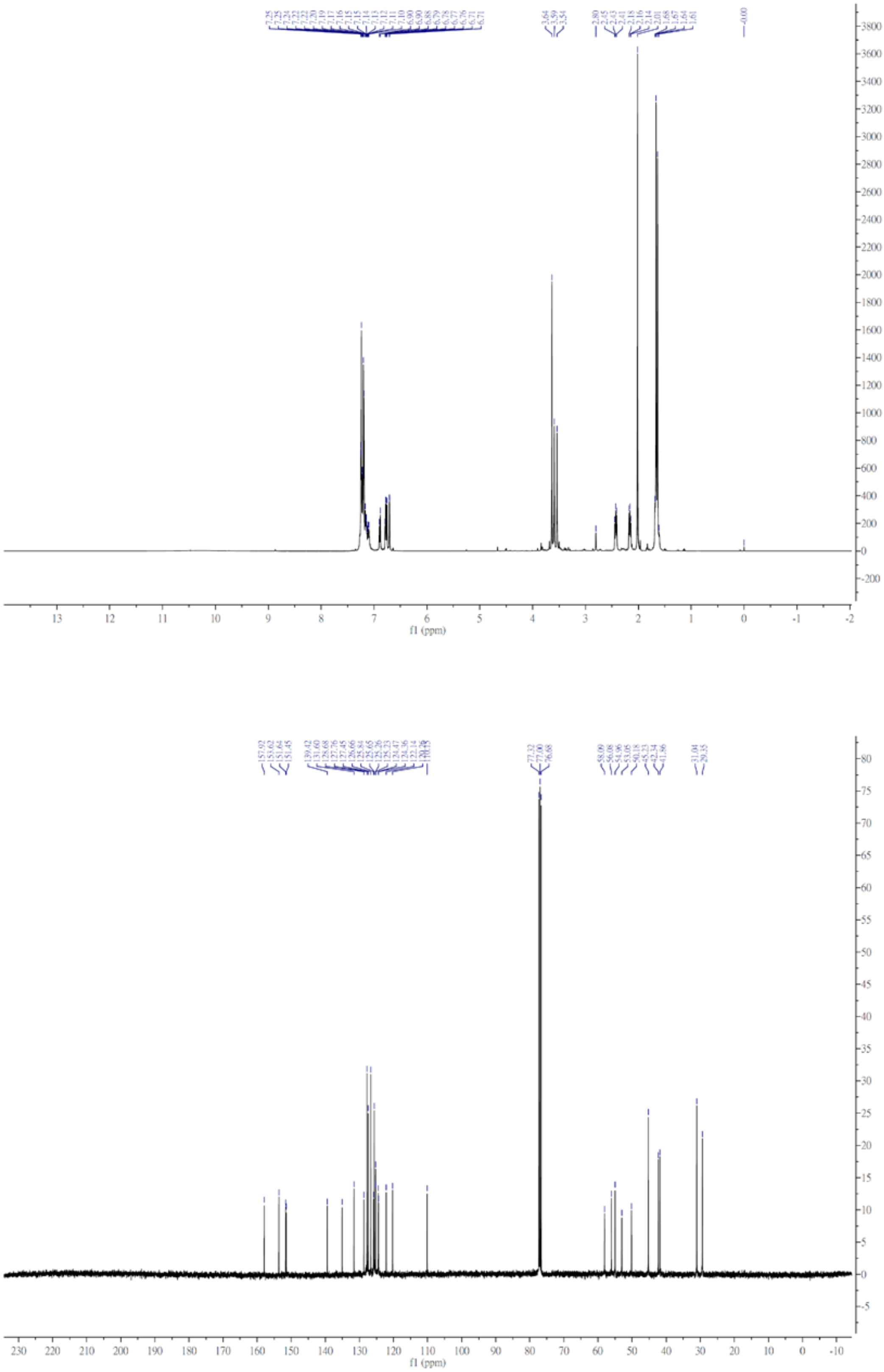

Figure S2 ^{1}H and ^{13}C NMR spectrum of L^{2}-H

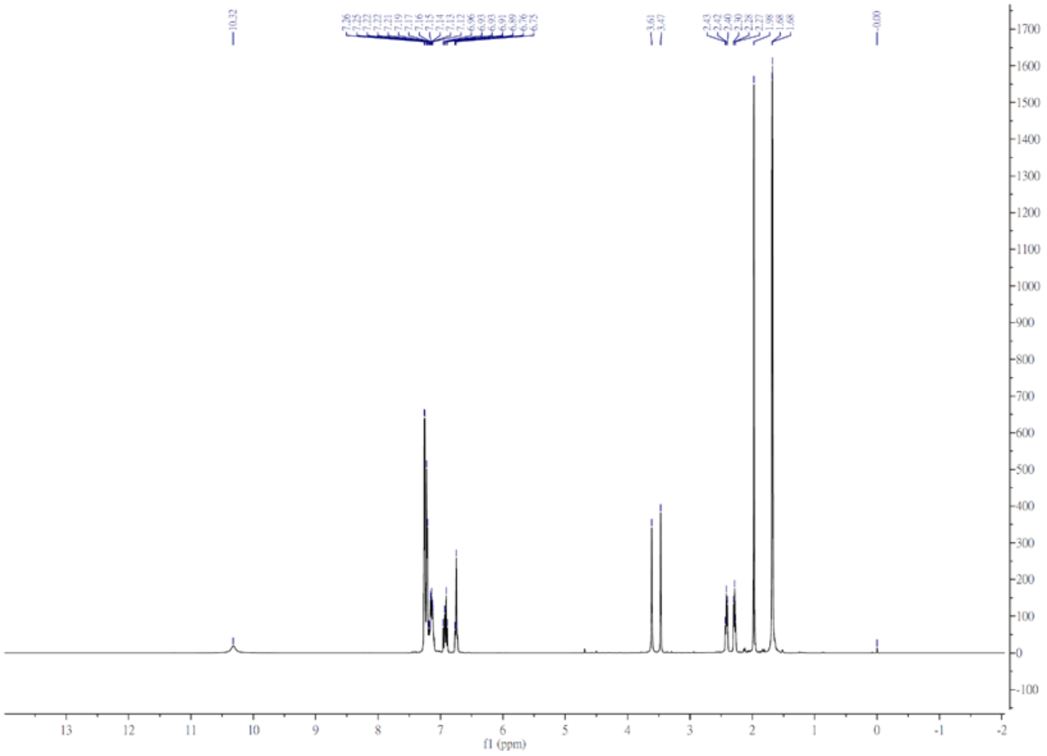

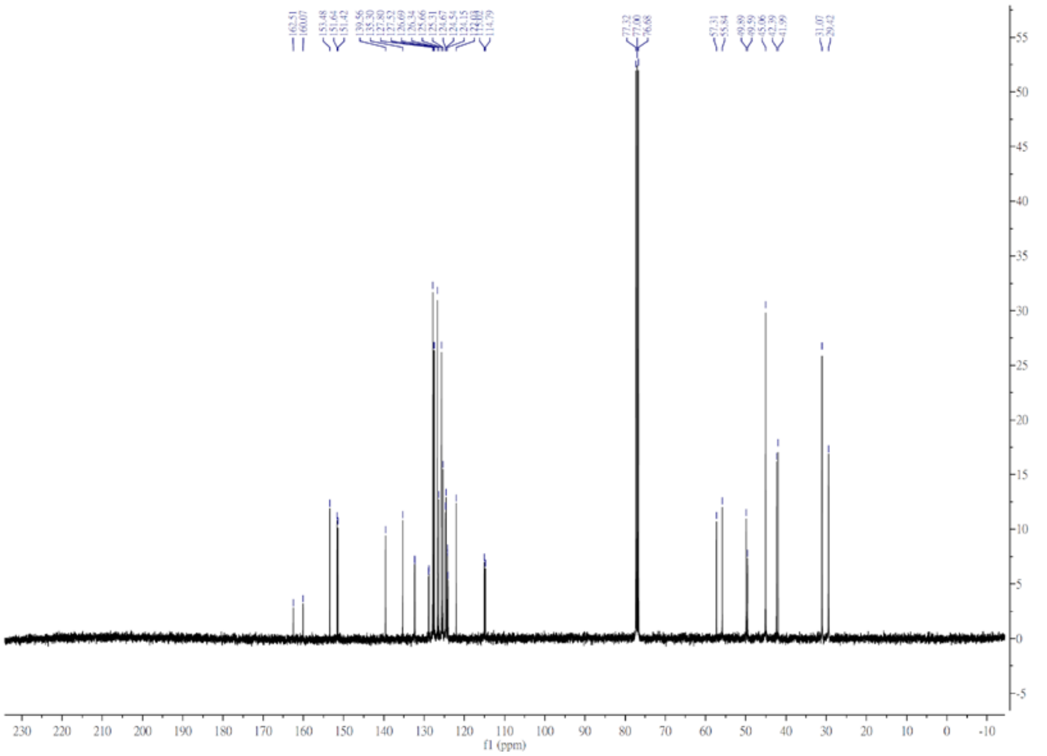

Figure S3 ^{1}H and ^{13}C NMR spectrum of L^{3}-H

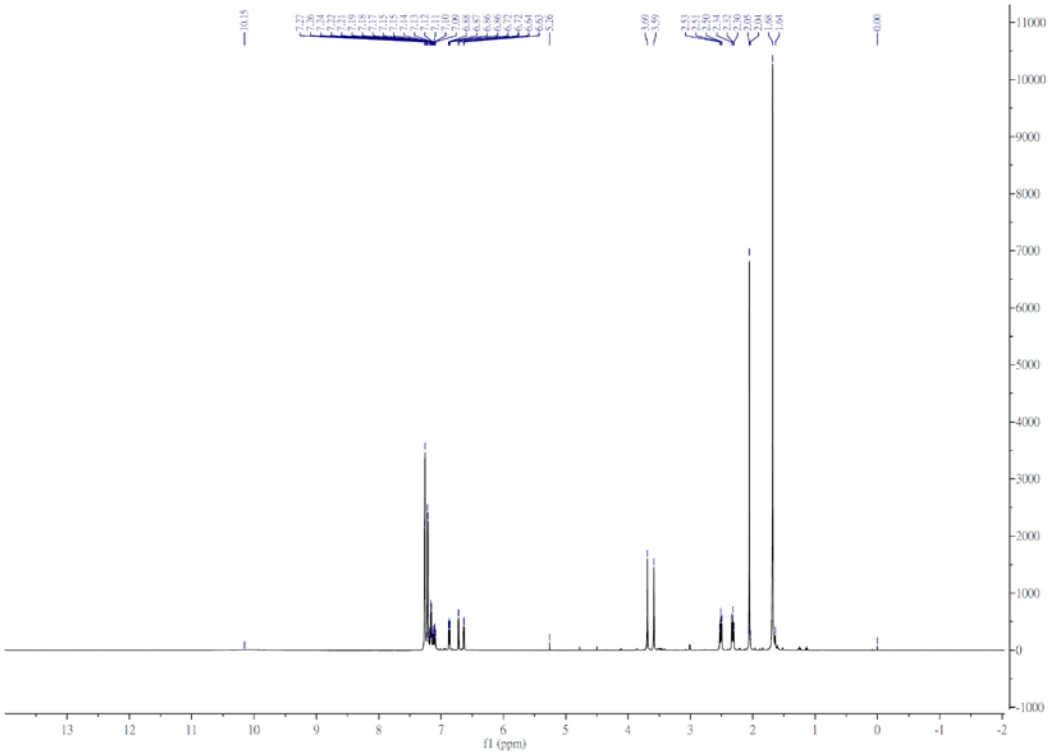

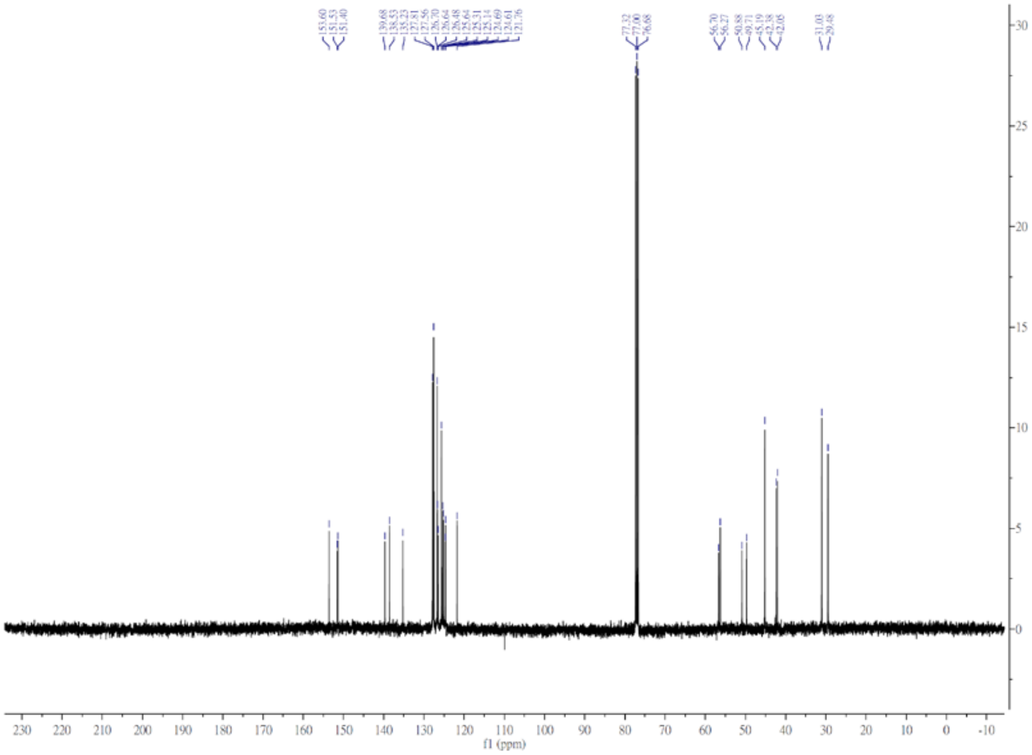

Figure S4 ^{1}H and ^{13}C NMR spectrum of L^{4}-H

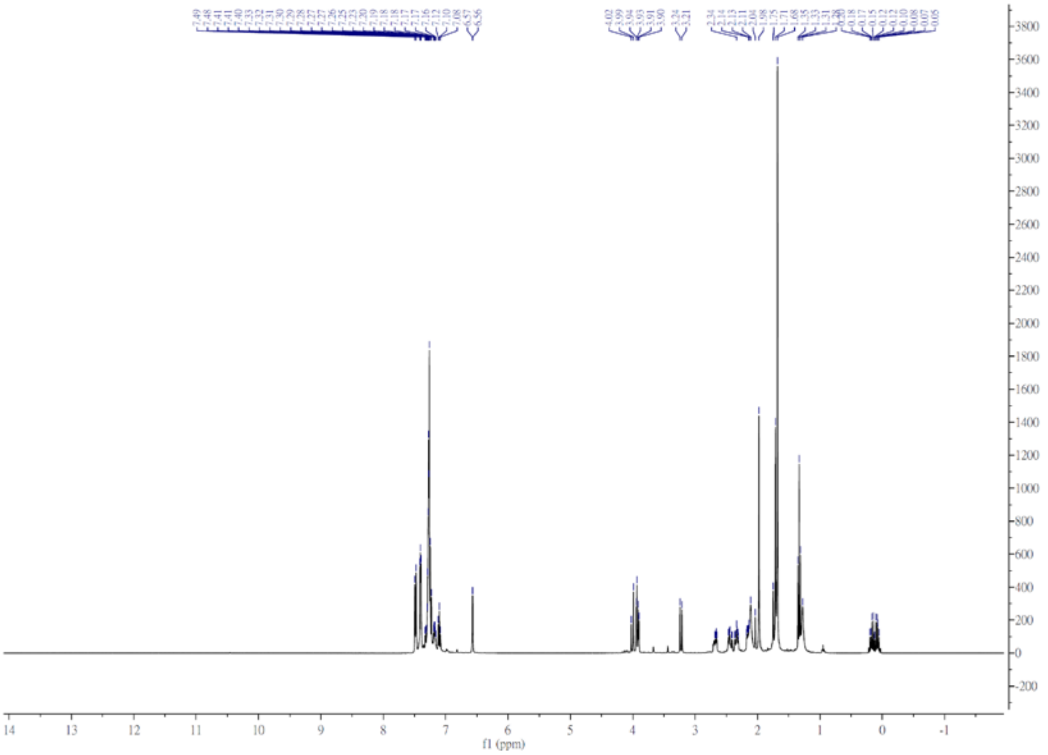

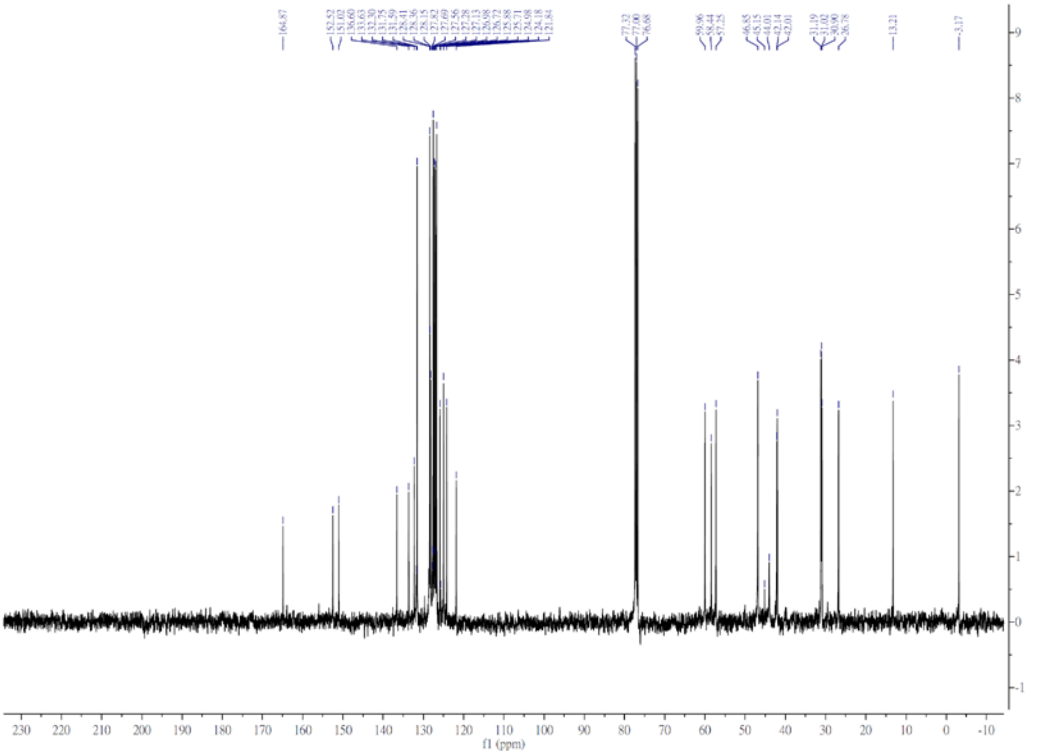

Figure S5 ^{1}H and ^{13}C NMR spectrum of $L^{1}ZnEt$

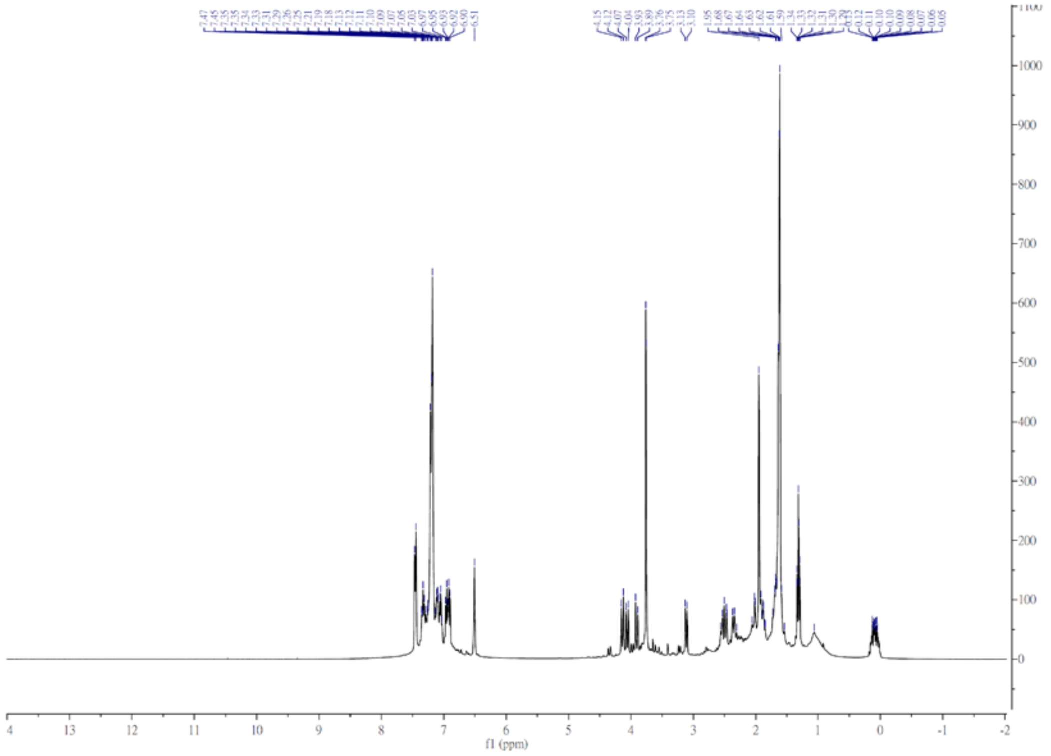

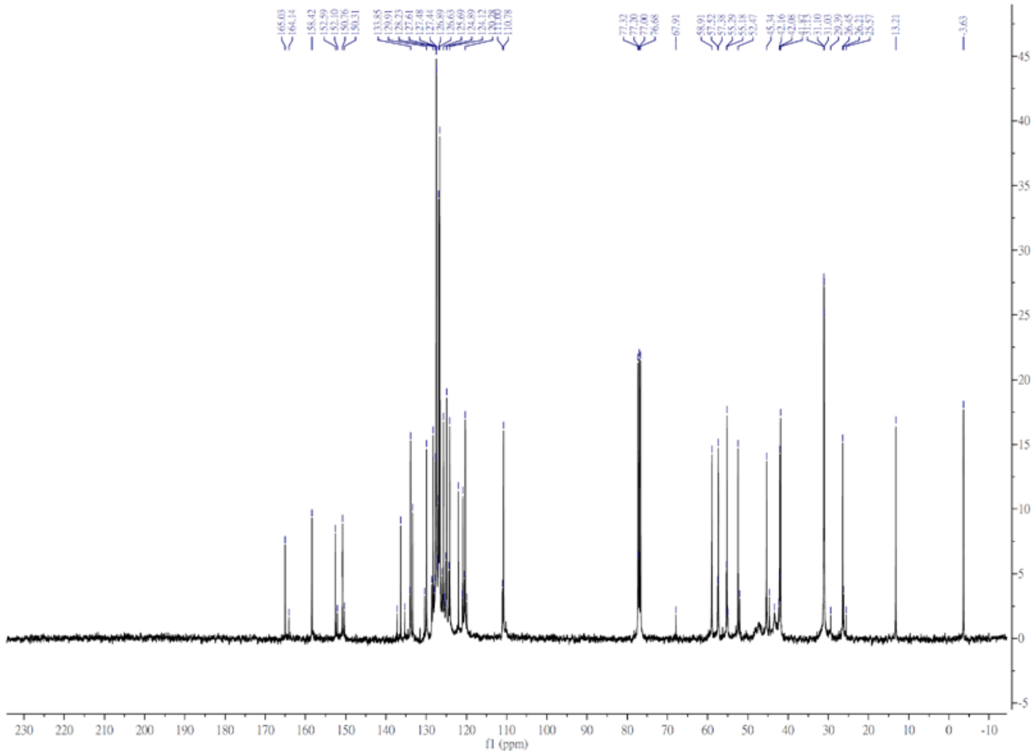

Figure S6 ^{1}H and ^{13}C NMR spectrum of L^{2}ZnEt

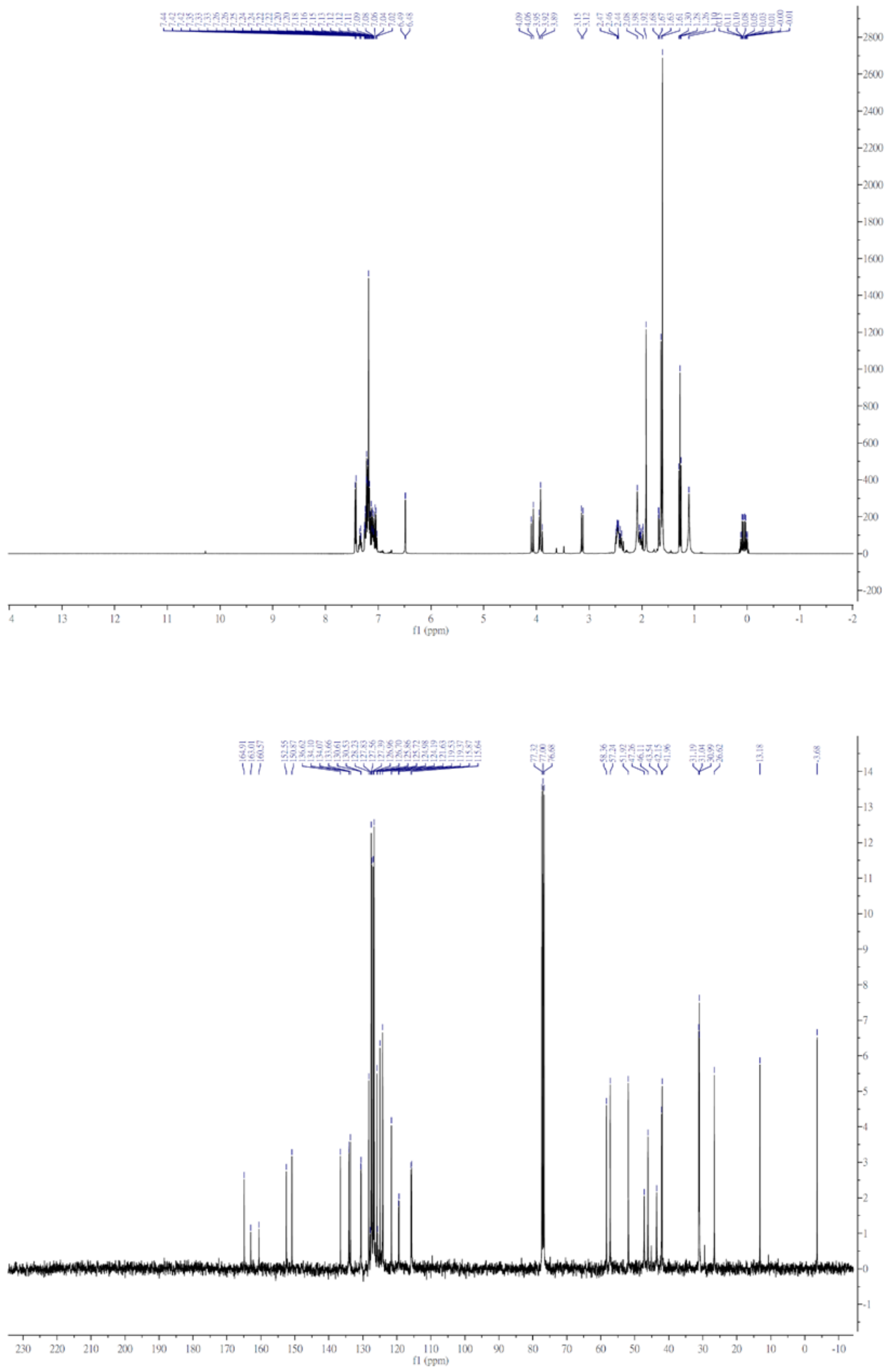

Figure S7 ^{1}H and ^{13}C NMR spectrum of $L^{3}ZnEt$

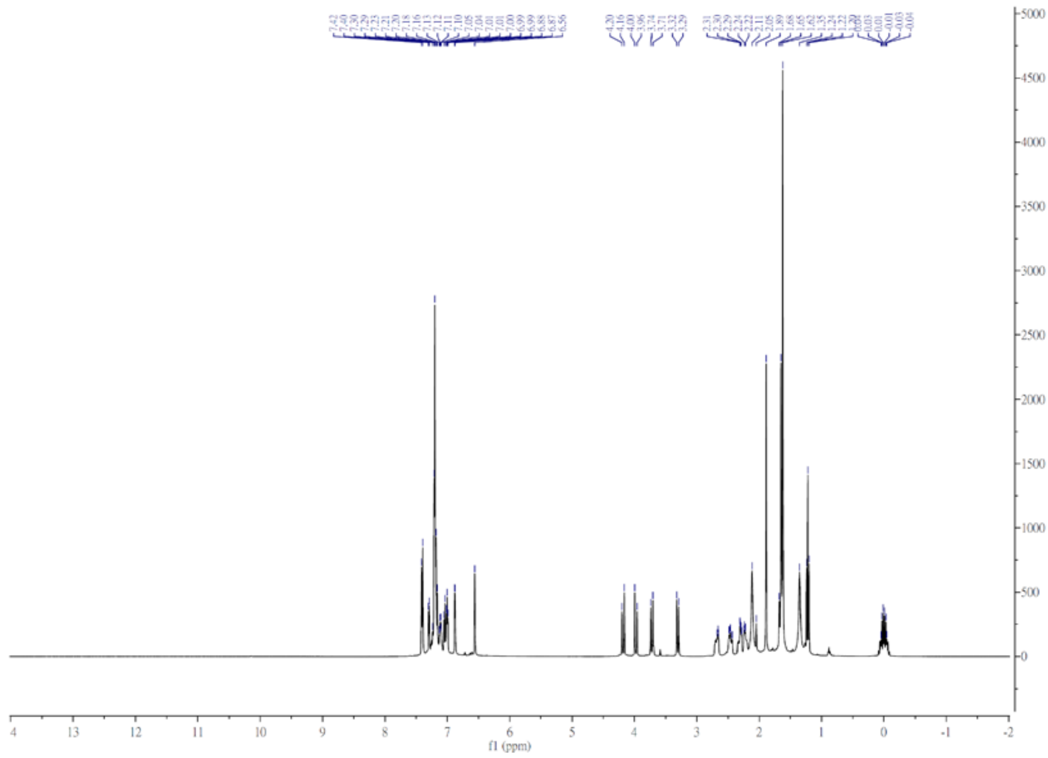

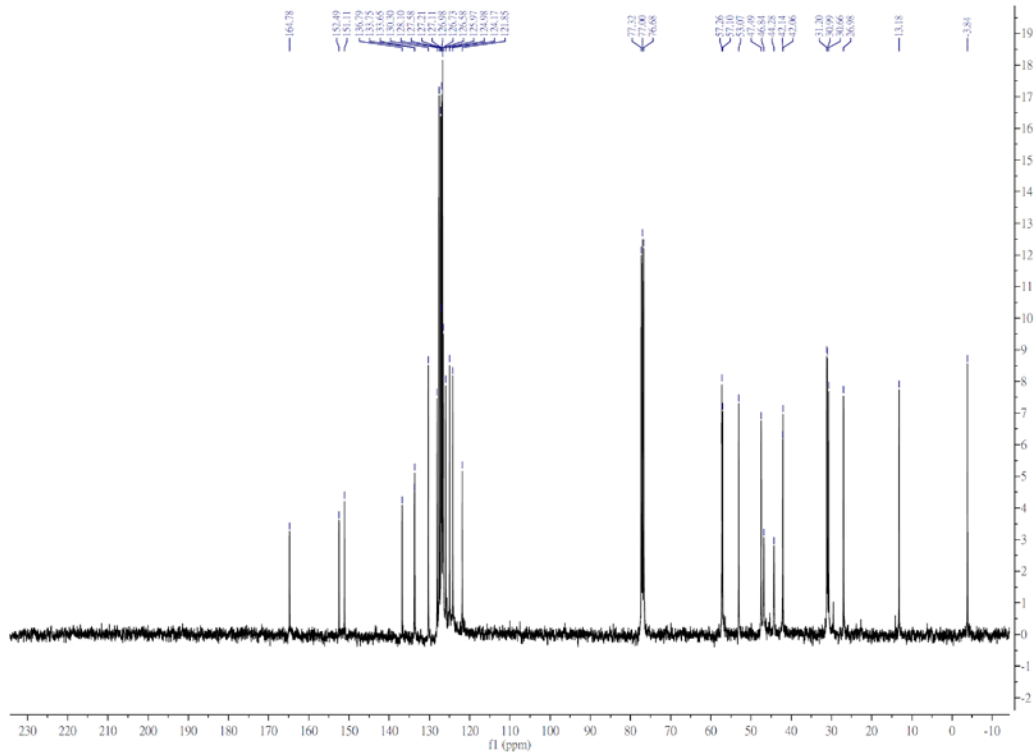

Figure S8 ^{1}H and ^{13}C NMR spectrum of $L^{4}ZnEt$

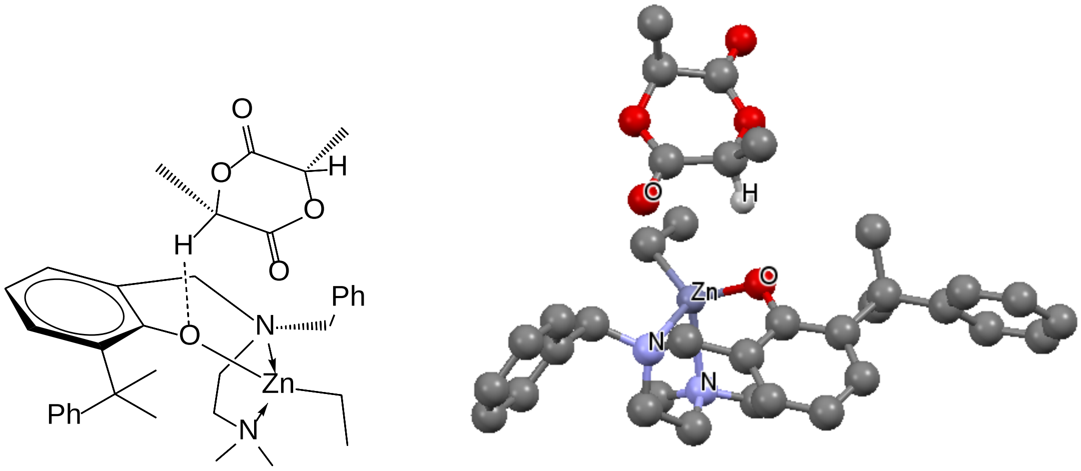

Figure S9 Chemical structure and ball and stick model of intermediate I

Table S3 Atomic coordinates of intermediate I

Standard orientation:

Center Number	Atomic Number	Atomic Type	Coordinates (Angstroms) X	Y	Z
1	30	0	0.821986	-0.271718	1.660378
2	8	0	-0.681800	0.052834	0.390080
3	7	0	1.992558	-1.521737	0.187080
4	7	0	0.480933	-2.393120	2.569078
5	6	0	-1.138622	-0.905224	-0.416650
6	6	0	-0.206735	-1.722440	-1.124723
7	6	0	-0.649683	-2.795408	-1.903254
8	6	0	-2.006633	-3.085545	-2.000058
9	6	0	-2.924495	-2.250631	-1.359105
10	6	0	-2.541281	-1.136935	-0.599342
11	6	0	-3.612148	-0.218841	0.053869
12	6	0	-3.572792	-0.419496	1.586124
13	6	0	-3.314087	1.283293	-0.219515
14	6	0	-5.021428	-0.500542	-0.513666
15	6	0	-5.305759	-0.213888	-1.862201
16	6	0	-6.574662	-0.413736	-2.401131

17	6	0	-7.608887	-0.909814	-1.602613
18	6	0	-7.348054	-1.204885	-0.266882
19	6	0	-6.070551	-1.003649	0.267342
20	6	0	1.262322	-1.364142	-1.117623
21	6	0	3.364335	-0.916151	0.096079
22	6	0	4.309866	-1.510731	-0.934411
23	6	0	4.407287	-0.937928	-2.211876
24	6	0	5.271120	-1.471025	-3.169895
25	6	0	6.058732	-2.582708	-2.863984
26	6	0	5.985860	-3.150972	-1.590703
27	6	0	5.121271	-2.615105	-0.634897
28	6	0	2.037169	-2.921589	0.668135
29	6	0	0.792227	-3.329959	1.464503
30	6	0	1.429029	-2.535061	3.689102
31	6	0	-0.891815	-2.623781	3.054586
32	6	0	1.711320	1.080248	2.936102
33	6	0	0.818584	1.617443	4.069858
34	8	0	2.367870	3.890060	-0.277166
35	6	0	1.720070	5.124258	0.109533
36	6	0	0.242639	4.991360	0.465818
37	6	0	1.949723	6.194738	-0.958838
38	8	0	-0.412800	3.886932	0.070826
39	8	0	-0.312098	5.856582	1.098643
40	6	0	0.160017	2.966452	-0.888499
41	6	0	1.677685	2.907859	-0.879637
42	6	0	-0.389054	3.260390	-2.286637
43	8	0	2.267723	1.978613	-1.389902
44	1	0	0.080443	-3.401031	-2.438393
45	1	0	-2.354753	-3.930348	-2.587673
46	1	0	-3.980661	-2.470561	-1.466764
47	1	0	-3.794110	-1.455670	1.865856
48	1	0	-2.573999	-0.167580	1.947252
49	1	0	-4.293823	0.234347	2.091389
50	1	0	-2.386754	1.582739	0.267581
51	1	0	-3.222553	1.485296	-1.291987
52	1	0	-4.134241	1.898859	0.167381
53	1	0	-4.513984	0.156600	-2.506646
54	1	0	-6.756413	-0.181746	-3.447745

55	1	0	-8.600932	-1.064548	-2.018796
56	1	0	-8.138213	-1.593668	0.370960
57	1	0	-5.906227	-1.243396	1.311862
58	1	0	1.382327	-0.309793	-1.386717
59	1	0	1.776833	-1.961807	-1.882278
60	1	0	3.221932	0.145745	-0.119815
61	1	0	3.805149	-0.991053	1.096462
62	1	0	3.813166	-0.058004	-2.443874
63	1	0	5.334756	-1.012173	-4.153052
64	1	0	6.734096	-2.996101	-3.608301
65	1	0	6.609008	-4.004872	-1.337850
66	1	0	5.092416	-3.052117	0.360709
67	1	0	2.922488	-3.026758	1.302133
68	1	0	2.166006	-3.627237	-0.165166
69	1	0	-0.072421	-3.353663	0.800791
70	1	0	0.943444	-4.351157	1.855801
71	1	0	1.397689	-3.551570	4.114352
72	1	0	1.176519	-1.815309	4.471954
73	1	0	2.448802	-2.323502	3.359124
74	1	0	-1.004804	-3.633893	3.481151
75	1	0	-1.596639	-2.506328	2.229667
76	1	0	-1.134161	-1.890347	3.829276
77	1	0	2.616767	0.628587	3.370319
78	1	0	2.075999	1.926781	2.335023
79	1	0	1.339066	2.342497	4.714687
80	1	0	0.462357	0.812845	4.729003
81	1	0	-0.074552	2.122489	3.680142
82	1	0	2.225336	5.418066	1.032258
83	1	0	1.463008	5.928288	-1.903082
84	1	0	1.539475	7.146763	-0.611105
85	1	0	3.021909	6.309096	-1.141282
86	1	0	-0.180525	1.978701	-0.557451
87	1	0	-0.026350	2.503734	-2.987945
88	1	0	-1.481072	3.223798	-2.257430
89	1	0	-0.082403	4.249230	-2.645551

Table S4 Thermochemical data of intermediate I

- Thermochemistry -

Temperature 298.150 Kelvin. Pressure 1.00000 Atm.

Zero-point correction=	0.755911 (Hartree/Particle)
Thermal correction to Energy=	0.801181
Thermal correction to Enthalpy=	0.802125
Thermal correction to Gibbs Free Energy=	0.672158
Sum of electronic and zero-point Energies=	-1911.890980
Sum of electronic and thermal Energies=	-1911.845710
Sum of electronic and thermal Enthalpies=	-1911.844766
Sum of electronic and thermal Free Energies=	-1911.974733

	E (Thermal)	CV	S
	KCal/Mol	Cal/Mol-Kelvin	Cal/Mol-Kelvin
Total	502.748	170.350	273.539

Figure S10 Chemical structure and ball and stick model of intermediate II

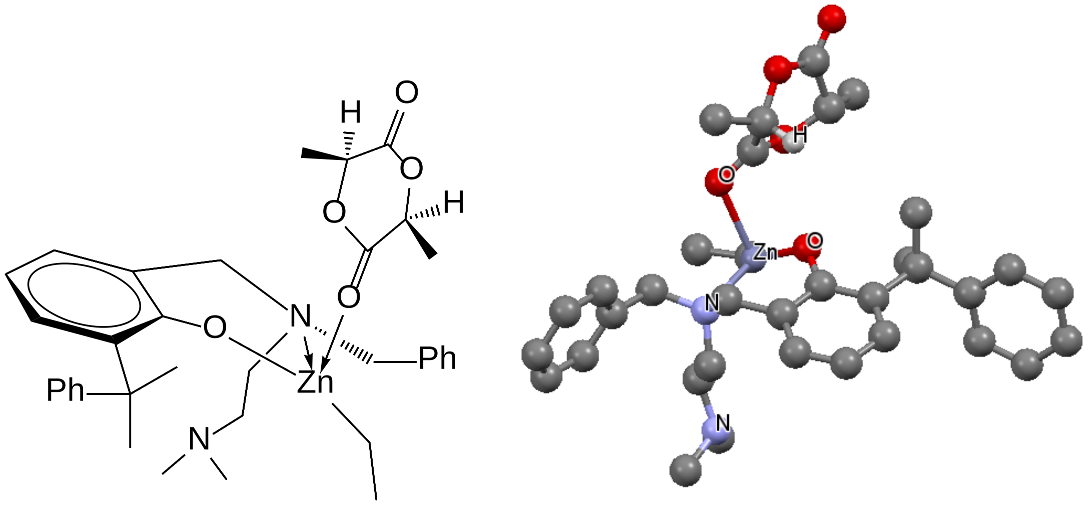

Table S5 Atomic coordinates of intermediate II

Standard orientation:

Center Number	Atomic Number	Atomic Type	Coordinates (Angstroms) X	Y	Z
1	30	0	0.629950	0.565530	1.253892
2	8	0	-0.824175	0.300496	-0.072318
3	7	0	2.213491	-0.365785	-0.079915
4	7	0	3.049054	-3.302108	2.185468
5	6	0	-0.867815	-0.795189	-0.834046
6	6	0	0.303754	-1.198628	-1.537192
7	6	0	0.278519	-2.305642	-2.390098
8	6	0	-0.893795	-3.028915	-2.574423
9	6	0	-2.043878	-2.641158	-1.884210
10	6	0	-2.068911	-1.557363	-0.997465
11	6	0	-3.392169	-1.133959	-0.306453
12	6	0	-3.202094	-0.862798	1.213770
13	6	0	-3.846573	0.188323	-0.969318
14	6	0	-4.476854	-2.226243	-0.419217
15	6	0	-4.288356	-3.465966	0.220237
16	6	0	-5.262544	-4.460917	0.185721

17	6	0	-6.465534	-4.245618	-0.492611
18	6	0	-6.669995	-3.027589	-1.135961
19	6	0	-5.686143	-2.033205	-1.099658
20	6	0	1.559181	-0.367846	-1.435653
21	6	0	3.370997	0.594316	-0.057782
22	6	0	4.523379	0.315743	-1.009068
23	6	0	4.536067	0.871877	-2.297277
24	6	0	5.592543	0.630083	-3.176219
25	6	0	6.664582	-0.170642	-2.777488
26	6	0	6.675366	-0.718367	-1.493561
27	6	0	5.615874	-0.472813	-0.618426
28	6	0	2.597295	-1.751838	0.301982
29	6	0	3.036128	-1.901263	1.765372
30	6	0	4.127437	-4.067972	1.572375
31	6	0	3.095970	-3.416209	3.638395
32	6	0	0.760087	0.749973	3.292589
33	6	0	2.031668	1.422997	3.835530
34	8	0	-1.154385	3.251958	1.117106
35	6	0	-2.541097	3.629735	0.866718
36	6	0	-2.589811	4.870235	-0.023953
37	6	0	-3.206163	3.840532	2.212694
38	8	0	-3.383760	5.764563	0.110698
39	8	0	-1.658729	4.907892	-1.009502
40	6	0	-0.833967	3.741545	-1.235141
41	6	0	-0.314550	3.191492	0.085188
42	6	0	0.269140	4.144377	-2.197941
43	8	0	0.797803	2.709424	0.225376
44	1	0	1.186172	-2.586794	-2.921868
45	1	0	-0.921551	-3.881585	-3.246821
46	1	0	-2.953323	-3.210121	-2.041417
47	1	0	-2.792078	-1.736664	1.729920
48	1	0	-2.518464	-0.028818	1.370184
49	1	0	-4.172231	-0.632240	1.670313
50	1	0	-3.040191	0.918005	-0.859673
51	1	0	-4.041015	0.060143	-2.039480
52	1	0	-4.752759	0.588516	-0.496607
53	1	0	-3.354742	-3.660449	0.740213
54	1	0	-5.081754	-5.407399	0.689329

55	1	0	-7.228689	-5.018989	-0.519559
56	1	0	-7.597564	-2.841736	-1.671965
57	1	0	-5.880109	-1.097647	-1.612422
58	1	0	1.333823	0.683622	-1.651797
59	1	0	2.283481	-0.714465	-2.184210
60	1	0	2.950254	1.580108	-0.276817
61	1	0	3.739789	0.625558	0.971465
62	1	0	3.713008	1.509601	-2.611131
63	1	0	5.581754	1.072794	-4.168634
64	1	0	7.489979	-0.358108	-3.458853
65	1	0	7.512320	-1.330789	-1.168766
66	1	0	5.645826	-0.888112	0.385696
67	1	0	3.373121	-2.115982	-0.386150
68	1	0	1.714927	-2.378900	0.156691
69	1	0	2.313944	-1.373853	2.396599
70	1	0	4.020341	-1.422453	1.943879
71	1	0	5.134423	-3.683120	1.834612
72	1	0	4.067995	-5.109492	1.905528
73	1	0	4.034103	-4.063547	0.482684
74	1	0	3.032095	-4.472016	3.923351
75	1	0	4.022910	-2.999911	4.081212
76	1	0	2.242425	-2.892319	4.080904
77	1	0	0.634498	-0.239227	3.756626
78	1	0	-0.119439	1.332111	3.602491
79	1	0	2.018948	1.527190	4.931273
80	1	0	2.938086	0.853916	3.586208
81	1	0	2.164930	2.430811	3.421444
82	1	0	-3.010799	2.794317	0.332004
83	1	0	-4.253304	4.111377	2.059943
84	1	0	-3.152174	2.920714	2.800590
85	1	0	-2.716768	4.649780	2.761292
86	1	0	-1.453630	2.947333	-1.677208
87	1	0	0.882119	3.274932	-2.445645
88	1	0	-0.176063	4.540886	-3.113994
89	1	0	0.908612	4.911165	-1.752380

Table S6 Thermochemical data of intermediate II

- Thermochemistry -

Temperature 298.150 Kelvin. Pressure 1.00000 Atm.

Zero-point correction=	0.754331 (Hartree/Particle)
Thermal correction to Energy=	0.800045
Thermal correction to Enthalpy=	0.800989
Thermal correction to Gibbs Free Energy=	0.668253
Sum of electronic and zero-point Energies=	-1911.883204
Sum of electronic and thermal Energies=	-1911.837491
Sum of electronic and thermal Enthalpies=	-1911.836547
Sum of electronic and thermal Free Energies=	-1911.969283

	E (Thermal)	CV	S
	KCal/Mol	Cal/Mol-Kelvin	Cal/Mol-Kelvin
Total	502.036	170.853	279.367

Figure S11 Chemical structure and ball and stick model of intermediate III

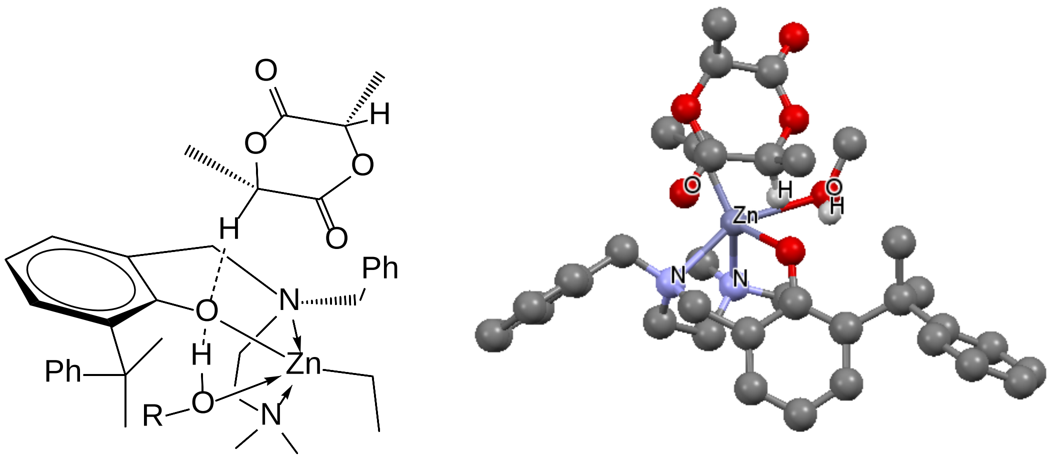

Table S7 Atomic coordinates of intermediate III

Standard orientation:

Center Number	Atomic Number	Atomic Type	Coordinates (Angstroms) X	Y	Z
1	30	0	0.942468	0.098417	1.565700
2	8	0	-0.761010	0.081033	0.369388
3	7	0	1.935433	-1.529462	0.240181
4	7	0	0.676942	-1.785954	2.904980
5	6	0	-1.278622	-1.019834	-0.187026
6	6	0	-0.391907	-1.986600	-0.754100
7	6	0	-0.873076	-3.213429	-1.215858
8	6	0	-2.229056	-3.516997	-1.144281
9	6	0	-3.107015	-2.537098	-0.686578
10	6	0	-2.687439	-1.267789	-0.256353
11	6	0	-3.778952	-0.243147	0.165429
12	6	0	-3.983377	-0.337651	1.695462
13	6	0	-3.415860	1.226569	-0.188215
14	6	0	-5.097880	-0.520725	-0.596205
15	6	0	-6.317283	-0.764573	0.047997
16	6	0	-7.500284	-0.948204	-0.677525
17	6	0	-7.490673	-0.892451	-2.068756
18	6	0	-6.281802	-0.656124	-2.730066
19	6	0	-5.108344	-0.474696	-2.002869

20	6	0	1.065403	-1.649616	-0.972042
21	6	0	3.283694	-1.003825	-0.148115
22	6	0	4.092327	-1.835935	-1.130621
23	6	0	4.057221	-1.533386	-2.500671
24	6	0	4.794218	-2.285465	-3.417173
25	6	0	5.584554	-3.349535	-2.978333
26	6	0	5.642690	-3.649961	-1.615927
27	6	0	4.905633	-2.895538	-0.701741
28	6	0	2.033614	-2.775654	1.028114
29	6	0	0.878832	-2.956692	2.018416
30	6	0	-0.652026	-1.874038	3.543283
31	6	0	1.714122	-1.718595	3.951740
32	6	0	2.293454	1.641235	1.905432
33	6	0	3.295893	1.477926	3.061734
34	8	0	2.490848	3.297477	-1.463459
35	6	0	2.142949	4.523688	-0.780626
36	6	0	0.821340	4.488262	-0.022144
37	6	0	2.199780	5.699773	-1.757145
38	8	0	0.593403	5.289595	0.852922
39	8	0	-0.084471	3.552103	-0.347693
40	6	0	0.104293	2.651558	-1.466395
41	6	0	1.555114	2.415629	-1.851957
42	6	0	-0.722589	3.125362	-2.663540
43	8	0	1.871448	1.445388	-2.506200
44	8	0	-0.977934	1.147753	2.791597
45	6	0	-1.199764	2.521570	3.112566
46	1	0	-0.171345	-3.930035	-1.639666
47	1	0	-2.605562	-4.479722	-1.477848
48	1	0	-4.169372	-2.757736	-0.685268
49	1	0	-4.309311	-1.340542	1.992931
50	1	0	-3.049432	-0.120547	2.218005
51	1	0	-4.729858	0.385620	2.046235
52	1	0	-2.559654	1.589173	0.377966
53	1	0	-3.175298	1.329222	-1.251352
54	1	0	-4.276575	1.871639	0.021810
55	1	0	-6.362870	-0.816173	1.130069
56	1	0	-8.429344	-1.135831	-0.144740
57	1	0	-8.408535	-1.034805	-2.633111

58	1	0	-6.252660	-0.616603	-3.816242
59	1	0	-4.175552	-0.313702	-2.535835
60	1	0	1.140186	-0.683558	-1.482497
61	1	0	1.497109	-2.403499	-1.645025
62	1	0	3.118435	-0.011656	-0.573876
63	1	0	3.846276	-0.863254	0.780823
64	1	0	3.459743	-0.691985	-2.842979
65	1	0	4.755858	-2.034512	-4.473973
66	1	0	6.160989	-3.933406	-3.691055
67	1	0	6.269525	-4.464966	-1.263252
68	1	0	4.979318	-3.124965	0.358921
69	1	0	2.979588	-2.745790	1.577732
70	1	0	2.078367	-3.663407	0.379503
71	1	0	-0.048787	-3.116184	1.469037
72	1	0	1.068382	-3.862664	2.619928
73	1	0	-0.725093	-2.767595	4.184495
74	1	0	-1.422353	-1.928481	2.771169
75	1	0	-0.828955	-0.977834	4.139076
76	1	0	1.705899	-2.623678	4.581177
77	1	0	1.532311	-0.847230	4.585600
78	1	0	2.705527	-1.608648	3.506478
79	1	0	2.857334	1.835838	0.980481
80	1	0	1.697758	2.546929	2.088838
81	1	0	3.961285	2.347850	3.179646
82	1	0	2.788386	1.340911	4.026115
83	1	0	3.950139	0.605167	2.922775
84	1	0	2.917668	4.640358	-0.019984
85	1	0	1.432022	5.613559	-2.533253
86	1	0	2.041148	6.633394	-1.210333
87	1	0	3.180459	5.728321	-2.240273
88	1	0	-0.277261	1.694072	-1.098494
89	1	0	-0.652434	2.387745	-3.467972
90	1	0	-1.768787	3.226462	-2.362723
91	1	0	-0.373496	4.092240	-3.042440
92	1	0	-1.303950	0.953751	1.884266
93	1	0	-0.716335	2.709623	4.074551
94	1	0	-0.771927	3.199131	2.365170
95	1	0	-2.272992	2.732532	3.214355

Table S8 Thermochemical data of intermediate III

- Thermochemistry -

Temperature 298.150 Kelvin. Pressure 1.00000 Atm.

Zero-point correction=	0.810483 (Hartree/Particle)
Thermal correction to Energy=	0.859838
Thermal correction to Enthalpy=	0.860783
Thermal correction to Gibbs Free Energy=	0.723338
Sum of electronic and zero-point Energies=	-2027.564170
Sum of electronic and thermal Energies=	-2027.514815
Sum of electronic and thermal Enthalpies=	-2027.513871
Sum of electronic and thermal Free Energies=	-2027.651315

	E (Thermal)	CV	S
	KCal/Mol	Cal/Mol-Kelvin	Cal/Mol-Kelvin
Total	539.557	183.472	289.276

Figure S12 Chemical structure and ball and stick model of intermediate IV

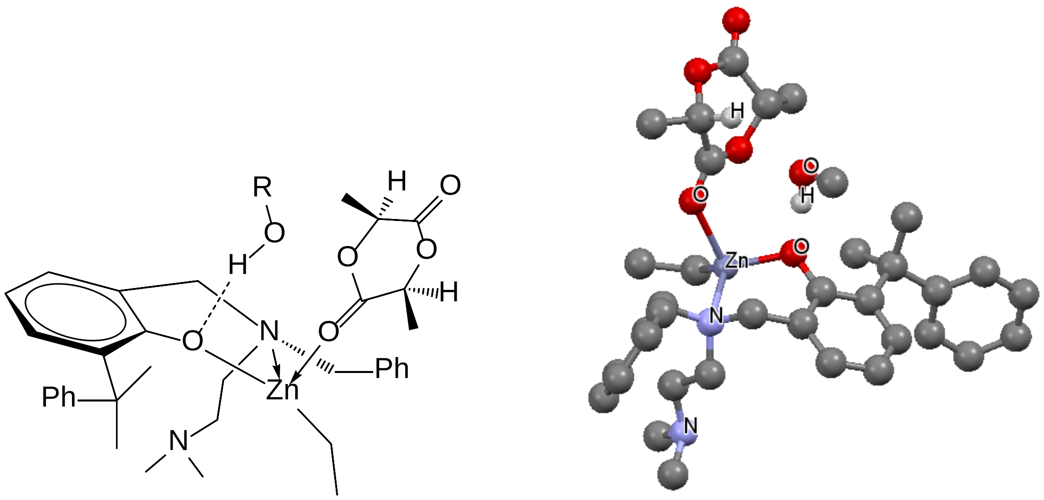

Table S9 Atomic coordinates of intermediate IV

Standard orientation:

Center Number	Atomic Number	Atomic Type	Coordinates (Angstroms) X	Y	Z
1	30	0	0.513451	0.303203	1.180579
2	8	0	-0.849901	0.282294	-0.291209
3	7	0	1.993770	-0.947673	-0.068582
4	7	0	2.305766	-3.835591	2.381246
5	6	0	-1.083448	-0.883035	-0.922070
6	6	0	0.013239	-1.546821	-1.534443
7	6	0	-0.187111	-2.719213	-2.267462
8	6	0	-1.465204	-3.249201	-2.406498
9	6	0	-2.539206	-2.609301	-1.786077
10	6	0	-2.391136	-1.444106	-1.019716
11	6	0	-3.635323	-0.755749	-0.396340
12	6	0	-3.382617	-0.256246	1.055073
13	6	0	-3.972209	0.481150	-1.263076
14	6	0	-4.828619	-1.733017	-0.312623
15	6	0	-4.741272	-2.869253	0.513412
16	6	0	-5.809837	-3.750979	0.657366
17	6	0	-7.008315	-3.522130	-0.024659
18	6	0	-7.113331	-2.406160	-0.851024

19	6	0	-6.035455	-1.524937	-0.992803
20	6	0	1.388998	-0.932127	-1.446508
21	6	0	3.292996	-0.190834	-0.060858
22	6	0	4.399240	-0.691942	-0.975639
23	6	0	4.524368	-0.193291	-2.281360
24	6	0	5.542985	-0.634203	-3.127145
25	6	0	6.464040	-1.582088	-2.677007
26	6	0	6.362625	-2.077608	-1.375964
27	6	0	5.341555	-1.632775	-0.534290
28	6	0	2.141278	-2.353230	0.399690
29	6	0	2.552858	-2.486648	1.872246
30	6	0	3.216820	-4.826981	1.822527
31	6	0	2.342836	-3.863034	3.838687
32	6	0	0.372721	0.283166	3.229984
33	6	0	1.599037	0.802601	3.999356
34	8	0	-0.571739	3.312435	0.913274
35	6	0	-1.511751	4.346881	0.475990
36	6	0	-0.829869	5.711306	0.525025
37	6	0	-2.731073	4.260352	1.371674
38	8	0	-1.381254	6.728169	0.858910
39	8	0	0.477863	5.717543	0.162064
40	6	0	1.043588	4.525059	-0.428206
41	6	0	0.634079	3.292923	0.364190
42	6	0	2.547776	4.724618	-0.500703
43	8	0	1.365630	2.321747	0.513563
44	8	0	-0.865667	2.449294	-1.914365
45	6	0	-0.984781	2.080107	-3.279511
46	1	0	0.665009	-3.206087	-2.738660
47	1	0	-1.630811	-4.152764	-2.986321
48	1	0	-3.528595	-3.038835	-1.897253
49	1	0	-3.008935	-1.057266	1.701235
50	1	0	-2.649719	0.549461	1.062773
51	1	0	-4.324863	0.104582	1.484043
52	1	0	-3.118329	1.162683	-1.277130
53	1	0	-4.198987	0.199713	-2.297394
54	1	0	-4.833176	1.027206	-0.857804
55	1	0	-3.814022	-3.074413	1.040864
56	1	0	-5.706483	-4.619398	1.303330

57	1	0	-7.844884	-4.206971	0.086675
58	1	0	-8.036038	-2.212575	-1.392701
59	1	0	-6.152582	-0.667035	-1.645447
60	1	0	1.348913	0.123452	-1.741079
61	1	0	2.061810	-1.448963	-2.143292
62	1	0	3.044499	0.840578	-0.323627
63	1	0	3.647494	-0.176401	0.974015
64	1	0	3.820031	0.555556	-2.636118
65	1	0	5.620961	-0.232612	-4.133967
66	1	0	7.260250	-1.924874	-3.332186
67	1	0	7.083123	-2.805218	-1.011895
68	1	0	5.285656	-2.011525	0.483016
69	1	0	2.843408	-2.882384	-0.260496
70	1	0	1.166351	-2.832444	0.287489
71	1	0	1.944381	-1.793916	2.461026
72	1	0	3.610782	-2.192006	2.025610
73	1	0	4.279649	-4.625235	2.069211
74	1	0	2.962092	-5.817033	2.215206
75	1	0	3.122238	-4.868535	0.733708
76	1	0	2.084589	-4.867508	4.191356
77	1	0	3.335639	-3.600772	4.256221
78	1	0	1.606957	-3.160416	4.242745
79	1	0	0.115775	-0.728369	3.576460
80	1	0	-0.500622	0.904093	3.477450
81	1	0	1.442136	0.815797	5.088842
82	1	0	2.491334	0.187032	3.818411
83	1	0	1.858732	1.827514	3.703393
84	1	0	-1.760231	4.098629	-0.563429
85	1	0	-3.461303	5.009530	1.057076
86	1	0	-3.177183	3.265544	1.294519
87	1	0	-2.463331	4.457023	2.413527
88	1	0	0.618124	4.384812	-1.429720
89	1	0	3.015884	3.865093	-0.985703
90	1	0	2.765919	5.625376	-1.080263
91	1	0	2.972627	4.837332	0.500627
92	1	0	-0.899146	1.627920	-1.354174
93	1	0	-0.180775	1.400464	-3.598705
94	1	0	-1.947709	1.596102	-3.492864

95	1	0	-0.919277	2.993427	-3.879942

Table S10 Thermochemical data of intermediate IV

- Thermochemistry -

Temperature 298.150 Kelvin. Pressure 1.00000 Atm.

Zero-point correction=	0.808996 (Hartree/Particle)
Thermal correction to Energy=	0.858664
Thermal correction to Enthalpy=	0.859608
Thermal correction to Gibbs Free Energy=	0.719212
Sum of electronic and zero-point Energies=	-2027.568737
Sum of electronic and thermal Energies=	-2027.519068
Sum of electronic and thermal Enthalpies=	-2027.518124
Sum of electronic and thermal Free Energies=	-2027.658521

	E (Thermal)	CV	S
	KCal/Mol	Cal/Mol-Kelvin	Cal/Mol-Kelvin
Total	538.820	183.556	295.489

Figure S13 Chemical structure and ball and stick model of intermediate V

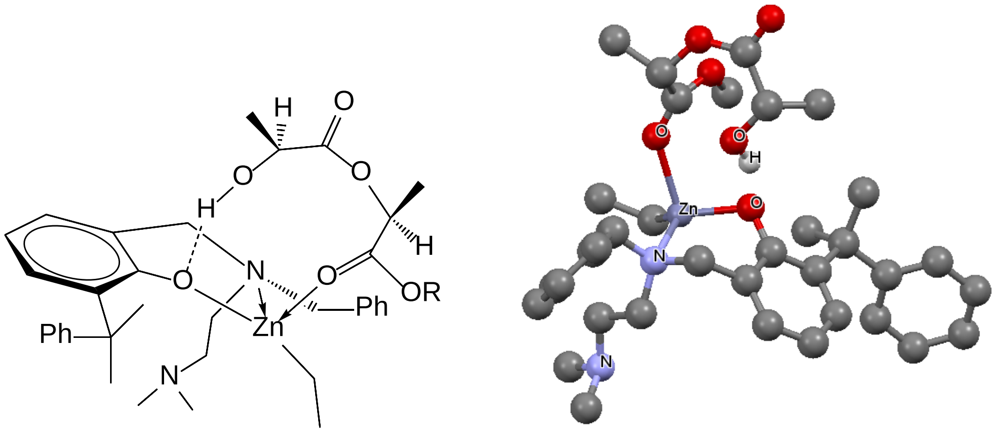

Table S11 Atomic coordinates of intermediate V

Standard orientation:

Center Number	Atomic Number	Atomic Type	Coordinates (Angstroms) X	Y	Z
1	30	0	0.347294	-0.255157	1.424484
2	8	0	-0.881979	0.619753	0.074925
3	7	0	1.580031	-1.282990	-0.199064
4	7	0	1.269494	-4.747575	1.309012
5	6	0	-1.385705	-0.250940	-0.830834
6	6	0	-0.472527	-0.964456	-1.654688
7	6	0	-0.939200	-1.816317	-2.659410
8	6	0	-2.303564	-1.979021	-2.866085
9	6	0	-3.200219	-1.309213	-2.033792
10	6	0	-2.788993	-0.464654	-0.992273
11	6	0	-3.841490	0.243530	-0.095563
12	6	0	-3.529893	0.032569	1.412960
13	6	0	-3.776646	1.761344	-0.380971
14	6	0	-5.264800	-0.309842	-0.335806
15	6	0	-5.586472	-1.622944	0.056530
16	6	0	-6.868702	-2.142446	-0.107999
17	6	0	-7.878562	-1.359526	-0.673469
18	6	0	-7.580300	-0.059548	-1.074265
19	6	0	-6.290042	0.455257	-0.908218
20	6	0	1.013428	-0.751277	-1.489628

21	6	0	3.000377	-0.819554	-0.028750
22	6	0	3.988307	-1.181584	-1.126026
23	6	0	4.207396	-0.304396	-2.199322
24	6	0	5.121725	-0.614224	-3.206711
25	6	0	5.840685	-1.810156	-3.155179
26	6	0	5.644436	-2.687076	-2.087009
27	6	0	4.729005	-2.371896	-1.081057
28	6	0	1.449112	-2.765927	-0.173727
29	6	0	1.850599	-3.414079	1.159095
30	6	0	1.830097	-5.716726	0.374983
31	6	0	1.391942	-5.221380	2.682705
32	6	0	-0.020968	-0.974229	3.316274
33	6	0	1.160459	-0.913052	4.299265
34	8	0	0.811151	2.521232	-0.908557
35	6	0	0.519849	3.771183	-1.502200
36	6	0	1.289238	4.946575	-0.861143
37	6	0	-0.974932	4.100695	-1.516509
38	8	0	1.117029	6.065971	-1.280052
39	8	0	2.183888	4.789851	0.157262
40	6	0	2.620481	3.560037	0.745760
41	6	0	1.509051	2.815244	1.473033
42	6	0	3.731091	3.939776	1.728376
43	8	0	1.596337	1.618572	1.741045
44	8	0	0.529117	3.606085	1.882372
45	6	0	-0.558143	2.995717	2.614246
46	1	0	-0.220727	-2.340372	-3.287117
47	1	0	-2.672828	-2.624832	-3.657654
48	1	0	-4.260993	-1.456788	-2.198069
49	1	0	-3.481425	-1.028420	1.675356
50	1	0	-2.571830	0.482168	1.671928
51	1	0	-4.316486	0.492904	2.022333
52	1	0	-2.773419	2.121455	-0.150318
53	1	0	-3.980839	1.984340	-1.433696
54	1	0	-4.495486	2.314316	0.235905
55	1	0	-4.815899	-2.257761	0.483809
56	1	0	-7.079149	-3.161728	0.206381
57	1	0	-8.880696	-1.760394	-0.800806
58	1	0	-8.350747	0.565180	-1.519525

59	1	0	-6.096483	1.471378	-1.232761
60	1	0	1.248896	0.316231	-1.508370
61	1	0	1.540120	-1.222444	-2.329663
62	1	0	2.951286	0.267631	0.071738
63	1	0	3.351431	-1.209334	0.931309
64	1	0	3.659023	0.633690	-2.241908
65	1	0	5.276720	0.080643	-4.027746
66	1	0	6.555411	-2.052405	-3.936971
67	1	0	6.209430	-3.613747	-2.031084
68	1	0	4.600172	-3.052973	-0.243806
69	1	0	2.022375	-3.191574	-1.009722
70	1	0	0.395541	-2.995657	-0.350396
71	1	0	1.466424	-2.794911	1.975153
72	1	0	2.953574	-3.446100	1.271973
73	1	0	2.922101	-5.861950	0.505573
74	1	0	1.341395	-6.685781	0.520613
75	1	0	1.648544	-5.406740	-0.658154
76	1	0	0.887107	-6.188412	2.781295
77	1	0	2.443621	-5.351725	3.008223
78	1	0	0.908748	-4.513822	3.364102
79	1	0	-0.404726	-2.002424	3.254080
80	1	0	-0.856100	-0.380892	3.717795
81	1	0	0.889510	-1.252695	5.310651
82	1	0	2.002056	-1.539796	3.972786
83	1	0	1.552430	0.107684	4.398229
84	1	0	0.008590	1.985459	-0.670884
85	1	0	0.878180	3.754681	-2.542957
86	1	0	-1.140275	5.063089	-2.004797
87	1	0	-1.520546	3.323141	-2.059833
88	1	0	-1.368394	4.156147	-0.496046
89	1	0	3.002450	2.881287	-0.017733
90	1	0	4.128521	3.043671	2.214560
91	1	0	4.538706	4.439949	1.186942
92	1	0	3.348216	4.624443	2.491114
93	1	0	-1.171861	3.827883	2.957097
94	1	0	-1.126149	2.345840	1.945467
95	1	0	-0.172108	2.422633	3.459950

Table S12 Thermochemical data of intermediate V

- Thermochemistry -

Temperature 298.150 Kelvin. Pressure 1.00000 Atm.

Zero-point correction= 0.810238 (Hartree/Particle)
Thermal correction to Energy= 0.859079
Thermal correction to Enthalpy= 0.860023
Thermal correction to Gibbs Free Energy= 0.723546
Sum of electronic and zero-point Energies= -2027.558486
Sum of electronic and thermal Energies= -2027.509646
Sum of electronic and thermal Enthalpies= -2027.508702
Sum of electronic and thermal Free Energies= -2027.645178

	E (Thermal)	CV	S
	KCal/Mol	Cal/Mol-Kelvin	Cal/Mol-Kelvin
Total	539.080	182.965	287.239

Figure S14 Chemical structure and ball and stick model of intermediate VI

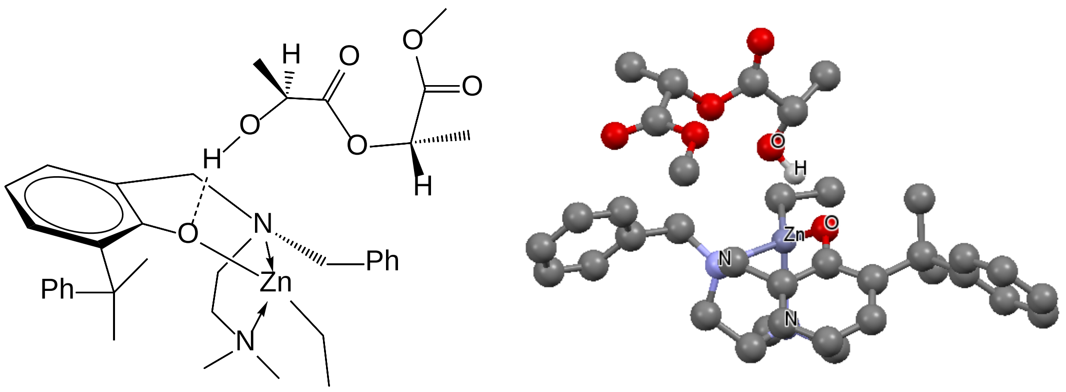

Table S13 Atomic coordinates of intermediate VI

Standard orientation:

Center Number	Atomic Number	Atomic Type	Coordinates (Angstroms) X	Y	Z
1	30	0	0.488008	2.209464	1.213315
2	8	0	1.279549	0.402467	0.785846
3	7	0	-1.022883	2.027040	-0.438734
4	7	0	1.377179	3.761009	-0.296371
5	6	0	1.586036	0.108196	-0.487462
6	6	0	0.618537	0.348967	-1.504867
7	6	0	0.940588	0.139560	-2.848858
8	6	0	2.209377	-0.301556	-3.211163
9	6	0	3.139466	-0.590147	-2.210907
10	6	0	2.858575	-0.436406	-0.845353
11	6	0	3.917295	-0.808931	0.229344
12	6	0	4.439563	0.492504	0.881517
13	6	0	3.293952	-1.676288	1.360596
14	6	0	5.071788	-1.636618	-0.377579
15	6	0	4.822466	-2.929731	-0.874880
16	6	0	5.843872	-3.723402	-1.391950
17	6	0	7.157191	-3.246830	-1.427092
18	6	0	7.425096	-1.968605	-0.943796
19	6	0	6.394138	-1.175484	-0.428045
20	6	0	-0.803134	0.707918	-1.124191

21	6	0	-2.367983	2.043096	0.233605
22	6	0	-3.581632	1.903151	-0.668150
23	6	0	-4.124779	0.635987	-0.928577
24	6	0	-5.244062	0.486406	-1.749439
25	6	0	-5.840642	1.611800	-2.322754
26	6	0	-5.320242	2.881278	-2.062590
27	6	0	-4.202529	3.024014	-1.238187
28	6	0	-0.838552	3.192008	-1.333058
29	6	0	0.634541	3.552268	-1.561453
30	6	0	1.098304	5.090526	0.277604
31	6	0	2.827349	3.615007	-0.522818
32	6	0	0.124652	3.008976	3.074818
33	6	0	1.360036	3.176787	3.978009
34	8	0	-0.934718	-0.867647	1.769449
35	6	0	-0.791913	-2.198438	2.217211
36	6	0	-0.236229	-2.278822	3.642682
37	6	0	-2.124847	-2.945464	2.124501
38	8	0	-2.365903	-3.953075	2.753878
39	8	0	-2.980043	-2.392877	1.230363
40	6	0	-4.232752	-3.085250	1.015841
41	6	0	-5.379267	-2.276965	1.599653
42	6	0	-4.358779	-3.290285	-0.493977
43	8	0	-5.286956	-2.903499	-1.173795
44	8	0	-3.310887	-3.979790	-0.973256
45	6	0	-3.317992	-4.218010	-2.391147
46	1	0	0.184707	0.323207	-3.611041
47	1	0	2.469906	-0.446045	-4.255887
48	1	0	4.114976	-0.959794	-2.505867
49	1	0	4.912912	1.153317	0.146931
50	1	0	3.601043	1.025119	1.334938
51	1	0	5.170459	0.273705	1.669024
52	1	0	2.543496	-1.104163	1.905979
53	1	0	2.816783	-2.577486	0.962236
54	1	0	4.079791	-1.994100	2.055455
55	1	0	3.807626	-3.316765	-0.871324
56	1	0	5.613736	-4.717373	-1.767676
57	1	0	7.956894	-3.864772	-1.826861
58	1	0	8.439869	-1.578682	-0.963464

59	1	0	6.641048	-0.186025	-0.059693
60	1	0	-1.174430	-0.045271	-0.423380
61	1	0	-1.435767	0.666676	-2.021368
62	1	0	-2.352370	1.225287	0.960405
63	1	0	-2.424824	2.982424	0.795254
64	1	0	-3.672224	-0.238624	-0.468918
65	1	0	-5.646432	-0.507266	-1.923651
66	1	0	-6.714965	1.501363	-2.958774
67	1	0	-5.790591	3.762258	-2.491797
68	1	0	-3.821721	4.019974	-1.022155
69	1	0	-1.355159	4.042153	-0.875247
70	1	0	-1.315804	3.024731	-2.309926
71	1	0	1.126557	2.745725	-2.104990
72	1	0	0.683362	4.456216	-2.192207
73	1	0	1.431349	5.894831	-0.398339
74	1	0	1.617089	5.191365	1.234271
75	1	0	0.028575	5.209633	0.465550
76	1	0	3.204156	4.370700	-1.231113
77	1	0	3.038978	2.619103	-0.917906
78	1	0	3.357565	3.734489	0.426459
79	1	0	-0.383238	3.979980	2.969159
80	1	0	-0.600595	2.352887	3.576625
81	1	0	1.108860	3.583570	4.969743
82	1	0	2.104080	3.857586	3.539954
83	1	0	1.870560	2.219936	4.147149
84	1	0	-0.051752	-0.530033	1.476406
85	1	0	-0.119155	-2.757426	1.543167
86	1	0	-0.174644	-3.316296	3.981300
87	1	0	0.762799	-1.832701	3.671295
88	1	0	-0.883059	-1.721692	4.328292
89	1	0	-4.158554	-4.066155	1.495571
90	1	0	-5.423310	-1.278828	1.156172
91	1	0	-6.330508	-2.780549	1.403907
92	1	0	-5.246283	-2.182976	2.681322
93	1	0	-3.315123	-3.270598	-2.936577
94	1	0	-2.407968	-4.781631	-2.596362
95	1	0	-4.202255	-4.793176	-2.678149

Table S14 Thermochemical data of intermediate VI

- Thermochemistry -

Temperature 298.150 Kelvin. Pressure 1.00000 Atm.

Zero-point correction= 0.810803 (Hartree/Particle)
Thermal correction to Energy= 0.859879
Thermal correction to Enthalpy= 0.860823
Thermal correction to Gibbs Free Energy= 0.719751
Sum of electronic and zero-point Energies= -2027.581211
Sum of electronic and thermal Energies= -2027.532135
Sum of electronic and thermal Enthalpies= -2027.531191
Sum of electronic and thermal Free Energies= -2027.672263

	E (Thermal)	CV	S
	KCal/Mol	Cal/Mol-Kelvin	Cal/Mol-Kelvin
Total	539.582	182.600	296.912

Table S15 Atomic coordinates of methanol

Standard orientation:

Center Number	Atomic Number	Atomic Type	Coordinates (Angstroms) X	Y	Z
1	8	0	-0.749287	0.122444	-0.000033
2	6	0	0.662366	-0.019619	0.000015
3	1	0	-1.133995	-0.766454	0.000108
4	1	0	1.037345	-0.543424	-0.893166
5	1	0	1.079433	0.991085	-0.000195
6	1	0	1.037321	-0.543043	0.893429

Table S16 Thermochemical data of methanol

\- Thermochemistry -

Temperature 298.150 Kelvin. Pressure 1.00000 Atm.

Zero-point correction=	0.051470 (Hartree/Particle)
Thermal correction to Energy=	0.054763
Thermal correction to Enthalpy=	0.055708
Thermal correction to Gibbs Free Energy=	0.028746
Sum of electronic and zero-point Energies=	-115.662935
Sum of electronic and thermal Energies=	-115.659642
Sum of electronic and thermal Enthalpies=	-115.658697
Sum of electronic and thermal Free Energies=	-115.685659

	E (Thermal) KCal/Mol	CV Cal/Mol-Kelvin	S Cal/Mol-Kelvin
Total	34.365	8.672	56.745

Figure S15 Chemical structure and ball and stick model of $L^{1'}$ZnEt

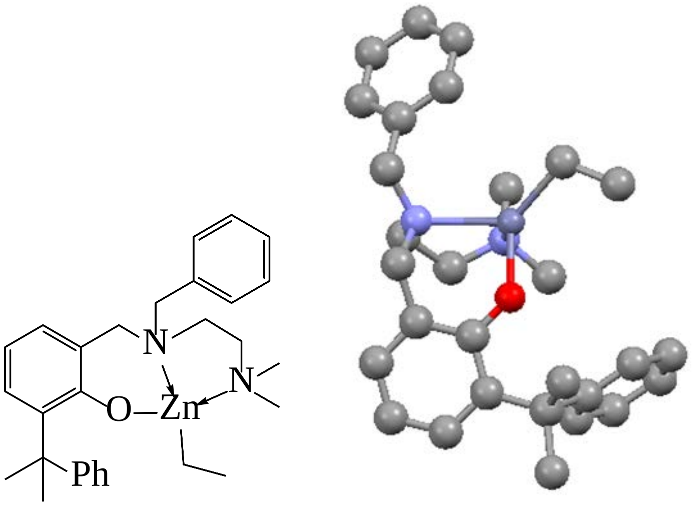

Table S17 Atomic coordinates of $L^{1'}$ZnEt

Standard orientation:

Center Number	Atomic Number	Atomic Type	Coordinates (Angstroms) X	Y	Z
1	30	0	-0.757455	-0.618214	-0.762988
2	8	0	1.013296	0.254245	-1.013582
3	7	0	-1.783255	1.368428	-0.154319
4	7	0	-0.832238	-0.874336	1.498676
5	6	0	1.467474	1.395586	-0.539825
6	6	0	2.870099	1.607096	-0.321224
7	6	0	3.274997	2.807785	0.268947
8	1	0	4.329802	2.978517	0.456816
9	6	0	2.375629	3.819461	0.623213
10	6	0	1.027089	3.648553	0.333010
11	1	0	0.318000	4.448885	0.541029
12	6	0	0.567135	2.465874	-0.256145
13	6	0	3.893167	0.543407	-0.779030
14	6	0	3.691411	0.266201	-2.288477
15	1	0	2.693589	-0.122090	-2.493911
16	1	0	4.441565	-0.441618	-2.661361
17	1	0	3.816938	1.202843	-2.842970
18	6	0	5.355386	1.039411	-0.628311

19	1	0	6.037704	0.263378	-0.991782
20	1	0	5.628882	1.252454	0.409592
21	1	0	5.525040	1.944796	-1.222262
22	6	0	3.773931	-0.731794	0.083212
23	6	0	3.578997	-2.009648	-0.458072
24	1	0	3.425627	-2.128541	-1.524637
25	6	0	3.573464	-3.147805	0.354348
26	1	0	3.422620	-4.125332	-0.097660
27	6	0	3.766764	-3.036436	1.731608
28	1	0	3.774002	-3.922710	2.361050
29	6	0	3.952289	-1.768947	2.289848
30	1	0	4.101408	-1.660434	3.361748
31	6	0	3.948293	-0.636470	1.475093
32	1	0	4.082834	0.343128	1.926901
33	6	0	-0.852728	2.393934	-0.753892
34	1	0	-0.847291	2.180239	-1.831470
35	1	0	-1.316566	3.384564	-0.621382
36	6	0	-3.132367	1.649018	-0.745147
37	1	0	-3.002347	1.599551	-1.831546
38	1	0	-3.412673	2.688220	-0.506627
39	6	0	-4.263356	0.731573	-0.330480
40	6	0	-5.193299	1.144378	0.635567
41	1	0	-5.088131	2.125790	1.093353
42	6	0	-6.258007	0.320857	1.006875
43	1	0	-6.968234	0.660313	1.756027
44	6	0	-6.413207	-0.930165	0.407431
45	1	0	-7.243334	-1.571940	0.689914
46	6	0	-5.503597	-1.346764	-0.567565
47	1	0	-5.625292	-2.312609	-1.050338
48	6	0	-4.440085	-0.521204	-0.936061
49	1	0	-3.745068	-0.851800	-1.701341
50	6	0	-1.811696	1.433360	1.328787
51	1	0	-2.812083	1.147968	1.659043
52	1	0	-1.639019	2.462546	1.676620
53	6	0	-0.777308	0.518135	1.996729
54	1	0	0.228007	0.895020	1.803174
55	1	0	-0.939375	0.545384	3.088498
56	6	0	-2.053306	-1.573148	1.937057

57	1	0	-2.110089	-1.625156	3.036820
58	1	0	-2.044638	-2.591131	1.538253
59	1	0	-2.945677	-1.071816	1.558250
60	6	0	0.358826	-1.621864	1.946118
61	1	0	0.407133	-1.681078	3.045312
62	1	0	1.261605	-1.137497	1.572982
63	1	0	0.320481	-2.638045	1.544419
64	6	0	-1.431610	-2.107441	-2.020201
65	1	0	-2.174763	-2.736817	-1.508468
66	1	0	-1.967518	-1.646929	-2.865078
67	6	0	-0.302794	-2.994812	-2.576297
68	1	0	-0.670125	-3.763929	-3.274026
69	1	0	0.232651	-3.522392	-1.775651
70	1	0	0.445838	-2.402456	-3.117066
71	1	0	2.734682	4.735770	1.082867

Figure S16 Chemical structure and ball and stick model of $L^{2'}$ZnEt

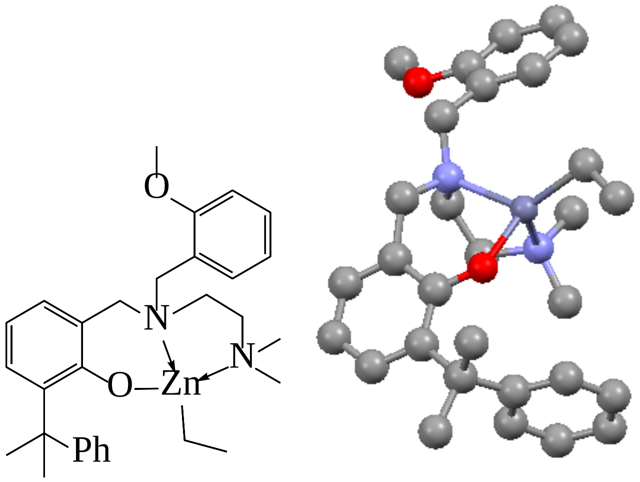

Table S18 Atomic coordinates of $L^{2'}$ZnEt

Standard orientation:

Center	Atomic	Atomic	Coordinates (Angstroms)		
Number	Number	Type	X	Y	Z
1	30	0	-0.308587	-0.914167	-0.782513
2	8	0	1.381362	0.103238	-1.048460
3	7	0	-1.562074	0.990735	-0.464315
4	7	0	-0.478690	-0.923703	1.489808
5	6	0	1.682912	1.338892	-0.708719
6	6	0	3.041231	1.732523	-0.462538
7	6	0	3.283875	3.034464	-0.015299
8	1	0	4.302706	3.344499	0.192080
9	6	0	2.264008	3.974126	0.169820
10	6	0	0.959034	3.617741	-0.149946
11	1	0	0.158414	4.352491	-0.074350
12	6	0	0.659262	2.327727	-0.600904
13	6	0	4.195055	0.741766	-0.735856
14	6	0	4.108948	0.257349	-2.203353
15	1	0	3.170693	-0.262564	-2.398201
16	1	0	4.949295	-0.403567	-2.448095
17	1	0	4.166887	1.125145	-2.869823

18	6	0	5.586209	1.412952	-0.589741
19	1	0	6.365412	0.679377	-0.823858
20	1	0	5.779522	1.779914	0.422939
21	1	0	5.691575	2.252638	-1.286324
22	6	0	4.163557	-0.420929	0.279578
23	6	0	4.142713	-1.770463	-0.097728
24	1	0	4.066974	-2.038663	-1.145355
25	6	0	4.211890	-2.790733	0.856051
26	1	0	4.196318	-3.828075	0.529935
27	6	0	4.307239	-2.486083	2.214235
28	1	0	4.372387	-3.279706	2.954287
29	6	0	4.319186	-1.145784	2.609127
30	1	0	4.389539	-0.887314	3.663286
31	6	0	4.241473	-0.132529	1.653422
32	1	0	4.239596	0.905004	1.977890
33	6	0	-0.718204	2.040399	-1.141426
34	1	0	-0.633812	1.711043	-2.186407
35	1	0	-1.291622	2.980837	-1.144962
36	6	0	-2.891946	1.026776	-1.162524
37	1	0	-2.682578	0.797602	-2.212631
38	1	0	-3.284630	2.051601	-1.124895
39	6	0	-3.954597	0.073412	-0.660484
40	6	0	-4.982019	0.518804	0.203424
41	6	0	-5.994868	-0.355957	0.614462
42	1	0	-6.782583	-0.016673	1.277100
43	6	0	-5.996004	-1.679005	0.165888
44	1	0	-6.788411	-2.348048	0.490681
45	6	0	-4.999879	-2.133344	-0.694915
46	1	0	-5.004490	-3.157614	-1.055054
47	6	0	-3.996183	-1.252201	-1.103919
48	1	0	-3.224846	-1.598031	-1.784899
49	6	0	-1.687417	1.224211	0.996965
50	1	0	-2.668695	0.871375	1.312896
51	1	0	-1.650010	2.298904	1.225203
52	6	0	-0.601206	0.514896	1.816293
53	1	0	0.368554	0.976766	1.624035
54	1	0	-0.827217	0.649467	2.888766
55	6	0	-1.640735	-1.700172	1.954773

56	1	0	-1.753142	-1.634420	3.049699
57	1	0	-1.503140	-2.749151	1.678726
58	1	0	-2.558141	-1.346354	1.481071
59	6	0	0.758977	-1.474895	2.072816
60	1	0	0.756863	-1.395730	3.171949
61	1	0	1.622795	-0.940932	1.676038
62	1	0	0.849712	-2.529876	1.800221
63	6	0	-0.760640	-2.601724	-1.880272
64	1	0	-1.445029	-3.253467	-1.316677
65	1	0	-1.315718	-2.305142	-2.784233
66	6	0	0.480522	-3.406850	-2.306332
67	1	0	0.228103	-4.289472	-2.915274
68	1	0	1.047276	-3.771212	-1.439103
69	1	0	1.173012	-2.796084	-2.899144
70	1	0	2.497702	4.974569	0.522603
71	8	0	-4.909960	1.828090	0.597115
72	6	0	-5.947223	2.355914	1.409908
73	1	0	-5.994339	1.850925	2.383543
74	1	0	-5.700333	3.408154	1.561905
75	1	0	-6.923887	2.279222	0.915283

Figure S17 Chemical structure and ball and stick model of $L^{3'}ZnEt$

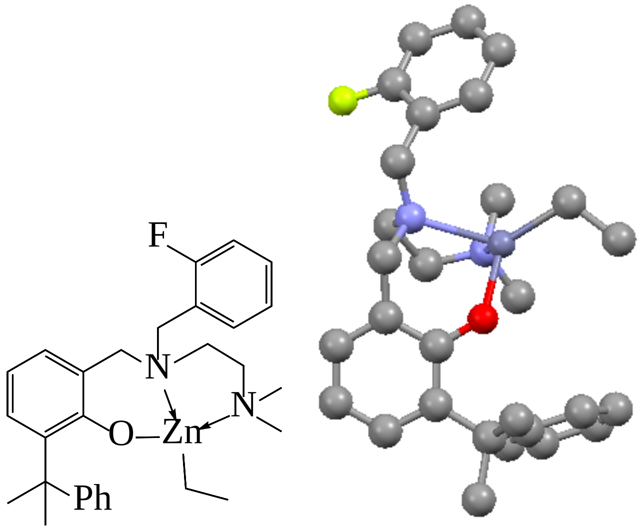

Table S19 Atomic coordinates of $L^{3'}ZnEt$

Standard orientation:

Center Number	Atomic Number	Atomic Type	Coordinates (Angstroms) X	Y	Z
1	30	0	0.592358	-0.709013	0.777454
2	8	0	-1.161749	0.195758	1.033514
3	7	0	1.663876	1.271681	0.260782
4	7	0	0.693395	-0.881627	-1.491440
5	6	0	-1.579797	1.371973	0.614317
6	6	0	-2.973518	1.630199	0.388066
7	6	0	-3.340345	2.870620	-0.141775
8	1	0	-4.387719	3.078079	-0.334575
9	6	0	-2.411383	3.876847	-0.428902
10	6	0	-1.071671	3.656181	-0.132005
11	1	0	-0.339904	4.447804	-0.288331
12	6	0	-0.649363	2.432296	0.398352
13	6	0	-4.028880	0.569449	0.774000
14	6	0	-3.860996	0.213516	2.271009
15	1	0	-2.874690	-0.204085	2.474740
16	1	0	-4.631164	-0.497122	2.594468
17	1	0	-3.979810	1.123668	2.869391

18	6	0	-5.476321	1.106466	0.622974
19	1	0	-6.182873	0.329334	0.934014
20	1	0	-5.724838	1.377839	-0.407529
21	1	0	-5.636794	1.984387	1.259143
22	6	0	-3.923798	-0.663871	-0.148920
23	6	0	-3.772262	-1.971738	0.331004
24	1	0	-3.641006	-2.147192	1.392695
25	6	0	-3.782509	-3.067968	-0.537121
26	1	0	-3.665741	-4.070477	-0.132192
27	6	0	-3.948482	-2.883199	-1.910001
28	1	0	-3.968455	-3.736644	-2.583018
29	6	0	-4.089353	-1.584868	-2.407324
30	1	0	-4.215720	-1.419305	-3.474831
31	6	0	-4.069634	-0.494705	-1.537048
32	1	0	-4.169366	0.509660	-1.940837
33	6	0	0.762616	2.296986	0.904369
34	1	0	0.741062	2.031955	1.970358
35	1	0	1.257376	3.277759	0.822451
36	6	0	3.017533	1.482122	0.866728
37	1	0	2.876432	1.431126	1.951013
38	1	0	3.359974	2.500558	0.633164
39	6	0	4.093603	0.493710	0.475109
40	6	0	5.023639	0.789351	-0.526226
41	6	0	6.056986	-0.067485	-0.885532
42	1	0	6.742002	0.228469	-1.673207
43	6	0	6.182884	-1.282115	-0.210754
44	1	0	6.986352	-1.963259	-0.475910
45	6	0	5.283176	-1.611874	0.805840
46	1	0	5.382848	-2.551178	1.341234
47	6	0	4.257622	-0.728534	1.144474
48	1	0	3.567237	-0.986914	1.940825
49	6	0	1.697601	1.406329	-1.220077
50	1	0	2.696476	1.141768	-1.565940
51	1	0	1.530436	2.451505	-1.516487
52	6	0	0.658074	0.530897	-1.931882
53	1	0	-0.344373	0.911049	-1.729717
54	1	0	0.827729	0.601999	-3.020421
55	6	0	1.907464	-1.576343	-1.953377

56	1	0	1.958048	-1.601343	-3.054395
57	1	0	1.896834	-2.603554	-1.579105
58	1	0	2.804107	-1.086586	-1.569740
59	6	0	-0.504221	-1.596645	-1.973020
60	1	0	-0.550200	-1.608465	-3.073779
61	1	0	-1.402532	-1.118387	-1.581670
62	1	0	-0.477016	-2.629295	-1.614677
63	6	0	1.201175	-2.275487	1.973796
64	1	0	1.980028	-2.865630	1.468757
65	1	0	1.678904	-1.871630	2.880441
66	6	0	0.047307	-3.202866	2.397303
67	1	0	0.377393	-4.018301	3.060176
68	1	0	-0.435252	-3.674248	1.530651
69	1	0	-0.736501	-2.652167	2.932237
70	1	0	-2.741097	4.825335	-0.842968
71	9	0	4.913475	1.967997	-1.190425

Figure S18 Chemical structure and ball and stick model of $L^{4'}$ZnEt

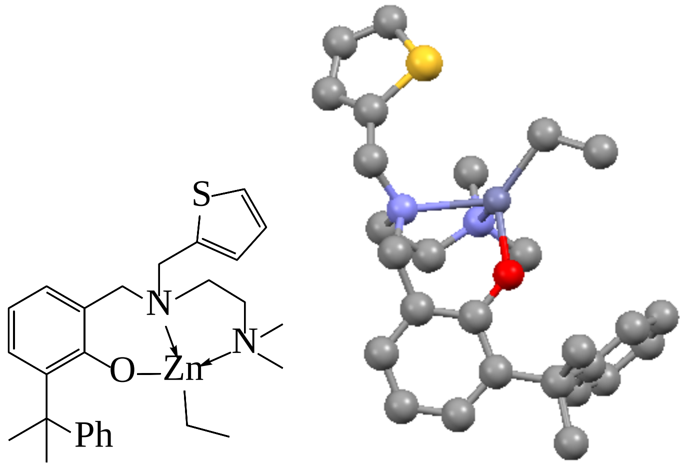

Table S20 Atomic coordinates of $L^{4'}$ZnEt

Standard orientation:

Center	Atomic	Atomic	Coordinates (Angstroms)		
Number	Number	Type	X	Y	Z
1	30	0	0.769768	-0.629126	0.692944
2	8	0	-0.998220	0.241361	0.982751
3	7	0	1.776429	1.367578	0.074746
4	7	0	0.794156	-0.855754	-1.581175
5	6	0	-1.461799	1.389342	0.536583
6	6	0	-2.869095	1.604047	0.353157
7	6	0	-3.287050	2.812339	-0.211269
8	1	0	-4.345861	2.985343	-0.372952
9	6	0	-2.395512	3.829325	-0.570906
10	6	0	-1.041001	3.655180	-0.312320
11	1	0	-0.337005	4.459129	-0.523855
12	6	0	-0.567943	2.464088	0.249207
13	6	0	-3.880408	0.535842	0.825878
14	6	0	-3.645157	0.252641	2.329501
15	1	0	-2.641902	-0.133405	2.511657
16	1	0	-4.384471	-0.459614	2.715351
17	1	0	-3.762090	1.186372	2.890720
18	6	0	-5.346264	1.029804	0.708877
19	1	0	-6.019429	0.250005	1.081264

20	1	0	-5.641584	1.249526	-0.321668
21	1	0	-5.505226	1.930720	1.312482
22	6	0	-3.777220	-0.735750	-0.043639
23	6	0	-3.567283	-2.015175	0.488509
24	1	0	-3.389364	-2.137431	1.550871
25	6	0	-3.577840	-3.150370	-0.327904
26	1	0	-3.414914	-4.129226	0.116993
27	6	0	-3.802428	-3.034496	-1.700091
28	1	0	-3.822049	-3.918574	-2.332353
29	6	0	-4.003182	-1.765485	-2.249385
30	1	0	-4.176734	-1.653493	-3.317242
31	6	0	-3.983156	-0.635866	-1.430778
32	1	0	-4.129714	0.345021	-1.875976
33	6	0	0.862446	2.384118	0.713750
34	1	0	0.881696	2.152570	1.787629
35	1	0	1.323620	3.376881	0.586465
36	6	0	3.138772	1.640805	0.637071
37	1	0	3.036950	1.581805	1.725836
38	1	0	3.418452	2.681396	0.403762
39	6	0	1.768295	1.453931	-1.406942
40	1	0	2.763243	1.178772	-1.762747
41	1	0	1.581092	2.486934	-1.735488
42	6	0	0.720840	0.542565	-2.059967
43	1	0	-0.280350	0.913042	-1.835415
44	1	0	0.853846	0.584576	-3.155161
45	6	0	1.998029	-1.546228	-2.075536
46	1	0	1.998341	-1.606035	-3.176447
47	1	0	2.022551	-2.560643	-1.668580
48	1	0	2.902933	-1.030519	-1.749607
49	6	0	-0.410970	-1.599981	-1.995665
50	1	0	-0.501566	-1.639946	-3.093061
51	1	0	-1.300204	-1.126156	-1.579093
52	1	0	-0.353230	-2.623127	-1.614470
53	6	0	1.475809	-2.098351	1.953250
54	1	0	2.057524	-2.837745	1.384254
55	1	0	2.188071	-1.656322	2.665325
56	6	0	0.355144	-2.806623	2.735163
57	1	0	0.740021	-3.570618	3.429250

58	1	0	-0.352760	-3.313228	2.065763
59	1	0	-0.231890	-2.097672	3.332638
60	1	0	-2.764582	4.751908	-1.009579
61	6	0	4.266022	0.764208	0.172849
62	6	0	5.235911	1.089297	-0.746310
63	16	0	4.572612	-0.822619	0.838299
64	6	0	6.216252	0.069922	-0.932449
65	1	0	5.252467	2.043203	-1.264849
66	6	0	5.987574	-1.022093	-0.140374
67	1	0	7.053013	0.150988	-1.617552
68	1	0	6.565242	-1.934627	-0.073834

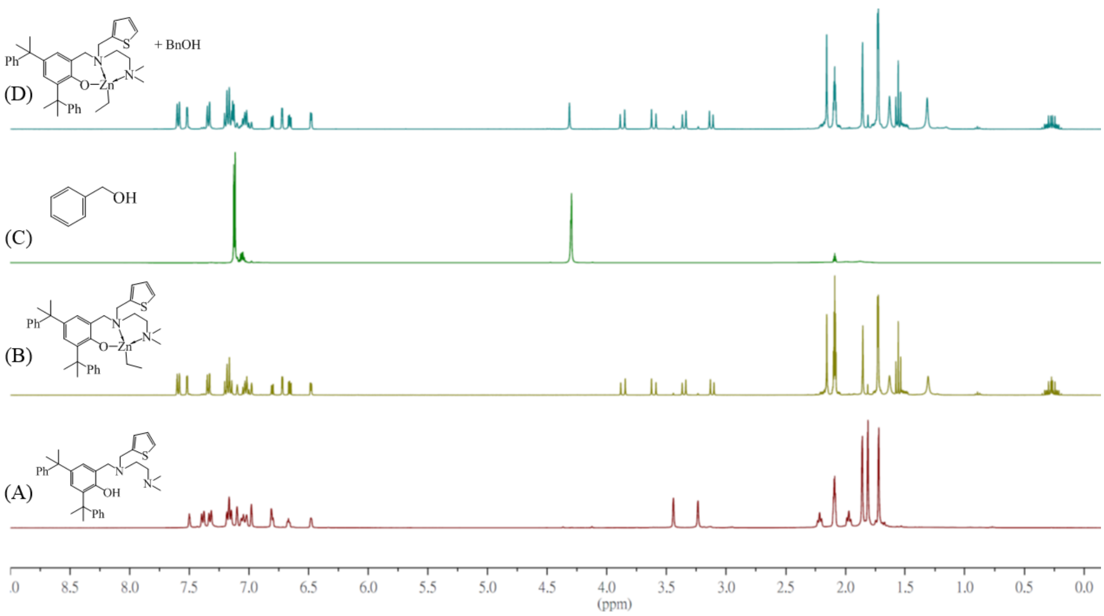

Figure S19. ^{1}H NMR spectra of the reaction of **L^{4}ZnEt** with one equivalent BnOH in d^{8}-toluene.

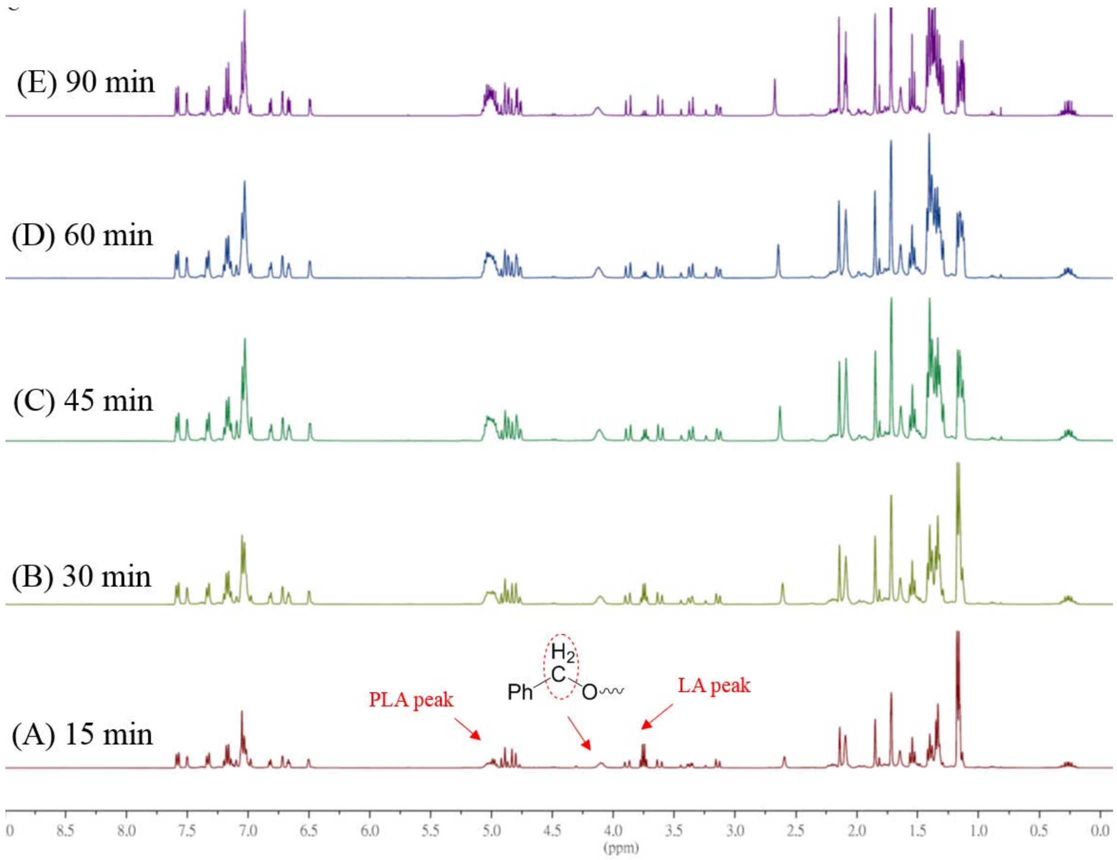

Figure S20. ^{1}H NMR spectra of the LA polymerization ([LA]:[Zn]:[BnOH]=4:1:1, [LA]=0.02 M in d^8-toluene (0.5 mL) at 25 °C).

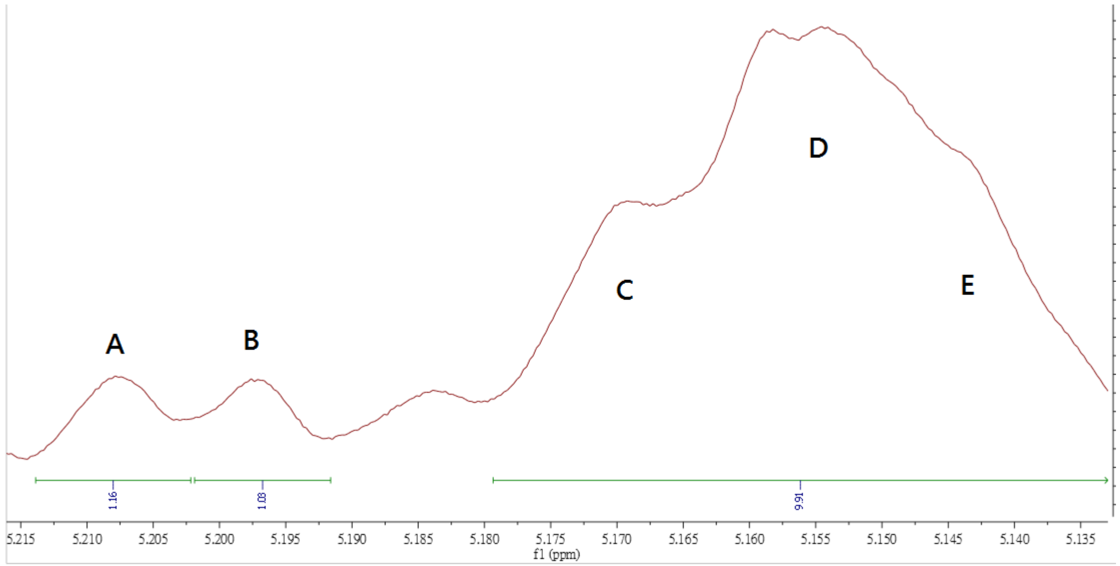

Figure S21. ^{1}H NMR spectra of rac-PLA (entry 9, Table 1) after decoupling at 1.57 ppm. The ratio of the integration between peak A and peak (A+B+C+D+E) is equal to $P_r^2/2$.

Table S21. Crystal data and structure refinement for L^1ZnEt.

Identification code	L^1ZnEt	
Empirical formula	C38 H48 N2 O Zn	
Formula weight	614.15	
Temperature	150(2) K	
Wavelength	0.71073 Å	
Crystal system	Monoclinic	
Space group	P 1 21/c 1	
Unit cell dimensions	a = 12.9647(5) Å	α= 90°.
	b = 20.1011(6) Å	β= 103.824(4)°.
	c = 26.0522(9) Å	γ = 90°.
Volume	6592.7(4) $Å^3$	
Z	8	
Density (calculated)	1.238 Mg/m^3	
Absorption coefficient	0.776 mm^{-1}	
F(000)	2624	
Crystal size	0.4 x 0.2 x 0.2 mm^3	
Theta range for data collection	2.74 to 26.00°.	
Index ranges	-12<=h<=15, -21<=k<=24, -32<=l<=30	
Reflections collected	38523	
Independent reflections	12779 [R(int) = 0.0584]	
Completeness to theta = 26.00°	98.8 %	
Absorption correction	Semi-empirical from equivalents	
Max. and min. transmission	1.00000 and 0.87163	
Refinement method	Full-matrix least-squares on F^2	
Data / restraints / parameters	12779 / 0 / 757	
Goodness-of-fit on F^2	1.015	
Final R indices [I>2sigma(I)]	R1 = 0.0489, wR2 = 0.1049	
R indices (all data)	R1 = 0.0795, wR2 = 0.1193	
Largest diff. peak and hole	0.612 and -0.496 e.$Å^{-3}$	

Atomic coordinates (x 10^4) and equivalent isotropic displacement parameters (Å^{2}x 10^3) for znetonnph. U(eq) is defined as one third of the trace of the orthogonalized U^{ij} tensor.

	x	y	z	U(eq)
Zn(1)	2218(1)	3468(1)	7250(1)	19(1)
Zn(2)	2663(1)	9888(1)	7719(1)	20(1)
O(1)	2025(2)	3873(1)	7904(1)	20(1)
O(2)	2915(2)	9459(1)	7084(1)	21(1)
N(1)	2787(2)	4360(1)	6948(1)	20(1)
N(2)	3852(2)	3210(1)	7456(1)	23(1)
N(3)	1975(2)	9026(1)	7996(1)	21(1)
N(4)	1057(2)	10196(1)	7453(1)	22(1)
C(1)	2457(2)	4429(1)	8129(1)	18(1)
C(2)	2634(2)	4535(1)	8685(1)	17(1)
C(3)	3109(2)	5125(1)	8893(1)	19(1)
C(4)	3426(2)	5631(1)	8594(1)	19(1)
C(5)	3211(2)	5527(1)	8052(1)	20(1)
C(6)	2722(2)	4946(1)	7818(1)	19(1)
C(7)	2240(2)	4023(1)	9036(1)	19(1)
C(8)	1031(2)	3958(2)	8843(1)	25(1)
C(9)	2456(3)	4243(2)	9617(1)	28(1)
C(10)	2797(2)	3345(1)	9032(1)	16(1)
C(11)	3883(2)	3290(2)	9240(1)	24(1)
C(12)	4397(3)	2687(2)	9276(1)	30(1)
C(13)	3830(3)	2114(2)	9097(1)	32(1)
C(14)	2760(3)	2161(2)	8882(1)	28(1)
C(15)	2252(2)	2768(1)	8844(1)	23(1)
C(16)	3904(2)	6275(1)	8868(1)	22(1)
C(17)	4814(2)	6099(2)	9352(1)	31(1)
C(18)	4372(3)	6712(2)	8493(1)	36(1)
C(19)	3052(2)	6689(1)	9040(1)	20(1)
C(20)	1987(3)	6624(2)	8796(1)	30(1)
C(21)	1218(3)	7010(2)	8947(2)	38(1)
C(22)	1506(3)	7467(2)	9345(2)	40(1)
C(23)	2553(3)	7546(2)	9586(1)	42(1)
C(24)	3324(3)	7168(2)	9431(1)	31(1)
C(25)	2367(3)	4914(1)	7224(1)	23(1)

C(26)	2392(3)	4506(2)	6366(1)	30(1)
C(27)	2630(3)	3963(2)	6008(1)	29(1)
C(28)	3508(3)	3990(2)	5796(1)	38(1)
C(29)	3728(3)	3481(2)	5473(1)	48(1)
C(30)	3043(3)	2950(2)	5350(1)	48(1)
C(31)	2150(3)	2925(2)	5542(1)	42(1)
C(32)	1948(3)	3426(2)	5872(1)	34(1)
C(33)	3964(2)	4340(2)	7102(1)	28(1)
C(34)	4372(2)	3871(2)	7551(1)	29(1)
C(35)	4206(3)	2842(2)	7037(1)	35(1)
C(36)	4116(3)	2818(2)	7947(1)	38(1)
C(37)	1140(2)	2786(1)	6944(1)	25(1)
C(38)	786(3)	2410(2)	7387(1)	34(1)
C(39)	2497(2)	8897(1)	6860(1)	19(1)
C(40)	2400(2)	8775(1)	6310(1)	18(1)
C(41)	1932(2)	8186(1)	6094(1)	20(1)
C(42)	1550(2)	7695(1)	6388(1)	20(1)
C(43)	1686(2)	7815(1)	6923(1)	23(1)
C(44)	2160(2)	8393(1)	7165(1)	21(1)
C(45)	2851(2)	9280(1)	5976(1)	19(1)
C(46)	4043(2)	9368(2)	6220(1)	25(1)
C(47)	2764(3)	9024(2)	5408(1)	28(1)
C(48)	2236(2)	9934(1)	5929(1)	17(1)
C(49)	2716(2)	10545(1)	6081(1)	23(1)
C(50)	2147(3)	11134(2)	5990(1)	30(1)
C(51)	1088(3)	11129(2)	5751(1)	33(1)
C(52)	579(3)	10531(2)	5608(1)	31(1)
C(53)	1155(2)	9942(2)	5699(1)	24(1)
C(54)	1107(2)	7044(1)	6116(1)	22(1)
C(55)	216(2)	7203(2)	5623(1)	29(1)
C(56)	643(3)	6596(2)	6482(1)	33(1)
C(57)	2020(2)	6651(1)	5971(1)	20(1)
C(58)	3031(3)	6681(2)	6296(1)	31(1)
C(59)	3864(3)	6327(2)	6183(1)	35(1)
C(60)	3701(3)	5930(2)	5739(1)	31(1)
C(61)	2706(3)	5885(2)	5418(1)	31(1)
C(62)	1874(3)	6245(2)	5532(1)	25(1)
C(63)	2430(3)	8446(1)	7754(1)	24(1)

C(64)	2247(3)	8889(2)	8579(1)	30(1)
C(65)	1937(3)	9435(2)	8912(1)	27(1)
C(66)	1013(3)	9394(2)	9088(1)	36(1)
C(67)	720(3)	9892(2)	9388(1)	49(1)
C(68)	1376(4)	10437(2)	9527(2)	57(1)
C(69)	2306(4)	10483(2)	9364(1)	55(1)
C(70)	2588(3)	9981(2)	9056(1)	41(1)
C(71)	809(2)	9070(2)	7790(1)	27(1)
C(72)	498(2)	9550(2)	7330(1)	26(1)
C(73)	643(3)	10577(2)	7844(1)	31(1)
C(74)	915(3)	10589(2)	6962(1)	34(1)
C(75)	3758(2)	10559(2)	8022(1)	27(1)
C(76)	4135(3)	10892(2)	7571(1)	36(1)

Bond lengths [Å] and angles [°] for znetonnph.

Zn(1)-O(1)	1.9583(18)	C(8)-H(8A)	0.9600	C(25)-H(25B)	0.9700
Zn(1)-C(37)	1.983(3)	C(8)-H(8B)	0.9600	C(26)-C(27)	1.514(4)
Zn(1)-N(2)	2.121(2)	C(8)-H(8C)	0.9600	C(26)-H(26A)	0.9700
Zn(1)-N(1)	2.159(2)	C(9)-H(9A)	0.9600	C(26)-H(26B)	0.9700
Zn(2)-O(2)	1.9602(19)	C(9)-H(9B)	0.9600	C(27)-C(28)	1.380(4)
Zn(2)-C(75)	1.981(3)	C(9)-H(9C)	0.9600	C(27)-C(32)	1.387(4)
Zn(2)-N(4)	2.123(2)	C(10)-C(15)	1.386(4)	C(28)-C(29)	1.397(5)
Zn(2)-N(3)	2.151(2)	C(10)-C(11)	1.388(4)	C(28)-H(28A)	0.9300
O(1)-C(1)	1.321(3)	C(11)-C(12)	1.374(4)	C(29)-C(30)	1.377(5)
O(2)-C(39)	1.326(3)	C(11)-H(11A)	0.9300	C(29)-H(29A)	0.9300
N(1)-C(33)	1.482(4)	C(12)-C(13)	1.386(4)	C(30)-C(31)	1.369(5)
N(1)-C(25)	1.495(3)	C(12)-H(12A)	0.9300	C(30)-H(30A)	0.9300
N(1)-C(26)	1.509(4)	C(13)-C(14)	1.369(4)	C(31)-C(32)	1.389(4)
N(2)-C(35)	1.478(4)	C(13)-H(13A)	0.9300	C(31)-H(31A)	0.9300
N(2)-C(36)	1.472(4)	C(14)-C(15)	1.379(4)	C(32)-H(32A)	0.9300
N(2)-C(34)	1.484(4)	C(14)-H(14A)	0.9300	C(33)-C(34)	1.497(4)
N(3)-C(71)	1.482(4)	C(15)-H(15A)	0.9300	C(33)-H(33A)	0.9700
N(3)-C(64)	1.499(4)	C(16)-C(19)	1.534(4)	C(33)-H(33B)	0.9700
N(3)-C(63)	1.509(3)	C(16)-C(18)	1.540(4)	C(34)-H(34A)	0.9700
N(4)-C(74)	1.477(4)	C(16)-C(17)	1.549(4)	C(34)-H(34B)	0.9700
N(4)-C(73)	1.475(4)	C(17)-H(17A)	0.9600	C(35)-H(35A)	0.9600
N(4)-C(72)	1.483(4)	C(17)-H(17B)	0.9600	C(35)-H(35B)	0.9600
C(1)-C(6)	1.409(4)	C(17)-H(17C)	0.9600	C(35)-H(35C)	0.9600
C(1)-C(2)	1.428(4)	C(18)-H(18A)	0.9600	C(36)-H(36A)	0.9600
C(2)-C(3)	1.385(4)	C(18)-H(18B)	0.9600	C(36)-H(36B)	0.9600
C(2)-C(7)	1.542(4)	C(18)-H(18C)	0.9600	C(36)-H(36C)	0.9600
C(3)-C(4)	1.401(4)	C(19)-C(20)	1.381(4)	C(37)-C(38)	1.540(4)
C(3)-H(3A)	0.9300	C(19)-C(24)	1.385(4)	C(37)-H(37A)	0.9700
C(4)-C(5)	1.389(4)	C(20)-C(21)	1.393(4)	C(37)-H(37B)	0.9700
C(4)-C(16)	1.534(4)	C(20)-H(20A)	0.9300	C(38)-H(38A)	0.9600
C(5)-C(6)	1.399(4)	C(21)-C(22)	1.368(5)	C(38)-H(38B)	0.9600
C(5)-H(5A)	0.9300	C(21)-H(21A)	0.9300	C(38)-H(38C)	0.9600
C(6)-C(25)	1.508(4)	C(22)-C(23)	1.363(5)	C(39)-C(44)	1.420(4)
C(7)-C(8)	1.533(4)	C(22)-H(22A)	0.9300	C(39)-C(40)	1.430(4)
C(7)-C(9)	1.537(4)	C(23)-C(24)	1.390(4)	C(40)-C(41)	1.387(4)

C(7)-C(10)	1.543(4)	C(23)-H(23A)	0.9300	C(57)-C(62)	1.382(4)
C(25)-H(25A)	0.9700	C(24)-H(24A)	0.9300	C(57)-C(58)	1.381(4)
C(41)-H(41A)	0.9300	C(58)-C(59)	1.382(4)	C(74)-H(74C)	0.9600
C(42)-C(43)	1.385(4)	C(58)-H(58A)	0.9300	C(75)-C(76)	1.530(4)
C(42)-C(54)	1.534(4)	C(59)-C(60)	1.379(4)	C(40)-C(45)	1.542(4)
C(43)-C(44)	1.392(4)	C(59)-H(59A)	0.9300	C(41)-C(42)	1.409(4)
C(43)-H(43A)	0.9300	C(60)-C(61)	1.362(4)	C(75)-H(75A)	0.9700
C(44)-C(63)	1.496(4)	C(60)-H(60A)	0.9300	C(75)-H(75B)	0.9700
C(45)-C(48)	1.527(4)	C(61)-C(62)	1.387(4)	C(76)-H(76A)	0.9600
C(45)-C(46)	1.534(4)	C(61)-H(61A)	0.9300	C(76)-H(76B)	0.9600
C(45)-C(47)	1.544(4)	C(62)-H(62A)	0.9300	C(76)-H(76C)	0.9600
C(46)-H(46A)	0.9600	C(63)-H(63A)	0.9700		
C(46)-H(46B)	0.9600	C(63)-H(63B)	0.9700		
C(46)-H(46C)	0.9600	C(64)-C(65)	1.512(4)		
C(47)-H(47A)	0.9600	C(64)-H(64A)	0.9700		
C(47)-H(47B)	0.9600	C(64)-H(64B)	0.9700		
C(47)-H(47C)	0.9600	C(65)-C(70)	1.381(4)		
C(48)-C(53)	1.387(4)	C(65)-C(66)	1.385(4)		
C(48)-C(49)	1.392(4)	C(66)-C(67)	1.376(5)		
C(49)-C(50)	1.384(4)	C(66)-H(66A)	0.9300		
C(49)-H(49A)	0.9300	C(67)-C(68)	1.382(6)		
C(50)-C(51)	1.366(4)	C(67)-H(67A)	0.9300		
C(50)-H(50A)	0.9300	C(68)-C(69)	1.374(6)		
C(51)-C(52)	1.379(4)	C(68)-H(68A)	0.9300		
C(51)-H(51A)	0.9300	C(69)-C(70)	1.389(5)		
C(52)-C(53)	1.390(4)	C(69)-H(69A)	0.9300		
C(52)-H(52A)	0.9300	C(70)-H(70A)	0.9300		
C(53)-H(53A)	0.9300	C(71)-C(72)	1.516(4)		
C(54)-C(56)	1.535(4)	C(71)-H(71A)	0.9700		
C(54)-C(55)	1.543(4)	C(71)-H(71B)	0.9700		
C(54)-C(57)	1.542(4)	C(72)-H(72A)	0.9700		
C(55)-H(55A)	0.9600	C(72)-H(72B)	0.9700		
C(55)-H(55B)	0.9600	C(73)-H(73A)	0.9600		
C(55)-H(55C)	0.9600	C(73)-H(73B)	0.9600		
C(56)-H(56A)	0.9600	C(73)-H(73C)	0.9600		
C(56)-H(56B)	0.9600	C(74)-H(74A)	0.9600		
C(56)-H(56C)	0.9600	C(74)-H(74B)	0.9600		

O(1)-Zn(1)-C(37)	114.38(10)	C(72)-N(4)-Zn(2)	101.88(17)	C(7)-C(9)-H(9B)	109.5
O(1)-Zn(1)-N(2)	102.08(9)	O(1)-C(1)-C(6)	120.3(3)	H(9A)-C(9)-H(9B)	109.5
C(37)-Zn(1)-N(2)	119.88(11)	O(1)-C(1)-C(2)	121.6(2)	C(7)-C(9)-H(9C)	109.5
O(1)-Zn(1)-N(1)	95.61(8)	C(6)-C(1)-C(2)	118.0(3)	H(9A)-C(9)-H(9C)	109.5
C(37)-Zn(1)-N(1)	134.15(11)	C(3)-C(2)-C(1)	118.3(2)	H(9B)-C(9)-H(9C)	109.5
N(2)-Zn(1)-N(1)	83.55(9)	C(3)-C(2)-C(7)	121.2(2)	C(15)-C(10)-C(11)	117.0(3)
O(2)-Zn(2)-C(75)	112.87(10)	C(1)-C(2)-C(7)	120.4(2)	C(15)-C(10)-C(7)	122.9(3)
O(2)-Zn(2)-N(4)	101.62(9)	C(2)-C(3)-C(4)	124.5(3)	C(11)-C(10)-C(7)	120.1(3)
C(75)-Zn(2)-N(4)	118.95(11)	C(2)-C(3)-H(3A)	117.8	C(12)-C(11)-C(10)	121.9(3)
O(2)-Zn(2)-N(3)	95.49(8)	C(4)-C(3)-H(3A)	117.8	C(12)-C(11)-H(11A)	119.1
C(75)-Zn(2)-N(3)	136.85(11)	C(5)-C(4)-C(3)	116.2(3)	C(10)-C(11)-H(11A)	119.1
N(4)-Zn(2)-N(3)	83.76(9)	C(5)-C(4)-C(16)	124.0(3)	C(11)-C(12)-C(13)	120.0(3)
C(1)-O(1)-Zn(1)	127.44(17)	C(3)-C(4)-C(16)	119.6(3)	C(11)-C(12)-H(12A)	120.0
C(39)-O(2)-Zn(2)	127.71(17)	C(4)-C(5)-C(6)	121.9(3)	C(13)-C(12)-H(12A)	120.0
C(33)-N(1)-C(25)	111.1(2)	C(4)-C(5)-H(5A)	119.0	C(14)-C(13)-C(12)	119.0(3)
C(33)-N(1)-C(26)	111.0(2)	C(6)-C(5)-H(5A)	119.0	C(14)-C(13)-H(13A)	120.5
C(25)-N(1)-C(26)	105.3(2)	C(5)-C(6)-C(1)	120.9(3)	C(12)-C(13)-H(13A)	120.5
C(33)-N(1)-Zn(1)	107.30(17)	C(5)-C(6)-C(25)	118.6(2)	C(13)-C(14)-C(15)	120.7(3)
C(25)-N(1)-Zn(1)	104.34(16)	C(1)-C(6)-C(25)	120.1(3)	C(13)-C(14)-H(14A)	119.7
C(26)-N(1)-Zn(1)	117.65(18)	C(8)-C(7)-C(9)	106.2(2)	C(15)-C(14)-H(14A)	119.7
C(35)-N(2)-C(36)	109.0(2)	C(72)-N(4)-Zn(2)	101.88(17)	C(10)-C(15)-C(14)	121.4(3)
C(35)-N(2)-C(34)	111.2(2)	O(1)-C(1)-C(6)	120.3(3)	C(10)-C(15)-H(15A)	119.3
C(36)-N(2)-C(34)	109.4(3)	O(1)-C(1)-C(2)	121.6(2)	C(14)-C(15)-H(15A)	119.3
C(35)-N(2)-Zn(1)	113.73(19)	C(6)-C(1)-C(2)	118.0(3)	C(4)-C(16)-C(19)	110.7(2)
C(36)-N(2)-Zn(1)	111.14(19)	C(3)-C(2)-C(1)	118.3(2)	C(4)-C(16)-C(18)	111.3(2)
C(34)-N(2)-Zn(1)	102.10(17)	C(8)-C(7)-C(2)	108.6(2)	C(19)-C(16)-C(18)	107.2(2)
C(71)-N(3)-C(64)	110.5(2)	C(9)-C(7)-C(2)	112.6(2)	C(4)-C(16)-C(17)	109.3(2)
C(71)-N(3)-C(63)	111.2(2)	C(8)-C(7)-C(10)	111.4(2)	C(19)-C(16)-C(17)	110.4(2)
C(64)-N(3)-C(63)	105.1(2)	C(9)-C(7)-C(10)	106.6(2)	C(18)-C(16)-C(17)	107.9(3)
C(71)-N(3)-Zn(2)	107.38(17)	C(2)-C(7)-C(10)	111.3(2)	C(16)-C(17)-H(17A)	109.5
C(64)-N(3)-Zn(2)	118.15(18)	C(7)-C(8)-H(8A)	109.5	C(16)-C(17)-H(17B)	109.5
C(63)-N(3)-Zn(2)	104.31(16)	C(7)-C(8)-H(8B)	109.5	H(17A)-C(17)-H(17B)	109.5
C(74)-N(4)-C(73)	109.2(2)	H(8A)-C(8)-H(8B)	109.5	C(16)-C(17)-H(17C)	109.5
C(74)-N(4)-C(72)	109.1(2)	C(7)-C(8)-H(8C)	109.5	H(17A)-C(17)-H(17C)	109.5
C(73)-N(4)-C(72)	111.4(2)	H(8A)-C(8)-H(8C)	109.5	H(17B)-C(17)-H(17C)	109.5
C(74)-N(4)-Zn(2)	110.34(18)	H(8B)-C(8)-H(8C)	109.5	C(16)-C(18)-H(18A)	109.5
C(73)-N(4)-Zn(2)	114.66(19)	C(7)-C(9)-H(9A)	109.5	C(16)-C(18)-H(18B)	109.5

H(18A)-C(18)-H(18B)	109.5	C(27)-C(28)-C(29)	121.3(3)	H(36A)-C(36)-H(36C)	109.5
C(16)-C(18)-H(18C)	109.5	C(27)-C(28)-H(28A)	119.4	H(36B)-C(36)-H(36C)	109.5
H(18A)-C(18)-H(18C)	109.5	C(29)-C(28)-H(28A)	119.4	C(38)-C(37)-Zn(1)	110.3(2)
H(18B)-C(18)-H(18C)	109.5	C(30)-C(29)-C(28)	119.7(4)	C(38)-C(37)-H(37A)	109.6
C(20)-C(19)-C(24)	117.1(3)	C(30)-C(29)-H(29A)	120.1	Zn(1)-C(37)-H(37A)	109.6
C(20)-C(19)-C(16)	121.6(3)	C(28)-C(29)-H(29A)	120.1	C(38)-C(37)-H(37B)	109.6
C(24)-C(19)-C(16)	121.2(3)	C(31)-C(30)-C(29)	119.8(3)	Zn(1)-C(37)-H(37B)	109.6
C(19)-C(20)-C(21)	121.4(3)	C(31)-C(30)-H(30A)	120.1	H(37A)-C(37)-H(37B)	108.1
C(19)-C(20)-H(20A)	119.3	C(29)-C(30)-H(30A)	120.1	C(37)-C(38)-H(38A)	109.5
C(21)-C(20)-H(20A)	119.3	C(30)-C(31)-C(32)	120.1(4)	C(37)-C(38)-H(38B)	109.5
C(22)-C(21)-C(20)	120.3(3)	C(30)-C(31)-H(31A)	119.9	H(38A)-C(38)-H(38B)	109.5
C(22)-C(21)-H(21A)	119.8	C(32)-C(31)-H(31A)	119.9	C(37)-C(38)-H(38C)	109.5
C(20)-C(21)-H(21A)	119.8	C(31)-C(32)-C(27)	121.3(3)	H(38A)-C(38)-H(38C)	109.5
C(21)-C(22)-C(23)	119.3(3)	C(31)-C(32)-H(32A)	119.4	H(38B)-C(38)-H(38C)	109.5
C(21)-C(22)-H(22A)	120.4	C(27)-C(32)-H(32A)	119.4	O(2)-C(39)-C(44)	120.8(3)
C(23)-C(22)-H(22A)	120.4	N(1)-C(33)-C(34)	112.2(2)	O(2)-C(39)-C(40)	121.0(2)
C(22)-C(23)-C(24)	120.6(3)	N(1)-C(33)-H(33A)	109.2	C(44)-C(39)-C(40)	118.2(3)
C(22)-C(23)-H(23A)	119.7	C(34)-C(33)-H(33A)	109.2	C(41)-C(40)-C(39)	118.6(2)
C(24)-C(23)-H(23A)	119.7	N(1)-C(33)-H(33B)	109.2	C(41)-C(40)-C(45)	121.4(2)
C(19)-C(24)-C(23)	121.3(3)	C(34)-C(33)-H(33B)	109.2	C(39)-C(40)-C(45)	120.0(2)
C(19)-C(24)-H(24A)	119.3	H(33A)-C(33)-H(33B)	107.9	C(40)-C(41)-C(42)	123.6(3)
C(23)-C(24)-H(24A)	119.3	N(2)-C(34)-C(33)	111.8(3)	C(40)-C(41)-H(41A)	118.2
N(1)-C(25)-C(6)	117.9(2)	N(2)-C(34)-H(34A)	109.3	C(42)-C(41)-H(41A)	118.2
N(1)-C(25)-H(25A)	107.8	C(33)-C(34)-H(34A)	109.3	C(43)-C(42)-C(41)	116.7(3)
C(6)-C(25)-H(25A)	107.8	N(2)-C(34)-H(34B)	109.3	C(43)-C(42)-C(54)	123.8(3)
N(1)-C(25)-H(25B)	107.8	C(33)-C(34)-H(34B)	109.3	C(41)-C(42)-C(54)	119.3(3)
C(6)-C(25)-H(25B)	107.8	H(34A)-C(34)-H(34B)	107.9	C(42)-C(43)-C(44)	122.4(3)
H(25A)-C(25)-H(25B)	107.2	N(2)-C(35)-H(35A)	109.5	C(42)-C(43)-H(43A)	118.8
N(1)-C(26)-C(27)	114.1(2)	N(2)-C(35)-H(35B)	109.5	C(44)-C(43)-H(43A)	118.8
N(1)-C(26)-H(26A)	108.7	H(35A)-C(35)-H(35B)	109.5	H(36A)-C(36)-H(36C)	109.5
C(27)-C(26)-H(26A)	108.7	N(2)-C(35)-H(35C)	109.5	H(36B)-C(36)-H(36C)	109.5
N(1)-C(26)-H(26B)	108.7	H(35A)-C(35)-H(35C)	109.5	C(38)-C(37)-Zn(1)	110.3(2)
C(27)-C(26)-H(26B)	108.7	H(35B)-C(35)-H(35C)	109.5	C(38)-C(37)-H(37A)	109.6
H(26A)-C(26)-H(26B)	107.6	N(2)-C(36)-H(36A)	109.5	Zn(1)-C(37)-H(37A)	109.6
C(28)-C(27)-C(32)	117.7(3)	N(2)-C(36)-H(36B)	109.5	C(38)-C(37)-H(37B)	109.6
C(28)-C(27)-C(26)	121.8(3)	H(36A)-C(36)-H(36B)	109.5	Zn(1)-C(37)-H(37B)	109.6
C(32)-C(27)-C(26)	120.4(3)	N(2)-C(36)-H(36C)	109.5	H(37A)-C(37)-H(37B)	108.1

C(43)-C(44)-C(39)	120.3(3)	C(52)-C(53)-H(53A)	119.0	C(61)-C(62)-H(62A)	119.2
C(43)-C(44)-C(63)	119.6(3)	C(48)-C(53)-H(53A)	119.0	C(44)-C(63)-N(3)	117.7(2)
C(39)-C(44)-C(63)	119.7(3)	C(42)-C(54)-C(56)	112.0(2)	C(44)-C(63)-H(63A)	107.9
C(48)-C(45)-C(46)	112.9(2)	C(42)-C(54)-C(55)	109.3(2)	N(3)-C(63)-H(63A)	107.9
C(48)-C(45)-C(47)	107.1(2)	C(56)-C(54)-C(55)	108.2(2)	C(44)-C(63)-H(63B)	107.9
C(46)-C(45)-C(47)	105.9(2)	C(42)-C(54)-C(57)	109.0(2)	N(3)-C(63)-H(63B)	107.9
C(48)-C(45)-C(40)	110.4(2)	C(56)-C(54)-C(57)	106.9(2)	H(63A)-C(63)-H(63B)	107.2
C(46)-C(45)-C(40)	108.8(2)	C(55)-C(54)-C(57)	111.6(2)	N(3)-C(64)-C(65)	114.7(2)
C(47)-C(45)-C(40)	111.8(2)	C(54)-C(55)-H(55A)	109.5	N(3)-C(64)-H(64A)	108.6
C(45)-C(46)-H(46A)	109.5	C(54)-C(55)-H(55B)	109.5	C(65)-C(64)-H(64A)	108.6
C(45)-C(46)-H(46B)	109.5	H(55A)-C(55)-H(55B)	109.5	N(3)-C(64)-H(64B)	108.6
H(46A)-C(46)-H(46B)	109.5	C(54)-C(55)-H(55C)	109.5	C(65)-C(64)-H(64B)	108.6
C(45)-C(46)-H(46C)	109.5	H(55A)-C(55)-H(55C)	109.5	H(64A)-C(64)-H(64B)	107.6
H(46A)-C(46)-H(46C)	109.5	H(55B)-C(55)-H(55C)	109.5	C(70)-C(65)-C(66)	118.5(3)
H(46B)-C(46)-H(46C)	109.5	C(54)-C(56)-H(56A)	109.5	C(70)-C(65)-C(64)	120.3(3)
C(45)-C(47)-H(47A)	109.5	C(54)-C(56)-H(56B)	109.5	C(66)-C(65)-C(64)	121.2(3)
C(45)-C(47)-H(47B)	109.5	H(56A)-C(56)-H(56B)	109.5	C(67)-C(66)-C(65)	121.6(4)
H(47A)-C(47)-H(47B)	109.5	C(54)-C(56)-H(56C)	109.5	C(67)-C(66)-H(66A)	119.2
C(45)-C(47)-H(47C)	109.5	H(56A)-C(56)-H(56C)	109.5	C(65)-C(66)-H(66A)	119.2
H(47A)-C(47)-H(47C)	109.5	H(56B)-C(56)-H(56C)	109.5	C(66)-C(67)-C(68)	119.2(4)
H(47B)-C(47)-H(47C)	109.5	C(62)-C(57)-C(58)	117.1(3)	C(66)-C(67)-H(67A)	120.4
C(53)-C(48)-C(49)	116.8(3)	C(62)-C(57)-C(54)	122.9(3)	C(68)-C(67)-H(67A)	120.4
C(53)-C(48)-C(45)	119.9(3)	C(58)-C(57)-C(54)	120.0(3)	C(69)-C(68)-C(67)	120.2(4)
C(49)-C(48)-C(45)	123.2(3)	C(57)-C(58)-C(59)	121.5(3)	C(69)-C(68)-H(68A)	119.9
C(50)-C(49)-C(48)	121.5(3)	C(57)-C(58)-H(58A)	119.2	C(67)-C(68)-H(68A)	119.9
C(50)-C(49)-H(49A)	119.3	C(59)-C(58)-H(58A)	119.2	C(68)-C(69)-C(70)	120.1(4)
C(48)-C(49)-H(49A)	119.3	C(60)-C(59)-C(58)	120.4(3)	C(68)-C(69)-H(69A)	120.0
C(51)-C(50)-C(49)	120.6(3)	C(60)-C(59)-H(59A)	119.8	C(70)-C(69)-H(69A)	120.0
C(51)-C(50)-H(50A)	119.7	C(58)-C(59)-H(59A)	119.8	C(65)-C(70)-C(69)	120.4(4)
C(49)-C(50)-H(50A)	119.7	C(61)-C(60)-C(59)	119.0(3)	C(65)-C(70)-H(70A)	119.8
C(50)-C(51)-C(52)	119.5(3)	C(61)-C(60)-H(60A)	120.5	C(69)-C(70)-H(70A)	119.8
C(50)-C(51)-H(51A)	120.2	C(59)-C(60)-H(60A)	120.5	N(3)-C(71)-C(72)	112.3(2)
C(52)-C(51)-H(51A)	120.2	C(60)-C(61)-C(62)	120.4(3)	C(61)-C(62)-H(62A)	119.2
C(51)-C(52)-C(53)	119.7(3)	C(60)-C(61)-H(61A)	119.8	C(44)-C(63)-N(3)	117.7(2)
C(51)-C(52)-H(52A)	120.2	C(62)-C(61)-H(61A)	119.8	C(44)-C(63)-H(63A)	107.9
C(53)-C(52)-H(52A)	120.2	C(57)-C(62)-C(61)	121.6(3)	N(3)-C(63)-H(63A)	107.9
C(52)-C(53)-C(48)	121.9(3)	C(57)-C(62)-H(62A)	119.2	C(44)-C(63)-H(63B)	107.9

N(3)-C(71)-H(71A)	109.1	H(74A)-C(74)-H(74C)	109.5		
C(72)-C(71)-H(71A)	109.1	H(74B)-C(74)-H(74C)	109.5		
N(3)-C(71)-H(71B)	109.1	C(76)-C(75)-Zn(2)	108.7(2)		
C(72)-C(71)-H(71B)	109.1	C(76)-C(75)-H(75A)	109.9		
H(71A)-C(71)-H(71B)	107.9	Zn(2)-C(75)-H(75A)	109.9		
N(4)-C(72)-C(71)	111.3(2)	C(76)-C(75)-H(75B)	109.9		
N(4)-C(72)-H(72A)	109.4	Zn(2)-C(75)-H(75B)	109.9		
C(71)-C(72)-H(72A)	109.4	H(75A)-C(75)-H(75B)	108.3		
N(4)-C(72)-H(72B)	109.4	C(75)-C(76)-H(76A)	109.5		
C(71)-C(72)-H(72B)	109.4	C(75)-C(76)-H(76B)	109.5		
H(72A)-C(72)-H(72B)	108.0	H(76A)-C(76)-H(76B)	109.5		
N(4)-C(73)-H(73A)	109.5	C(75)-C(76)-H(76C)	109.5		
N(4)-C(73)-H(73B)	109.5	H(76A)-C(76)-H(76C)	109.5		
N(3)-C(71)-H(71A)	109.1	H(76B)-C(76)-H(76C)	109.5		
C(72)-C(71)-H(71A)	109.1	C(76)-C(75)-H(75B)	109.9		
N(3)-C(71)-H(71B)	109.1				
C(72)-C(71)-H(71B)	109.1				
H(71A)-C(71)-H(71B)	107.9				
N(4)-C(72)-C(71)	111.3(2)				
H(73A)-C(73)-H(73B)	109.5				
N(4)-C(73)-H(73C)	109.5				
H(73A)-C(73)-H(73C)	109.5				
H(73B)-C(73)-H(73C)	109.5				
N(4)-C(74)-H(74A)	109.5				
N(4)-C(74)-H(74B)	109.5				
H(74A)-C(74)-H(74B)	109.5				
N(4)-C(74)-H(74C)	109.5				

Symmetry transformations used to generate equivalent atoms:

Anisotropic displacement parameters ($Å^2$x 10^3) for znetonnph. The anisotropic displacement factor exponent takes the form: $-2\pi^2$[h^2 $a^{*2}U^{11}$ + ... + 2 h k a* b* U^{12}]

	U^{11}	U^{22}	U^{33}	U^{23}	U^{13}	U^{12}
Zn(1)	23(1)	17(1)	18(1)	-3(1)	6(1)	-3(1)
Zn(2)	23(1)	18(1)	19(1)	-3(1)	6(1)	-4(1)
O(1)	26(1)	16(1)	19(1)	-4(1)	10(1)	-5(1)
O(2)	27(1)	18(1)	20(1)	-3(1)	10(1)	-3(1)
N(1)	29(2)	18(1)	13(1)	0(1)	6(1)	-1(1)
N(2)	28(2)	24(1)	18(1)	-3(1)	6(1)	4(1)
N(3)	32(2)	19(1)	15(1)	-2(1)	10(1)	-3(1)
N(4)	25(2)	22(1)	20(1)	-1(1)	9(1)	-1(1)
C(1)	18(2)	16(2)	19(2)	-2(1)	5(1)	-1(1)
C(2)	18(2)	16(2)	18(2)	-2(1)	7(1)	2(1)
C(3)	24(2)	21(2)	15(2)	-2(1)	9(1)	1(1)
C(4)	20(2)	16(2)	23(2)	-6(1)	10(1)	-1(1)
C(5)	28(2)	16(2)	21(2)	0(1)	12(1)	0(1)
C(6)	26(2)	16(2)	16(2)	-3(1)	9(1)	1(1)
C(7)	22(2)	18(2)	19(2)	0(1)	10(1)	-2(1)
C(8)	24(2)	23(2)	32(2)	3(2)	13(2)	2(1)
C(9)	40(2)	26(2)	21(2)	-3(1)	15(2)	-2(2)
C(10)	24(2)	17(2)	11(2)	3(1)	10(1)	-1(1)
C(11)	25(2)	28(2)	18(2)	-1(1)	6(1)	-3(1)
C(12)	24(2)	44(2)	21(2)	7(2)	4(1)	8(2)
C(13)	47(2)	26(2)	26(2)	8(2)	17(2)	12(2)
C(14)	39(2)	18(2)	30(2)	3(1)	12(2)	-1(2)
C(15)	26(2)	22(2)	22(2)	2(1)	8(1)	-2(1)
C(16)	25(2)	20(2)	25(2)	-4(1)	11(1)	-5(1)
C(17)	23(2)	28(2)	43(2)	-14(2)	7(2)	-1(2)
C(18)	46(2)	28(2)	42(2)	-13(2)	28(2)	-19(2)
C(19)	26(2)	14(2)	22(2)	2(1)	11(1)	-2(1)
C(20)	34(2)	24(2)	36(2)	-7(2)	15(2)	-4(2)
C(21)	33(2)	35(2)	50(2)	10(2)	18(2)	5(2)
C(22)	54(3)	33(2)	42(2)	18(2)	29(2)	27(2)
C(23)	74(3)	27(2)	27(2)	-2(2)	15(2)	17(2)
C(24)	38(2)	24(2)	28(2)	-4(2)	5(2)	4(2)
C(25)	39(2)	14(2)	17(2)	2(1)	10(2)	2(1)

C(26)	51(2)	24(2)	16(2)	-2(1)	8(2)	2(2)
C(27)	52(2)	21(2)	13(2)	4(1)	6(2)	-3(2)
C(28)	67(3)	31(2)	22(2)	3(2)	20(2)	-4(2)
C(29)	74(3)	48(3)	29(2)	2(2)	27(2)	9(2)
C(30)	90(3)	33(2)	24(2)	-5(2)	19(2)	11(2)
C(31)	75(3)	24(2)	23(2)	0(2)	3(2)	-4(2)
C(32)	51(2)	36(2)	16(2)	2(2)	6(2)	3(2)
C(33)	32(2)	30(2)	27(2)	-8(2)	17(2)	-10(2)
C(34)	21(2)	40(2)	27(2)	-13(2)	9(2)	-3(2)
C(35)	40(2)	39(2)	29(2)	-4(2)	15(2)	10(2)
C(36)	46(2)	48(2)	22(2)	9(2)	10(2)	20(2)
C(37)	26(2)	23(2)	25(2)	-8(1)	6(1)	-7(1)
C(38)	37(2)	25(2)	41(2)	-1(2)	9(2)	-9(2)
C(39)	20(2)	16(2)	21(2)	1(1)	7(1)	2(1)
C(40)	23(2)	14(2)	20(2)	2(1)	12(1)	2(1)
C(41)	25(2)	22(2)	16(2)	-1(1)	9(1)	2(1)
C(42)	23(2)	15(2)	23(2)	-2(1)	9(1)	0(1)
C(43)	34(2)	16(2)	25(2)	6(1)	18(2)	3(1)
C(44)	30(2)	17(2)	18(2)	-3(1)	10(1)	1(1)
C(45)	24(2)	16(2)	20(2)	0(1)	9(1)	1(1)
C(46)	23(2)	26(2)	29(2)	7(2)	13(2)	2(1)
C(47)	42(2)	21(2)	25(2)	3(1)	21(2)	-1(2)
C(48)	24(2)	18(2)	12(2)	4(1)	8(1)	3(1)
C(49)	27(2)	23(2)	23(2)	1(1)	12(1)	-1(1)
C(50)	44(2)	18(2)	32(2)	4(2)	21(2)	2(2)
C(51)	44(2)	28(2)	32(2)	14(2)	20(2)	16(2)
C(52)	26(2)	44(2)	23(2)	7(2)	7(2)	9(2)
C(53)	27(2)	26(2)	22(2)	1(1)	9(1)	0(1)
C(54)	27(2)	20(2)	22(2)	-2(1)	11(1)	-1(1)
C(55)	24(2)	27(2)	36(2)	-7(2)	6(2)	0(1)
C(56)	46(2)	20(2)	39(2)	-5(2)	23(2)	-12(2)
C(57)	28(2)	12(2)	21(2)	1(1)	5(1)	-2(1)
C(58)	34(2)	27(2)	30(2)	-11(2)	3(2)	1(2)
C(59)	26(2)	35(2)	41(2)	-5(2)	1(2)	5(2)
C(60)	35(2)	22(2)	41(2)	4(2)	17(2)	9(2)
C(61)	42(2)	24(2)	27(2)	-4(2)	10(2)	5(2)
C(62)	28(2)	23(2)	23(2)	-3(1)	3(1)	2(1)
C(63)	41(2)	16(2)	19(2)	0(1)	13(2)	3(1)

C(64)	49(2)	22(2)	19(2)	2(1)	10(2)	0(2)
C(65)	46(2)	23(2)	12(2)	4(1)	5(2)	0(2)
C(66)	50(2)	39(2)	19(2)	-3(2)	11(2)	-2(2)
C(67)	58(3)	68(3)	21(2)	0(2)	9(2)	25(2)
C(68)	108(4)	36(2)	21(2)	-5(2)	4(2)	31(3)
C(69)	111(4)	27(2)	18(2)	2(2)	-3(2)	-15(2)
C(70)	72(3)	34(2)	15(2)	2(2)	5(2)	-16(2)
C(71)	30(2)	27(2)	27(2)	-8(2)	14(2)	-10(1)
C(72)	20(2)	37(2)	22(2)	-7(2)	6(1)	-2(1)
C(73)	35(2)	30(2)	29(2)	-4(2)	12(2)	6(2)
C(74)	40(2)	40(2)	25(2)	6(2)	12(2)	9(2)
C(75)	27(2)	27(2)	30(2)	-5(2)	12(2)	-6(1)
C(76)	36(2)	36(2)	37(2)	2(2)	7(2)	-12(2)

Hydrogen coordinates (x 10^4) and isotropic displacement parameters ($Å^2$x 10^3) for znetonnph.

	x	y	z	U(eq)
H(3A)	3225	5189	9256	23
H(5A)	3399	5854	7838	25
H(8A)	708	4386	8854	38
H(8B)	772	3655	9068	38
H(8C)	857	3794	8487	38
H(9A)	2119	4664	9639	42
H(9B)	3208	4286	9759	42
H(9C)	2176	3917	9817	42
H(11A)	4275	3671	9358	29
H(12A)	5125	2664	9420	36
H(13A)	4171	1704	9122	38
H(14A)	2373	1779	8761	34
H(15A)	1528	2790	8689	27
H(17A)	4543	5823	9590	47
H(17B)	5101	6500	9530	47
H(17C)	5363	5864	9236	47
H(18A)	3821	6826	8188	54
H(18B)	4922	6471	8384	54
H(18C)	4662	7110	8674	54
H(20A)	1779	6315	8525	36
H(21A)	506	6958	8776	45
H(22A)	992	7720	9450	48
H(23A)	2754	7856	9857	50
H(24A)	4036	7238	9594	37
H(25A)	1598	4887	7129	28
H(25B)	2561	5331	7084	28
H(26A)	2712	4917	6286	36
H(26B)	1629	4574	6288	36
H(28A)	3962	4354	5870	46
H(29A)	4335	3501	5342	58
H(30A)	3187	2609	5136	58
H(31A)	1677	2572	5452	51
H(32A)	1343	3401	6005	41

H(33A)	4233	4783	7203	33
H(33B)	4228	4203	6800	33
H(34A)	5133	3818	7599	35
H(34B)	4246	4060	7873	35
H(35A)	4949	2742	7155	52
H(35B)	4087	3110	6724	52
H(35C)	3810	2436	6961	52
H(36A)	4858	2708	8031	58
H(36B)	3703	2417	7901	58
H(36C)	3959	3074	8231	58
H(37A)	531	3000	6714	30
H(37B)	1438	2475	6734	30
H(38A)	264	2082	7234	52
H(38B)	484	2718	7592	52
H(38C)	1388	2194	7612	52
H(41A)	1867	8111	5735	24
H(43A)	1451	7498	7129	27
H(46A)	4145	9530	6576	37
H(46B)	4333	9682	6015	37
H(46C)	4397	8948	6224	37
H(47A)	3054	9349	5212	41
H(47B)	2031	8948	5238	41
H(47C)	3152	8615	5421	41
H(49A)	3436	10559	6247	28
H(50A)	2489	11536	6093	36
H(51A)	713	11527	5685	39
H(52A)	-145	10522	5453	37
H(53A)	804	9541	5601	29
H(55A)	-339	7445	5727	44
H(55B)	-65	6796	5453	44
H(55C)	499	7467	5382	44
H(56A)	1185	6492	6793	49
H(56B)	382	6192	6300	49
H(56C)	70	6823	6582	49
H(58A)	3154	6944	6598	37
H(59A)	4538	6357	6407	42
H(60A)	4262	5695	5660	38
H(61A)	2583	5613	5122	37

H(62A)	1201	6211	5307	30
H(63A)	3197	8465	7876	29
H(63B)	2198	8040	7894	29
H(64A)	3007	8817	8695	36
H(64B)	1897	8481	8641	36
H(66A)	580	9022	9003	43
H(67A)	88	9861	9496	59
H(68A)	1187	10775	9731	69
H(69A)	2747	10850	9459	66
H(70A)	3220	10013	8948	49
H(71A)	489	9214	8072	32
H(71B)	531	8632	7676	32
H(72A)	672	9357	7020	32
H(72B)	-263	9624	7250	32
H(73A)	-84	10695	7695	46
H(73B)	1057	10974	7938	46
H(73C)	687	10311	8155	46
H(74A)	186	10724	6846	52
H(74B)	1105	10322	6693	52
H(74C)	1362	10975	7028	52
H(75A)	3461	10890	8217	33
H(75B)	4352	10347	8264	33
H(76A)	4666	11219	7714	55
H(76B)	3544	11104	7335	55
H(76C)	4431	10562	7382	55
